# Contextual auditory processing in the inferior colliculus is affected in a sex- and age-dependent manner in the valproic acid-induced rat model of autism

**DOI:** 10.1371/journal.pbio.3003309

**Published:** 2025-08-04

**Authors:** Sara Cacciato-Salcedo, Ana B. Lao-Rodríguez, Manuel S. Malmierca

**Affiliations:** 1 Cognitive and Auditory Neuroscience Laboratory (CANELAB), Institute of Neuroscience of Castilla y León (INCYL), Salamanca, Spain; 2 Salamanca Institute for Biomedical Research (IBSAL), Salamanca, Spain; 3 Department of Cell Biology and Pathology, Faculty of Medicine, University of Salamanca, Salamanca, Spain; University College London, UNITED KINGDOM OF GREAT BRITAIN AND NORTHERN IRELAND

## Abstract

Diverse biological factors, such as sex and age, confer heterogeneity on sensory processing challenges in autism. These factors result in major difficulties in the processing of contextual information in social and non-social situations. To assess divergence in autistic traits, it is critical to consider sex- and age-related variability. Nevertheless, these differences remain largely elusive. Animal models of autism offer the possibility to examine contextual processing at the single-neuron level. Here, we investigated predictive processing of contextual auditory cues in the auditory midbrain of control and prenatally valproic acid-induced rats, a well-established animal model of autism. The rats were prepubertal and adult female and male animals. We performed single-unit recordings in the inferior colliculus of control and prenatally, or *in utero*, exposed rats under the classical oddball paradigm and non-repetitive cascade control sequences to study neuronal mismatch. This is the neuronal correlate of mismatch negativity, the brain’s automatic response to interruptions in environmental regularity. When comparing control and exposed rats, our results demonstrated a reduction in neuronal mismatch in rats exposed to valproic acid. However, exposed adult females exhibited an increased neuronal mismatch compared to their control counterparts. With respect to sex distinctions, valproic acid induced sex differences in neuronal mismatch of prepubertal and adult rats that are not observable in control animals. Moreover, we detected an age-dependent refinement in prediction error that is not affected by the drug. But valproic acid altered typical developmental trajectory of neuronal mismatch in both sexes. Such observations support sex- and age-related effects of *in utero* valproic acid exposure in contextual auditory processing at the neural level of the inferior colliculus. In autism, atypical predictive processing of environmental regularities underlies unusual responses to novel experiences. The present study highlights the importance of sex and age, that confer heterogeneity to these challenges.

## Abstract 2

Biological factors like sex and age affect sensory processing, particularly interpretation of contextual cues, in autism spectrum disorder. This study shows that prenatal exposure to valproic acid in rats, a model with autistic-like features, alters predictive auditory processing, revealing sex- and age-specific neural differences that mirror the heterogeneity seen in autistic traits.

## Introduction

Our perception is constantly interrupted by new and unexpected signals from the surrounding environment. The brain creates an internal representation of the world by extracting patterns from the sensory systems. This enables it to respond to changes in sensory inputs effectively [1,2]. In autism spectrum disorder, hereafter referred to as *autism*, this mental structure of environmental regularity is divergent from typical representations and, as such, compromises adaptive behavior towards unexpected events [3].

Autism is an early-onset neurodevelopmental condition characterized by atypical social communication and interaction, as well as restrictive and repetitive interests [4–6]. Given their ubiquity among autistic individuals, unusual sensory-based behaviors emerge as diagnostic criteria, especially in the auditory domain [7,8]. Auditory sensitivities, including, hyper-sensitivity to certain sounds, impair the extraction of environmental regularities and responsiveness to novel events [9], hindering behavioral navigation in social and non-social situations [3,7,10,11]. Indeed, autism-related changes along the auditory pathway have been demonstrated to impact the processing of contextual auditory cues [7,9,12–14]. But developmental trajectories with respect to sex disparities remain largely unexplored [15,16].

At the neural level, the capacity to detect changes in regular auditory scenes is reflected in the electroencephalogram as a mismatch negativity evoked response (MMN) [17]. MMN is currently best explained under the predictive coding framework [2,18]. This theory postulates that the brain generates internal models in a hierarchical manner based on environmental regularity [2, 19,20]. Higher-order brain areas send top–down predictions that are compared to bottom-up sensory inputs in lower-order brain regions. If a mismatch occurs, a prediction error emerges, and the internal models update. Thus, MMN represents a prediction error [2,18,19,21]. The predictive coding theory of autism provides a unifying framework to explain core symptoms [3,8,9,11,22–28]. It posits that atypical processing of sensory contexts contributes to unusual sensory sensitivities, and vice versa.

Diverse environmental exposures influence the occurrence of autism [29]. For example, the use of the antiepileptic valproic acid (VPA) drug during pregnancy increases the risk of autism in newborns up to 10-fold [29]. Prenatal VPA administration is considered as a standardized, robust model of autism-like symptoms in rats [30,31]. The auditory brain of VPA rats shows abnormalities resembling the atypical auditory system of idiopathic autism, including midbrain hypoplasia and dysmorphology [32–34]. The male-to-female ratio of autism incidence ranges from 4:1 in children to 2.6:1 in adults [35]. Changes in this ratio over the life span are associated with the developmental trajectories of the brain with respect to sex in autism [36]. However, the developmental trajectory of brainstem or midbrain structure and function is elusive in females and males with autism, as well as in the VPA rat model.

Studies on animal models are starting to consider sex and age as variables providing heterogeneity across autism-like symptoms [37,38]. For VPA rats, the literature on age-dependent changes in context processing is sparse, follows a single-sex approach, and is usually conducted at the behavioral level [31].

**Fig 1:**
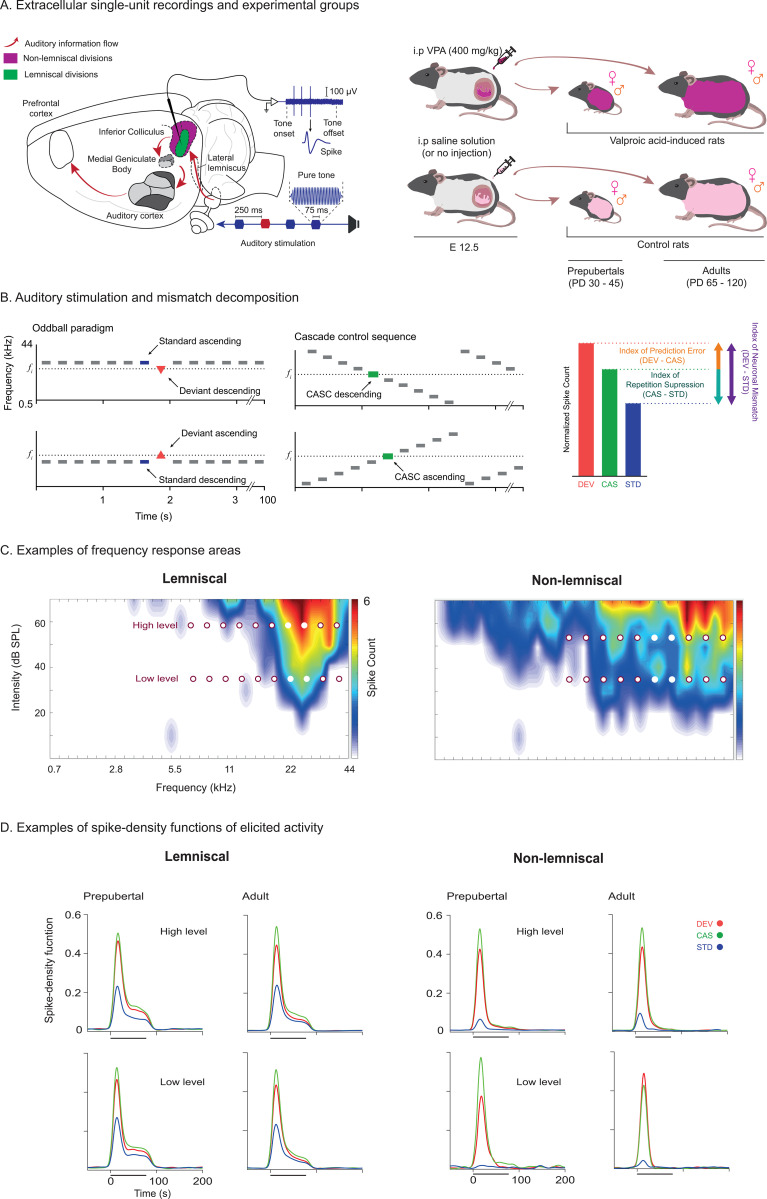
Experimental design and recording setup in the rat inferior colliculus. **A.** Left: Schematic of extracellular single-unit recordings in the lemniscal (green) and non-lemniscal (purple) regions of the IC in response to pure-tone auditory stimulation (red and blue). Right: Diagram of experimental group generation. Pregnant rats received an intraperitoneal injection of saline (control) or 400 mg/kg VPA on gestational day 12.5. An additional control group consisted of untreated rats acquired prior to the experiment. We examined the effects of sex, age, and prenatal VPA exposure by recording from female and male rats at prepubertal (P30–45) and adult (P65–120) stages. **B.** Left: Pure-tone stimulation paradigms included ascending and descending oddball sequences and a cascade control, corresponding to DEV, CAS, and STD conditions. Right: These conditions were decomposed to compute predictive processing components: neuronal mismatch (iMM, DEV – STD), repetition suppression (iRS, CAS – STD), and prediction error (iPE, DEV – CAS). Neuronal mismatch was also obtained by summing iRS and iPE. **C.** Representative frequency response areas from lemniscal and non-lemniscal IC, with dots marking the tone frequencies used in low- and high-intensity conditions (see “Materials and methods”). **D.** Spike-density functions to DEV, CAS, and STD conditions across lemniscal and non-lemniscal IC regions, showing usual responses in prepubertal and adult animals at both sound levels. Abbreviations: IC, inferior colliculus; VPA, valproic acid; DEV, deviant; CAS, cascade; STD, standard; iMM, neuronal mismatch index; iRS, repetition suppression index; iPE, prediction error index. See S1 Data (for data from panel D).

Animal models offer the unique opportunity to analyze neuronal activity at the cellular level with high spatial resolution. They provide evidence for specific prediction-generating circuits that influence the sensory representations in early brain regions [27], such as the inferior colliculus (IC) of the auditory midbrain (Fig 1A).

To this end, we presented the classical oddball paradigm and cascade control (CAS) sequences to study neuronal mismatch, the neural correlate of MMN [39, 40], in typically developing, or *control*, and VPA rats (Fig 1A and 1B). In the classical oddball paradigm, low-probability deviant (DEV) tones interrupt the sequence of high-probability standard (STD) tones. The difference in neural activity between DEV and STD conditions represents a neuronal mismatch (Fig 1B) [38,41]. Tones in the cascade control sequences are equivalent to DEV events with respect to neural adaptation but do not disrupt regularity [39]. Hence, the difference between DEV and CAS conditions reveal a prediction error effect, or the incongruence between the internal model and incoming sensory information (Fig 1B). Meanwhile, the difference between CAS and STD conditions reflects a repetition suppression effect, or neural adaptation upon stimulus repetition (Fig 1B). Thus, neuronal mismatch represents both prediction error and repetition suppression phenomena.

We performed single-unit recordings in the IC, as neuronal mismatch first emerges at this level [41–46]. Since non-lemniscal IC neurons of rats increase prediction error signaling to low-intensity sounds [40,41], we inspected the processing of auditory contexts at high- and low-intensity sounds. Further, it has been suggested that autism is associated with divergent involvement of the ‘non-classical’ (*i.e.*, non-lemniscal) auditory pathways [47]. Thus, we assessed the lemniscal and non-lemniscal IC of control and VPA rats. Prenatal exposure to VPA in pregnant rats induced autism-like neuronal signatures in the offspring. After the litter is born, we allowed the animals to develop until prepuberty or adulthood before performing the electrophysiological recordings. Then, we investigated neuronal mismatch, repetition suppression, and prediction error using a hierarchical statistical approach in control and VPA rats. We first applied generalized linear mixed-effects models to assess the main effects of sex, age, exposure, division, and sound level, along with their interactions. We subsequently performed post hoc population- and group-level comparisons across each division and sound level to identify fine-grained differences.

## Results

To investigate how sex, age, and prenatal VPA exposure influence predictive processing in the inferior colliculus, we recorded single-neuron auditory responses using the oddball paradigm and cascade control sequences (Fig 1B). We obtained data from 903 neurons across lemniscal and non-lemniscal divisions in urethane-anesthetized rats (Table 1): 397 from controls (prepubertal females: *n* = 9 animals, 102 neurons; prepubertal males: *n* = 11 animals, 72 neurons; adult females: *n* = 9 animals, 111 neurons; adult males: *n* = 10 animals, 112 neurons) and 506 from VPA-exposed animals (prepubertal females: *n* = 12 animals, 125 neurons; prepubertal males: *n* = 11 animals, 160 neurons; adult females: *n* = 10 animals, 80 neurons; adult males: *n* = 11 animals, 141 neurons). Developmental stages followed Sengupta [48], with prepuberty spanning 30–45 postnatal days (P) and adulthood P65–120.

**Table 1:** Median normalized responses in control and valproic acid-induced rats. Spike count to deviant, cascade and standard conditions and predictive components of neuronal mismatch, repetition suppression and prediction error in the lemniscal and non-lemniscal inferior colliculus at high- and low-intensity sounds.

	Control								VPA							
	Female				Male				Female				Male			
	Prepubertal		Adult		Prepubertal		Adult		Prepubertal		Adult		Prepubertal		Adult	
	IC_L_	IC_NL_	IC_L_	IC_NL_	IC_L_	IC_NL_	IC_L_	IC_NL_	IC_L_	IC_NL_	IC_L_	IC_NL_	IC_L_	IC_NL_	IC_L_	IC_NL_
**High-intensity sound stimulation (≥40 dB SPL)**																
Neurons	28	45	21	47	24	32	38	46	56	53	29	42	41	81	92	18
Points	80	225	125	368	66	160	100	180	177	229	83	158	122	336	277	100
DEV	0.6068	0.6421	0.5579	0.6558	0.6011	0.6430	0.6027	0.6335	0.5820	0.5989	0.6086	0.6537	0.6049	0.6329	0.5930	0.6342
STD	0.3507	0.0510	0.4315	0.2307	0.3778	0.0605	0.3678	0.0372	0.4192	0.0842	0.4130	0.0923	0.3832	0.1047	0.3260	0.1010
CAS	0.6913	0.7543	0.6563	0.6582	0.7001	0.7382	0.7063	0.7652	0.6888	0.7812	0.6768	0.7323	0.6854	0.7427	0.7211	0.7411
iMM	0.2561	0.5911	0.1264	0.4251	0.2233	0.5824	0.2349	0.5963	0.1628	0.5147	0.1956	0.5614	0.2218	0.5282	0.2670	0.5332
*p*-value	***0.000***	***0.000***	***0.000***	***0.000***	***0.000***	***0.000***	***0.000***	***0.000***	***0.000***	***0.000***	***0.000***	***0.000***	***0.000***	***0.000***	***0.000***	***0.000***
iPE	−0.0844	−0.1122	−0.0984	−0.0024	−0.0990	−0.0953	−0.1036	−0.1317	−0.1068	−0.1823	−0.0682	−0.0786	−0.0804	−0.1098	−0.1281	−0.1069
*p*-value	***0.000***	***0.000***	***0.000***	***0.000***	***0.000***	***0.000***	***0.000***	***0.000***	***0.000***	***0.000***	***0.000***	***0.000***	***0.000***	***0.000***	***0.000***	***0.000***
iRS	0.3405	0.7033	0.2248	0.4275	0.3223	0.6777	0.3385	0.7280	0.2696	0.6971	0.2638	0.6400	0.3022	0.6380	0.3951	0.6401
*p*-value	***0.000***	***0.000***	***0.000***	0.9412	***0.000***	***0.000***	***0.000***	***0.000***	***0.000***	***0.000***	***0.000***	***0.000***	***0.000***	***0.000***	***0.000***	***0.000***
**Low-intensity sound stimulation (<40 dB SPL)**																
Neurons	22	7	6	37	10	6	6	22	10	6	2	7	22	16	19	12
Points/required	72	14	22	209	37	39	50	92	40	12	8	32	105	57	73	124
DEV	0.6315	0.5458	0.6275	0.7204	0.5954	0.6009	0.6171	0.7149	0.6013	0.5868	0.5942	0.7333	0.6261	0.6438	0.6220	0.7129
STD	0.3414	0.0494	0.4547	0.1029	0.3354	0.0478	0.3647	0.0437	0.3296	0.0470	0.3474	0.0578	0.3100	0.0888	0.2968	0.0654
CAS	0.6886	0.8342	0.6410	0.6376	0.7065	0.7949	0.6757	0.6739	0.7115	0.8072	0.7192	0.6652	0.6877	0.7579	0.7161	0.6955
iMM	0.2902	0.4964	0.1728	0.6175	0.2600	0.5531	0.2525	0.6712	0.2717	0.5398	0.2467	0.6754	0.3161	0.5550	0.3204	0.6474
*p*-value	***0.000***	***0.0013***	***0.000***	***0.000***	***0.000***	***0.000***	***0.000***	***0.000***	***0.000***	***0.0080***	***0.0244***	***0.000***	***0.000***	***0.000***	***0.000***	***0.000***
iPE	−0.0571	−0.2884	−0.0136	0.0829	−0.1111	−0.1940	−0.0585	0.0409	−0.1102	−0.2204	−0.1250	0.0680	−0.0616	−0.1141	−0.1217	0.0173
*p*-value	***0.000***	0.1306	0.7630	***0.0062***	***0.0037***	***0.0046***	***0.0164***	***0.0325***	***0.000***	***0.0412***	0.1336	0.0801	***0.000***	***0.000***	***0.000***	0.2530
iRS	0.3472	0.7848	0.1863	0.5347	0.3711	0.7471	0.3110	0.6302	0.3818	0.7602	0.3717	0.6074	0.3777	0.6691	0.4422	0.6301
*p*-value	***0.000***	***0.000***	***0.000***	***0.000***	***0.000***	***0.000***	***0.000***	***0.000***	***0.000***	***0.000***	***0.000***	***0.000***	***0.000***	***0.000***	***0.000***	***0.000***

“Neurons” indicates the number of single units contributing to each group-level estimate. “Points” refers to total frequency pairs recorded. DEV, CAS, and STD represent median normalized responses to deviant, cascade, and standard conditions, respectively. iMM = DEV – STD; iPE = DEV – CAS; iRS = CAS – STD. *P*-values for iMM, iPE, and iRS were calculated using Friedman tests with false discovery rate correction across each division and sound level. Data are grouped by sound intensity (high vs. low sound levels), sex, age, exposure (control or VPA), and IC division (lemniscal: IC_L_, and non-lemniscal: IC_NL_).

After isolating single neurons, we first recorded the frequency response area, followed by the presentation of pure-tone sequences with fixed frequencies and sound intensities (Fig 1C). Cascade sequences comprised 10 frequencies spaced at 0.5-octave intervals, while oddball sequences used two frequency pairs. Both sequence types were presented in ascending and descending versions at high (≥40 dB SPL) and low (<40 dB SPL) sound levels (Fig 1C). Each sequence contained 400 tones delivered at a rate of 4 Hz.

We first quantified spontaneous firing before sound presentation. Next, we computed average spike counts for the DEV (rare, 10%), STD (frequent, 90%), and CAS conditions (Fig 1D; S1 Data). From these, we derived the index of neuronal mismatch (iMM), calculated as the difference between DEV and STD responses (Fig 1B). We further decomposed iMM into repetition suppression (iRS; STD − CAS) and prediction error (iPE; DEV − CAS) indices (Fig 1B). These indices quantified adaptation to repetition and the enhancement of DEV responses associated with predictive processes (i.e., genuine deviance detection). Finally, we computed Spearman correlations between spontaneous activity and iMM, applying false discovery rate correction across all tests.

**Fig 2:**
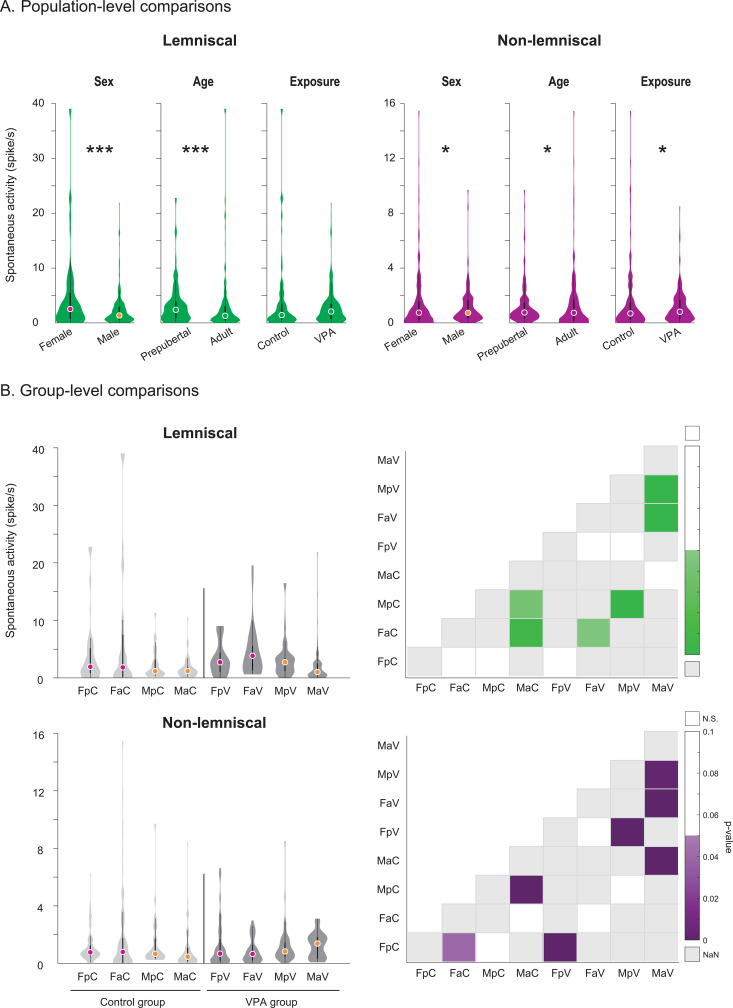
Population- and group-level comparisons of spontaneous activity in the inferior colliculus. **A.** Population-level comparisons of sex, age, and exposure effects within the lemniscal and non-lemniscal divisions of the IC. **B.** Group-level comparisons across experimental subgroups defined by sex, age, and prenatal exposure. Violin plots show spontaneous firing rates in control (light grey) and VPA-exposed (dark grey) rats. Medians are marked in pink for females and orange for males. Heatmaps display statistical significance of pairwise comparisons using the Wilcoxon rank-sum test with Bonferroni correction (*p < 0.05, **p = 0.001, ***p < 0.001). A green gradient represents significance levels within the lemniscal division, and a purple gradient represents those within the non-lemniscal division. Non-significant results are shown in white (N.S.), and missing comparisons in grey (NaN). Group abbreviations: FpC = female prepubertal control, FaC = female adult control, MpC = male prepubertal control, MaC = male adult control, FpV = female prepubertal VPA, FaV = female adult VPA, MpV = male prepubertal VPA, MaV = male adult VPA. General abbreviations: IC, inferior colliculus; VPA, valproic acid. See S2 Data.

**Fig 3:**
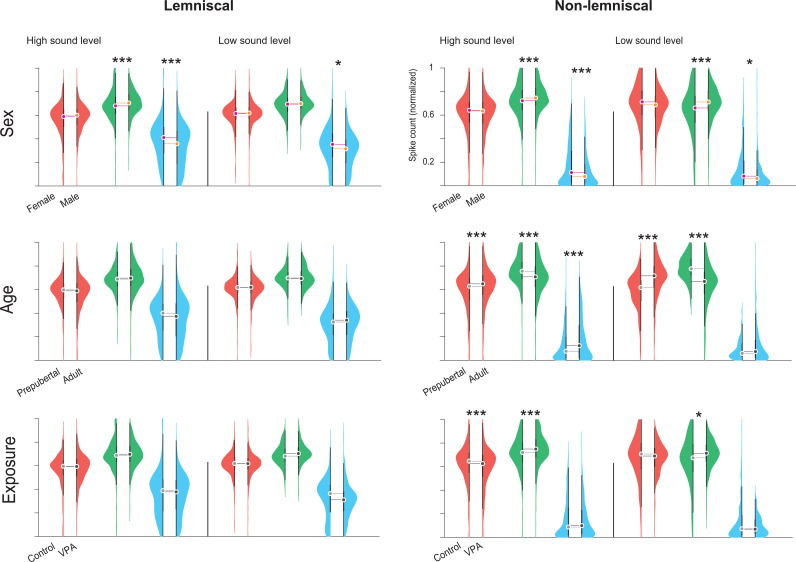
Population-level comparisons of spike count to deviant, cascade, and standard conditions. Violin plots show normalized spike counts in response to DEV (red), CAS (green), and STD (blue) auditory conditions. Left panels correspond to the lemniscal division, and right panels to the non-lemniscal division of the IC. A solid vertical black line separates responses elicited at high sound levels (≥40 dB SPL) from those at low sound levels (<40 dB SPL). The upper row displays sex effects, the middle row shows age effects, and the bottom row illustrates exposure effects. Statistical significance of pairwise comparisons was assessed using the Wilcoxon rank-sum test with Bonferroni correction (*p < 0.05, **p = 0.001, ***p < 0.001). Abbreviations: IC, inferior colliculus; VPA, valproic acid; DEV, deviant; CAS, cascade; STD, standard. See S3 Data.

**Fig 4:**
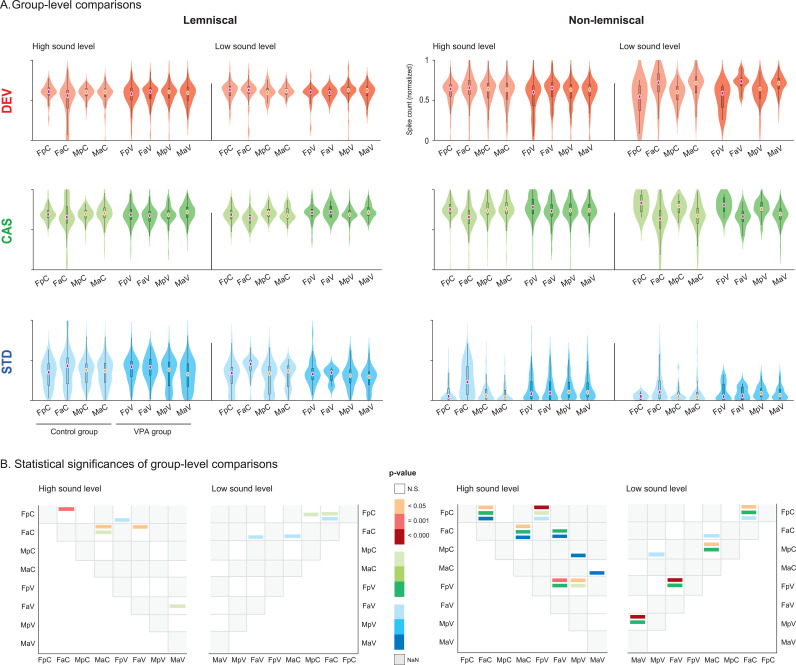
Group-level comparisons of spike count to deviant, cascade, and standard conditions. **A.** Violin plots show normalized spike counts in response to DEV (red), CAS (green), and STD (blue) conditions across experimental groups. Left panels correspond to the lemniscal division, and right panels to the non-lemniscal division of the IC. A solid vertical black line separates responses elicited at high sound levels (≥40 dB SPL) from those at low sound levels (<40 dB SPL). Group abbreviations: FpC = female prepubertal control, FaC = female adult control, MpC = male prepubertal control, MaC = male adult control, FpV = female prepubertal VPA, FaV = female adult VPA, MpV = male prepubertal VPA, MaV = male adult VPA. General abbreviations: IC, inferior colliculus; VPA, valproic acid; DEV, deviant; CAS, cascade; STD, standard. **B.** Heatmaps show statistical significance of pairwise group comparisons based on the Wilcoxon rank-sum test with false discovery rate correction (*p < 0.05, **p = 0.001, ***p < 0.001). As significance strengthens, the corresponding color becomes darker: red for DEV, green for CAS, and blue for STD. Non-significant comparisons are shown in white (N.S.), and missing comparisons in grey (NaN). See S4 Data.

**Fig 5:**
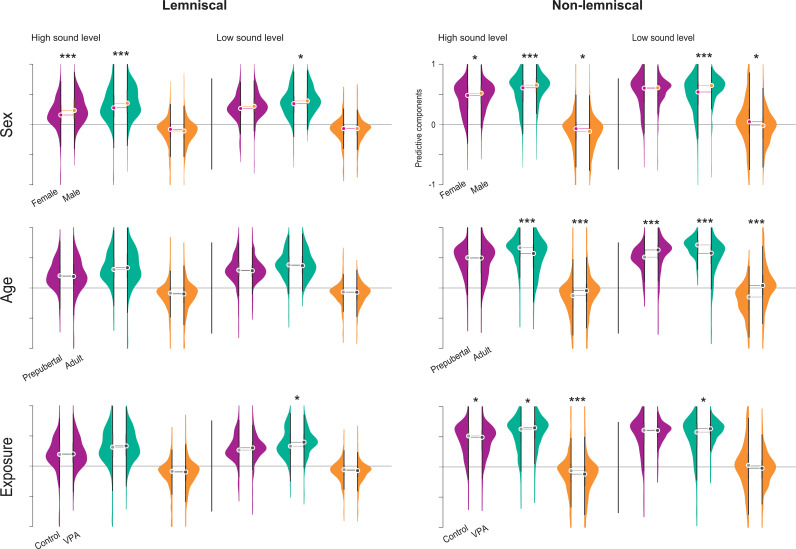
Population-level comparisons of predictive components of mismatch processing. Violin plots show normalized values for iMM (purple), iRS (turquoise), and iPE (orange). Left panels correspond to the lemniscal division, and right panels to the non-lemniscal division of the IC. A solid vertical black line separates responses elicited at high sound levels (≥40 dB SPL) from those at low sound levels (<40 dB SPL). The upper row displays sex effects, the middle row shows age effects, and the bottom row illustrates exposure effects. Statistical significance of pairwise comparisons was assessed using the Wilcoxon rank-sum test with Bonferroni correction (*p < 0.05, **p = 0.001, ***p < 0.001). Abbreviations: IC, inferior colliculus; VPA, valproic acid; iMM, neuronal mismatch index; iRS, repetition suppression index; iPE, prediction error index. See S5 Data.

**Fig 6:**
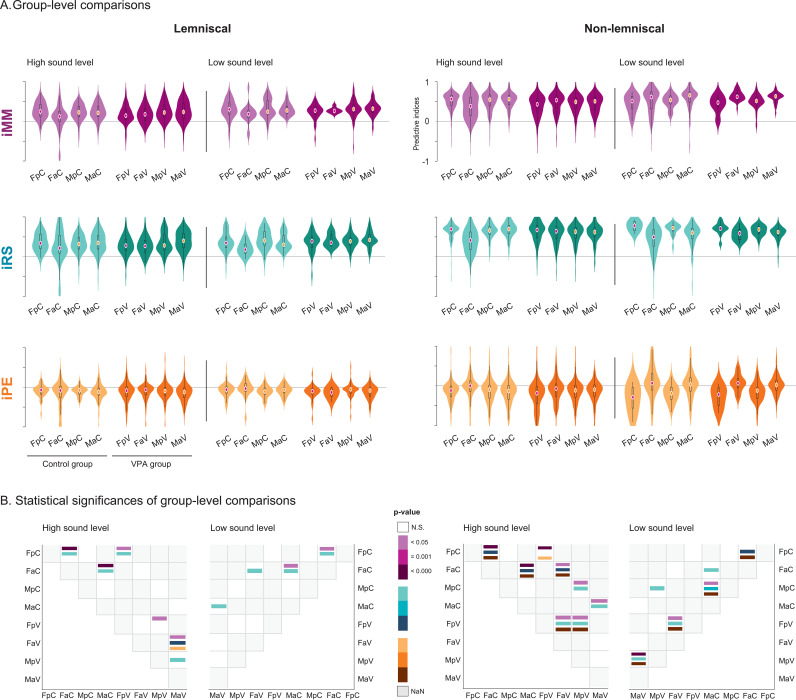
Group-level comparisons of spike count to predictive components of mismatch processing. **A.** Violin plots show normalized values for iMM (purple), iRS (turquoise), and iPE (orange) across experimental groups. Left panels correspond to the lemniscal division, and right panels to the non-lemniscal division of the IC. A solid vertical black line separates responses elicited at high sound levels (≥40 dB SPL) from those at low sound levels (<40 dB SPL). Group abbreviations: FpC = female prepubertal control, FaC = female adult control, MpC = male prepubertal control, MaC = male adult control, FpV = female prepubertal VPA, FaV = female adult VPA, MpV = male prepubertal VPA, MaV = male adult VPA. General abbreviations: IC, inferior colliculus; VPA, valproic acid; iMM, neuronal mismatch index; iRS, repetition suppression index; iPE, prediction error index. **B.** Heatmaps show statistical significance of pairwise group comparisons based on the Wilcoxon rank-sum test with false discovery rate correction (*p < 0.05, **p = 0.001, ***p < 0.001). As significance strengthens, the corresponding color becomes darker: purple for iMM, turquoise for iRS, and orange for iPE. Non-significant comparisons are shown in white (N.S.), and missing comparisons in grey (NaN). See S6 Data.

We assessed the effects of sex, age, and VPA exposure using generalized linear mixed-effects models fit separately for spontaneous activity, evoked responses, and predictive indices. The models included random intercepts and slopes to account for subject variability (*n* = 83). We evaluated model performance using intraclass correlation coefficients, cross-validated median absolute errors, and bootstrap confidence intervals for fixed effects (Tables 2, 3, and 4). To examine the effects of sex, age, and exposure at the population level, we applied Wilcoxon rank sum tests with Bonferroni correction. At the experimental group level, we conducted Wilcoxon rank sum tests with false discovery rate correction. We compared spontaneous activity within each division (Fig 2; S2 Data) and assessed evoked responses and predictive indices by division and sound level (Figs 3, 4, 5, and 6; S3, S4, S5, and S6 Data).

**Table 2:** Generalized linear mixed-effect model outcome for spontaneous neuronal activity in the inferior colliculus.

Term	Estimate	SE	*t*-Stat	*DF*	*p-*value	95% CI [Lower, Upper]
**Spontaneous activity**						
**(Intercept)**	8.040	1.372	5.859	95	***<0.001***	[0.310, 19.856]
**Division**	−7.061	1.373	−5.143	95	***<0.001***	[–18.318, 1.301]
**Sex**	−7.161	1.725	−4.152	95	***<0.001***	[–18.831, 0.815]
**Age**	−6.135	1.748	−3.510	95	***<0.001***	[–18.024, 1.881]
**Exposure**	−6.443	1.617	−3.984	95	***<0.001***	[–18.218, 1.745]
**Division × Sex**	6.925	1.652	4.192	95	***<0.001***	[–1.558, 17.992]
**Division × Age**	6.155	1.660	3.707	95	***<0.001***	[–2.217, 17.725]
**Sex × Age**	8.355	2.336	3.577	95	***<0.001***	[0.248, 19.292]
**Division × Exposure**	6.199	1.731	3.581	95	***<0.001***	[–2.453, 17.733]
**Sex × Exposure**	6.707	2.010	3.336	95	***0.001***	[–1.658, 18.264]
**Age × Exposure**	6.607	2.048	3.226	95	***0.002***	[–1.808, 18.651]
**Division × Sex × Age**	−8.073	2.149	−3.756	95	***<0.001***	[–18.615, 0.124]
**Division × Sex × Exposure**	−6.251	2.119	−2.950	95	***0.004***	[–17.111, 2.550]
**Division × Age × Exposure**	−6.308	2.128	−2.964	95	***0.004***	[–18.170, 2.256]
**Sex × Age × Exposure**	−7.983	2.746	−2.907	95	***0.005***	[–19.067, 0.874]
**Division × Sex × Age × Exposure**	7.600	2.834	2.682	95	***0.009***	[–1.117, 18.378]

The table presents *generalized linear mixed-effects model* results with the following abbreviations: Term refers to the fixed effect or interaction; *Estimate* denotes the estimated coefficient; *SE* is the standard error; *t-Stat* represents the *t*-statistic; *DF* stands for degrees of freedom; *p-value* indicates the probability of observing the result under the null hypothesis; and *95% CI [Lower, Upper]* shows the 95% confidence interval bounds. Statistically significant effects (*p* < 0.05) are highlighted in bold.

**Table 3:** Generalized linear mixed-effect model outcome for deviant-, control-, and standard-elicited responses in the inferior colliculus.

Term	Estimate	SE	t-Stat	DF	*p-Value*	95% CI [Lower, Upper]
**DEV conditions**						
** (Intercept)**	–0.778	0.084	–9.221	139	***<0.001***	[–1.275, –0.411]
** Division**	0.321	0.085	3.770	139	***<0.001***	[0.039, 0.852]
** Level**	0.333	0.090	3.690	139	***<0.001***	[0.010, 1.421]
** Sex**	0.279	0.109	2.566	139	***0.011***	[–0.111, 0.767]
** Age**	0.299	0.116	2.579	139	***0.011***	[–0.074, 0.771]
** Exposure**	0.257	0.094	2.751	139	***0.007***	[–0.112, 0.766]
** **Division × Level	–0.152	0.090	–1.682	139	*0.095*	[–1.322, 0.088]
** Division × Sex**	–0.242	0.098	–2.461	139	***0.015***	[–0.775, 0.083]
** Level × Sex**	–0.258	0.128	–2.026	139	***0.045***	[–1.273, 0.206]
** Division × Age**	–0.291	0.101	–2.883	139	***0.005***	[–0.813, 0.021]
** **Level × Age	–0.243	0.124	–1.960	139	*0.052*	[–1.293, 0.205]
** **Sex × Age	–0.312	0.162	–1.928	139	*0.056*	[–0.823, 0.087]
** Division × Exposure**	–0.255	0.103	–2.481	139	***0.014***	[–0.829, 0.028]
** Level × Exposure**	–0.401	0.134	–2.993	139	***0.003***	[–1.539, –0.025]
** Sex × Exposure**	–0.289	0.119	–2.433	139	***0.016***	[–0.784, 0.117]
** Age × Exposure**	–0.309	0.126	–2.440	139	***0.016***	[–0.810, 0.081]
** **Division × Level × Sex	0.138	0.114	1.209	139	*0.229*	[–0.191, 1.308]
** **Division × Level × Age	–0.180	0.110	–1.625	139	*0.106*	[–0.680, 0.853]
** Division × Sex × Age**	0.272	0.129	2.115	139	***0.036***	[–0.077, 0.801]
** **Level × Sex × Age	0.210	0.181	1.161	139	*0.248*	[–0.300, 1.190]
** Division × Level × Exposure**	0.345	0.119	2.906	139	***0.004***	[0.068, 1.598]
** **Division × Sex × Exposure	0.180	0.127	1.420	139	*0.158*	[–0.196, 0.697]
** Level × Sex × Exposure**	0.398	0.173	2.299	139	***0.023***	[–0.064, 1.419]
** **Division × Age × Exposure	0.133	0.125	1.065	139	*0.289*	[–0.227, 0.676]
** **Level × Age × Exposure	0.232	0.177	1.317	139	*0.190*	[–0.198, 1.353]
** Sex × Age × Exposure**	0.349	0.175	1.995	139	***0.048***	[–0.050, 0.878]
** **Division × Level × Sex × Age	0.161	0.150	1.078	139	*0.283*	[–0.765, 0.809]
** **Division × Level × Sex × Exposure	–0.088	0.160	–0.548	139	*0.585*	[–1.267, 0.404]
** **Division × Level × Age × Exposure	0.056	0.153	0.369	139	*0.713*	[–1.053, 0.617]
** **Division × Sex × Age × Exposure	–0.104	0.168	–0.619	139	*0.537*	[–0.642, 0.269]
** **Level × Sex × Age × Exposure	–0.250	0.243	–1.030	139	*0.305*	[–1.277, 0.324]
** Division × Level × Sex × Age × Exposure**	–0.296	0.208	–1.423	139	***<0.001***	[–1.024, 0.779]
**CAS conditions**						
** (Intercept)**	–0.713	0.083	–8.554	139	***<0.001***	[–1.330, –0.438]
** Division**	0.245	0.080	3.070	139	***0.003***	[–0.011, 0.933]
** Level**	0.270	0.080	3.382	139	***0.001***	[–0.001, 1.609]
** Sex**	0.370	0.111	3.351	139	***0.001***	[0.098, 1.017]
** Age**	0.329	0.119	2.755	139	***0.007***	[0.056, 0.952]
** Exposure**	0.331	0.089	3.716	139	***<0.001***	[0.011, 0.962]
** Division × Level**	–0.236	0.075	–3.159	139	***0.002***	[–1.694, –0.084]
** Division × Sex**	–0.210	0.101	–2.074	139	***0.040***	[–0.935, 0.039]
** Level × Sex**	–0.315	0.110	–2.860	139	***0.005***	[–1.726, –0.043]
** **Division × Age	–0.139	0.108	–1.286	139	*0.200*	[–0.820, 0.127]
** Level × Age**	–0.248	0.114	–2.185	139	***0.031***	[–1.634, 0.028]
** Sex × Age**	–0.509	0.166	–3.057	139	***0.003***	[–1.111, –0.147]
** **Division × Exposure	–0.161	0.107	–1.504	139	*0.135*	[–0.837, 0.114]
** **Level × Exposure	–0.118	0.122	–0.966	139	*0.336*	[–1.498, 0.226]
** Sex × Exposure**	–0.315	0.117	–2.700	139	***0.008***	[–0.957, 0.009]
** Age × Exposure**	–0.314	0.126	–2.497	139	***0.014***	[–0.941, –0.012]
** Division × Level × Sex**	0.222	0.093	2.376	139	***0.019***	[0.133, 1.777]
** Division × Level × Age**	0.310	0.089	3.487	139	***0.001***	[0.059, 1.813]
** Division × Sex × Age**	0.334	0.147	2.269	139	***0.025***	[–0.035, 0.994]
** Level × Sex × Age**	0.461	0.162	2.848	139	***0.005***	[0.021, 1.872]
** **Division × Level × Exposure	0.036	0.098	0.365	139	*0.716*	[–0.211, 1.521]
** **Division × Sex × Exposure	0.201	0.137	1.466	139	*0.145*	[–0.119, 0.941]
** **Level × Sex × Exposure	0.136	0.156	0.872	139	*0.384*	[–0.207, 1.615]
** **Division × Age × Exposure	0.160	0.142	1.123	139	*0.263*	[–0.125, 0.823]
** **Level × Age × Exposure	0.113	0.163	0.690	139	*0.492*	[–0.288, 1.558]
** Sex × Age × Exposure**	0.435	0.175	2.490	139	***0.014***	[0.046, 1.042]
** Division × Level × Sex × Age**	–0.502	0.120	–4.193	139	***<0.001***	[–2.004, –0.027]
** **Division × Level × Sex × Exposure	–0.240	0.134	–1.796	139	*0.075*	[–1.824, 0.128]
** **Division × Level × Age × Exposure	–0.066	0.127	–0.516	139	*0.607*	[–1.600, 0.416]
** **Division × Sex × Age × Exposure	–0.318	0.197	–1.613	139	*0.109*	[–1.005, 0.138]
** **Level × Sex × Age × Exposure	–0.254	0.222	–1.145	139	*0.254*	[–1.797, 0.264]
** Division × Level × Sex × Age × Exposure**	0.424	0.173	2.455	139	***0.015***	[–0.268, 1.968]
**STD conditions**						
** (Intercept)**	0.612	0.068	9.042	139	***<0.001***	[0.422, 0.984]
** Division**	–0.379	0.073	–5.221	139	***<0.001***	[–0.732, –0.169]
** Level**	–0.201	0.065	–3.076	139	***0.003***	[–0.608, –0.022]
** Sex**	–0.282	0.085	–3.317	139	***0.001***	[–0.659, –0.068]
** Age**	–0.241	0.087	–2.771	139	***0.006***	[–0.637, –0.020]
**Exposure**	–0.203	0.081	–2.500	139	***0.014***	[–0.572, 0.030]
** **Division × Level	0.125	0.077	1.627	139	*0.106*	[–0.049, 0.546]
** **Division × Sex	0.117	0.091	1.290	139	*0.199*	[–0.119, 0.483]
** **Level × Sex	0.163	0.102	1.597	139	*0.113*	[–0.208, 0.712]
** **Division × Age	0.057	0.093	0.612	139	*0.542*	[–0.180, 0.431]
** **Level × Age	0.005	0.082	0.056	139	*0.955*	[–0.220, 0.483]
**Sex × Age**	0.415	0.119	3.495	139	***0.001***	[0.162, 0.804]
** **Division × Exposure	0.067	0.097	0.692	139	*0.490*	[–0.152, 0.426]
** **Level × Exposure	0.090	0.101	0.896	139	*0.372*	[–0.139, 0.483]
** **Sex × Exposure	0.181	0.100	1.810	139	*0.072*	[–0.095, 0.545]
**Age × Exposure**	0.217	0.103	2.117	139	***0.036***	[–0.043, 0.605]
** **Division × Level × Sex	–0.095	0.115	–0.826	139	*0.410*	[–0.647, 0.266]
** **Division × Level × Age	0.093	0.106	0.880	139	*0.380*	[–0.401, 0.301]
** **Division × Sex × Age	–0.231	0.127	–1.817	139	*0.071*	[–0.609, 0.046]
** **Level × Sex × Age	–0.239	0.128	–1.861	139	*0.065*	[–0.729, 0.184]
** **Division × Level × Exposure	–0.008	0.122	–0.064	139	*0.949*	[–0.446, 0.183]
** **Division × Sex × Exposure	0.062	0.125	0.492	139	*0.623*	[–0.262, 0.354]
** **Level × Sex × Exposure	–0.122	0.134	–0.909	139	*0.365*	[–0.708, 0.324]
** **Division × Age × Exposure	–0.001	0.123	–0.011	139	*0.991*	[–0.372, 0.251]
** **Level × Age × Exposure	0.054	0.124	0.436	139	*0.664*	[–0.433, 0.414]
**Sex × Age × Exposure**	–0.347	0.140	–2.486	139	***0.014***	[–0.767, –0.003]
** **Division × Level × Sex × Age	0.176	0.158	1.114	139	*0.267*	[–0.257, 0.699]
** **Division × Level × Sex × Exposure	–0.052	0.165	–0.314	139	*0.754*	[–0.449, 0.567]
** **Division × Level × Age × Exposure	–0.195	0.158	–1.230	139	*0.221*	[–0.512, 0.376]
** **Division × Sex × Age × Exposure	0.083	0.172	0.482	139	*0.631*	[–0.266, 0.562]
** **Level × Sex × Age × Exposure	0.132	0.171	0.773	139	*0.441*	[–0.341, 0.714]
** **Division × Level × Sex × Age × Exposure	0.036	0.219	0.164	139	*0.870*	[–0.678, 0.526]

The table presents *generalized linear mixed-effects model* results with the following abbreviations: *Term* refers to the fixed effect or interaction; *Estimate* denotes the estimated coefficient; *SE* is the standard error; *t-Stat* represents the *t*-statistic; *DF* stands for degrees of freedom; *p-value* indicates the probability of observing the result under the null hypothesis; and *95% CI [Lower, Upper]* shows the 95% confidence interval bounds. Statistically significant effects (*p* < 0.05) are highlighted in bold.

**Table 4:** Generalized linear mixed-effect model outcome for predictive components of mismatch processing in the inferior colliculus.

Term	Estimate	SE	t-Stat	DF	*p-Value*	95% CI [Lower, Upper]
**iMM**						
** **(Intercept)	–0.132	0.095	–1.387	139	*0.168*	[–0.320, 0.056]
** Division**	0.507	0.101	5.023	139	***<0.001***	[0.307, 0.706]
** Level**	0.360	0.092	3.915	139	***<0.001***	[0.178, 0.542]
** Sex**	0.403	0.120	3.358	139	***0.001***	[0.166, 0.641]
** Age**	0.391	0.125	3.133	139	***0.002***	[0.144, 0.638]
** Exposure**	0.340	0.111	3.065	139	***0.003***	[0.121, 0.559]
** **Division × Level	–0.177	0.102	–1.732	139	*0.085*	[–0.379, 0.025]
** **Division × Sex	–0.207	0.118	–1.751	139	*0.082*	[–0.441, 0.027]
** **Level × Sex	–0.257	0.137	–1.870	139	*0.064*	[–0.529, 0.015]
** **Division × Age	–0.200	0.120	–1.666	139	*0.098*	[–0.438, 0.037]
** **Level × Age	–0.140	0.118	–1.183	139	*0.239*	[–0.373, 0.094]
** Sex × Age**	–0.583	0.171	–3.399	139	***<0.001***	[–0.921, –0.244]
** **Division × Exposure	–0.226	0.125	–1.804	139	*0.073*	[–0.473, 0.022]
** Level × Exposure**	–0.397	0.139	–2.849	139	***0.005***	[–0.672, –0.121]
** Sex × Exposure**	–0.349	0.138	–2.530	139	***0.013***	[–0.622, –0.076]
** Age × Exposure**	–0.407	0.143	–2.837	139	***0.005***	[–0.691, –0.123]
** **Division × Level × Sex	0.130	0.140	0.927	139	*0.356*	[–0.148, 0.408]
** **Division × Level × Age	–0.168	0.130	–1.290	139	*0.199*	[–0.425, 0.089]
** Division × Sex × Age**	0.360	0.155	2.319	139	***0.022***	[0.053, 0.666]
** **Level × Sex × Age	0.352	0.179	1.964	139	*0.051*	[–0.002, 0.706]
** **Division × Level × Exposure	0.281	0.148	1.895	139	*0.060*	[–0.012, 0.574]
** **Division × Sex × Exposure	0.069	0.153	0.449	139	*0.654*	[–0.234, 0.371]
** Level × Sex × Exposure**	0.425	0.183	2.327	139	***0.021***	[0.064, 0.786]
** **Division × Age × Exposure	0.083	0.152	0.548	139	*0.585*	[–0.305, 0.384]
** **Level × Age × Exposure	0.201	0.175	1.148	139	*0.253*	[–0.197, 0.987]
** Sex × Age × Exposure**	0.597	0.196	3.046	139	***0.003***	[0.094, 0.923]
** **Division × Level × Sex × Age	–0.139	0.185	–0.751	139	*0.454*	[–0.962, 0.501]
** **Division × Level × Sex × Exposure	–0.099	0.196	–0.503	139	*0.615*	[–0.976, 0.403]
** **Division × Level × Age × Exposure	0.151	0.188	0.804	139	*0.423*	[–0.671, 0.591]
** **Division × Sex × Age × Exposure	–0.170	0.203	–0.837	139	*0.404*	[–0.576, 0.320]
** **Level × Sex × Age × Exposure	–0.428	0.240	–1.784	139	*0.077*	[–1.334, 0.256]
** **Division × Level × Sex × Age × Exposure	–0.054	0.254	–0.213	139	*0.831*	[–0.900, 0.954]
**iRS**						
** ** (Intercept)	–0.084	0.095	–0.885	139	*0.378*	[–0.864, 0.306]
** Division**	0.491	0.102	4.796	139	***<0.001***	[0.086, 1.266]
** Level**	0.354	0.089	3.969	139	***<0.001***	[–0.024, 1.111]
** Sex**	0.476	0.119	4.003	139	***<0.001***	[0.061, 1.263]
** Age**	0.415	0.121	3.428	139	***<0.001***	[–0.015, 1.220]
** Exposure**	0.335	0.116	2.889	139	***0.004***	[–0.108, 1.100]
** Division × Level**	–0.356	0.106	–3.349	139	***0.001***	[–1.152, 0.001]
** **Division × Sex	–0.228	0.128	–1.781	139	*0.077*	[–1.015, 0.185]
** Level × Sex**	–0.356	0.138	–2.569	139	***0.011***	[–1.318, 0.159]
** **Division × Age	–0.114	0.132	–0.869	139	*0.386*	[–0.901, 0.290]
** **Level × Age	–0.167	0.112	–1.487	139	*0.139*	[–0.917, 0.275]
** Sex × Age**	–0.662	0.166	–3.997	139	***<0.001***	[–1.473, –0.251]
** **Division × Exposure	–0.108	0.137	–0.788	139	*0.432*	[–0.879, 0.302]
** **Level × Exposure	–0.144	0.139	–1.035	139	*0.302*	[–0.859, 0.371]
** Sex × Exposure**	–0.306	0.143	–2.140	139	***0.034***	[–1.070, 0.205]
** Age × Exposure**	–0.343	0.146	–2.342	139	***0.021***	[–1.111, 0.148]
** Division × Level × Sex**	0.334	0.160	2.088	139	***0.039***	[–0.157, 1.377]
** **Division × Level × Age	0.236	0.148	1.598	139	*0.112*	[–0.173, 1.055]
** Division × Sex × Age**	0.377	0.180	2.094	139	***0.038***	[–0.047, 1.189]
** Level × Sex × Age**	0.484	0.175	2.757	139	***0.007***	[0.022, 0.831]
** **Division × Level × Exposure	0.059	0.173	0.339	139	*0.735*	[–0.391, 0.860]
** **Division × Sex × Exposure	0.007	0.176	0.043	139	*0.966*	[–0.540, 0.799]
** **Level × Sex × Exposure	0.198	0.183	1.078	139	*0.283*	[–0.490, 1.170]
** **Division × Age × Exposure	0.055	0.175	0.317	139	*0.752*	[–0.431, 0.858]
** **Level × Age × Exposure	0.017	0.172	0.102	139	*0.919*	[–0.728, 0.874]
** Sex × Age × Exposure**	0.482	0.199	2.418	139	***0.017***	[–0.085, 1.286]
** Division × Level × Sex × Age**	–0.503	0.220	–2.285	139	***0.024***	[–1.612, 0.093]
** **Division × Level × Sex × Exposure	–0.074	0.233	–0.320	139	*0.749*	[–1.108, 0.613]
** **Division × Level × Age × Exposure	0.089	0.223	0.397	139	*0.692*	[–0.928, 0.815]
** **Division × Sex × Age × Exposure	–0.163	0.244	–0.669	139	*0.505*	[–1.065, 0.430]
** **Level × Sex × Age × Exposure	–0.228	0.235	–0.967	139	*0.335*	[–1.236, 0.623]
** **Division × Level × Sex × Age × Exposure	0.064	0.309	0.209	139	*0.835*	[–0.707, 1.572]
**iPE**						
** ** (Intercept)	–0.008	0.031	–0.276	139	*0.783*	[–0.069, 0.052]
** **Division	0.049	0.048	1.031	139	*0.304*	[–0.045, 0.143]
** **Level	0.037	0.056	0.649	139	*0.518*	[–0.075, 0.185]
** Sex**	–0.120	0.038	–3.128	139	***0.002***	[–0.196, –0.044]
** **Age	–0.057	0.040	–1.448	139	*0.150*	[–0.136, 0.021]
** **Exposure	–0.065	0.043	–1.502	139	*0.135*	[–0.150, 0.021]
** **Division × Level	0.055	0.066	0.831	139	*0.407*	[–0.075, 0.185]
** **Division × Sex	0.003	0.063	0.050	139	*0.960*	[–0.122, 0.128]
** **Level × Sex	0.063	0.083	0.759	139	*0.449*	[–0.101, 0.226]
** **Division × Age	–0.101	0.068	–1.482	139	*0.141*	[–0.236, 0.034]
** **Level × Age	0.005	0.070	0.073	139	*0.942*	[–0.134, 0.145]
** Sex × Age**	0.138	0.055	2.499	139	***0.014***	[0.029, 0.247]
** **Division × Exposure	–0.084	0.070	–1.199	139	*0.233*	[–0.222, 0.055]
** Level × Exposure**	–0.171	0.086	–1.993	139	***0.048***	[–0.341, –0.001]
** **Sex × Exposure	0.055	0.053	1.037	139	*0.301*	[–0.049, 0.158]
** **Age × Exposure	0.029	0.054	0.531	139	*0.596*	[–0.079, 0.094]
** **Division × Level × Sex	–0.090	0.093	–0.966	139	*0.336*	[–0.275, 0.094]
** Division × Level × Age**	–0.294	0.091	–3.215	139	***0.002***	[–0.475, –0.113]
** **Division × Sex × Age	–0.020	0.097	–0.208	139	*0.835*	[–0.211, 0.171]
** **Level × Sex × Age	–0.133	0.105	–1.269	139	*0.207*	[–0.275, 0.094]
** Division × Level × Exposure**	0.219	0.099	2.221	139	***0.028***	[0.024, 0.414]
** **Division × Sex × Exposure	0.010	0.096	0.106	139	*0.915*	[–0.180, 0.201]
** **Level × Sex × Exposure	0.125	0.111	1.127	139	*0.262*	[–0.179, 0.200]
** **Division × Age × Exposure	0.007	0.095	0.070	139	*0.944*	[–0.337, 0.246]
** **Level × Age × Exposure	0.110	0.107	1.036	139	*0.302*	[–0.135, 0.321]
** **Sex × Age × Exposure	–0.053	0.074	–0.709	139	*0.479*	[–0.200, 0.094]
** **Division × Level × Sex × Age	0.374	0.130	2.870	139	*0.005*	[0.117, 0.632]
** **Division × Level × Sex × Exposure	0.023	0.138	0.170	139	*0.865*	[–0.249, 0.296]
** **Division × Level × Age × Exposure	–0.019	0.133	–0.141	139	*0.888*	[–0.283, 0.295]
** **Division × Sex × Age × Exposure	0.068	0.137	0.497	139	*0.620*	[–0.283, 0.387]
** **Level × Sex × Age × Exposure	–0.040	0.143	–0.278	139	*0.781*	[–0.596, 0.137]

The table presents *generalized linear mixed-effects model* results with the following abbreviations: Term refers to the fixed effect or interaction; *Estimate* denotes the estimated coefficient; *SE* is the standard error; *t-Stat* represents the *t*-statistic; *DF* stands for degrees of freedom; *p-value* indicates the probability of observing the result under the null hypothesis; and *95% CI [Lower, Upper]* shows the 95% confidence interval bounds. Statistically significant effects (*p* < 0.05) are highlighted in bold.

**Fig 7:**
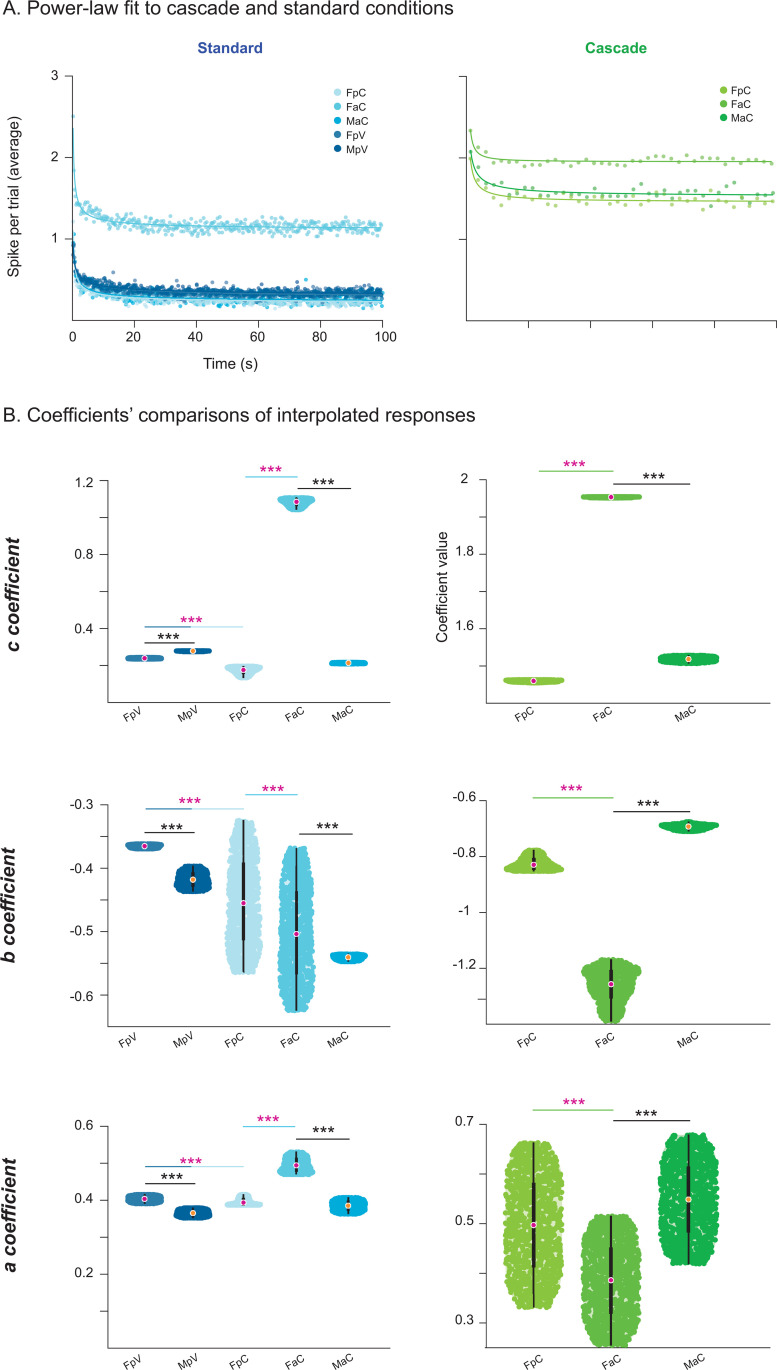
Time course of adaptation in cascade and standard conditions in control and VPA-exposed rats. **A.** Time course of long-term adaptation to CAS (green) and STD (blue) tones in the non-lemniscal IC under high stimulation level (≥40 dB SPL) in control and VPA-exposed rats. Solid lines represent power-law fits. **B.** Violin plots show the distribution of power-law parameters extracted from interpolated responses: *a* (initial response), *b* (long-term decay rate), and *c* (steady state). Vertical lines indicate statistically significant differences across sex, age, and exposure comparisons using Wilcoxon rank-sum tests with false discovery rate correction (*p < 0.05, **p = 0.001, ***p < 0.001), performed when comparisons were possible. Group abbreviations: FpC = female prepubertal control, FaC = female adult control, MaC = male adult control, FpV = female prepubertal VPA, MpV = male prepubertal VPA. General abbreviations: IC, inferior colliculus; VPA, valproic acid; CAS, cascade; STD, standard. See S7 Data.

**Fig 8:**
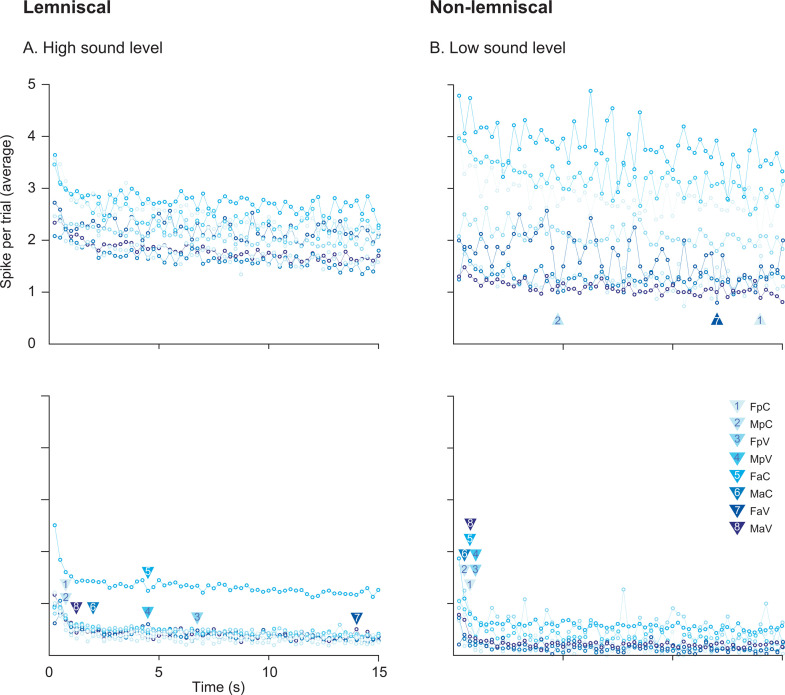
Half adaptation to standard stimuli in control and VPA-exposed rats. **A–B.** Time course of short-term adaptation to STD tones during the first 15 s of stimulation. Arrows indicate the estimated “half adaptation” point in response to STD conditions for control and VPA-exposed groups. **A** shows normalized responses at high sound levels (≥40 dB SPL), and **B** shows normalized responses at low sound levels (<40 dB SPL). Group abbreviations: FPC, female prepubertal control, FaC = female adult control, MpC = male prepubertal control, MaC = male adult control, FpV = female prepubertal VPA, FaV = female adult VPA, MpV = male prepubertal VPA, MaV = male adult VPA. See S8 Data.

To characterize adaptation dynamics, we applied two complementary approaches. First, we averaged spike counts across trials for DEV, CAS, and STD tones and fit a power-law model to capture long-term adaptation. We evaluated the goodness of fit and model coefficients (Table 5), using 1,000 interpolated iterations (*interp1* function in MATLAB) to estimate variability (Fig 7) [49]. Second, we calculated half-adaptation times of STD responses, defined as the time required for STD activity to decay to half of their initial value, to assess short-term adaptation (Fig 8). We evaluated sex, age, and prenatal exposure differences across divisions and sound intensities.

**Table 5:** Power-law fit parameters (*a, b, c*) and model fit quality (R^²^) for DEV, CAS, and STD conditions across experimental groups in the lemniscal and non-lemniscal inferior colliculus under high- and low-intensity sound stimulation.

	Control								VPA							
	Female				Male				Female				Male			
	Prepubertal		Adult		Prepubertal		Adult		Prepubertal		Adult		Prepubertal		Adult	
	IC_L_	IC_NL_	IC_L_	IC_NL_	IC_L_	IC_NL_	IC_L_	IC_NL_	IC_L_	IC_NL_	IC_L_	IC_NL_	IC_L_	IC_NL_	IC_L_	IC_NL_
**High-intensity sound stimulation (≥40 dB SPL)**																
DEV R^2^	0.5623	***0.7718***	0.5707	0.4997	0.1729	0.5107	0.1501	0.5765	0.0838	0.4524	0.0000	0.1579	0.2909	***0.6944***	0.5133	0.3510
DEV *a*	–	0.6011	–	–	–	–	–	–	–	–	–	–	–	0.4412	–	–
DEV *b*	–	−1.318	–	–	–	–	–	–	–	–	–	–	–	−1.121	–	–
DEV *c*	–	1.207	–	–	–	–	–	–	–	–	–	–	–	1.104	–	–
CAS R^2^	0.5834	***0.7107***	0.3092	***0.7174***	0.0934	0.6388	0.3845	***0.7239***	0.2015	0.5687	0.0199	0.3957	0.6050	***0.6702***	0.5483	0.5655
CAS *a*	–	0.6631	–	0.5158	–	–	–	0.6764	–	–	–	–	–	0.3852	–	–
CAS *b*	–	−0.8623	–	−1.178	–	–	–	−0.697	–	–	–	–	–	−0.8003	–	–
CAS *c*	–	1.456	–	1.95	–	–	–	1.511	–	–	–	–	–	1.269	–	–
STD R^2^	0.6442	***0.7677***	0.3802	***0.7543***	0.4390	0.5532	0.6374	***0.6726***	0.5244	***0.6726***	0.2161	0.5973	0.5521	***0.7622***	***0.7589***	0.5238
STD *a*	–	0.4144	–	0.5292	–	–	–	0.4055	–	0.4162	–	–	–	0.3794	0.9738	–
STD *b*	–	−0.5665	–	−0.6258	–	–	–	−0.5382	–	−0.3605	–	–	–	−0.4363	−0.2457	–
STD *c*	–	0.1969	–	1.108	–	–	–	0.2096	–	0.2339	–	–	–	0.2792	1.135	–
**Low-intensity sound stimulation (<40 dB SPL)**																
DEV R^2^	0.0950	0.1177	0.0000	***0.6989***	0.0612	0.2709	0.2026	0.2116	0.0442	0.0789	0.0010	0.2785	0.0797	0.4721	0.3138	0.5059
DEV *a*	–	–	–	0.6013	–	–	–	–	–	–	–	–	–	–	–	–
DEV *b*	–	–	–	−0.9225	–	–	–	–	–	–	–	–	–	–	–	–
DEV *c*	–	–	–	1.244	–	–	–	–	–	–	–	–	–	–	–	–
CAS R^2^	0.0048	0.1637	0.0939	***0.7879***	0.0341	0.2645	0.0409	0.3021	0.0000	0.0879	0.0000	0.0443	0.1206	0.5908	0.1301	0.5168
CAS *a*	–	–	–	0.745	–	–	–	–	–	–	–	–	–	–	–	–
CAS *b*	–	–	–	−0.8638	–	–	–	–	–	–	–	–	–	–	–	–
CAS *c*	–	–	–	1.082	–	–	–	–	–	–	–	–	–	–	–	–
STD *R*^2^	0.2552	0.1203	0.2414	***0.7035***	0.0690	0.1845	0.0660	0.3602	0.2852	0.0312	0.1538	0.1231	0.6050	0.3248	0.3626	0.3916
STD *a*	–	–	–	0.3655	–	–	–	–	–	–	–	–	–	–		
STD *b*	–	–	–	−0.9138	–	–	–	–	–	–	–	–	–	–		
STD *c*	–	–	–	0.4591	–	–	–	–	–	–	–	–	–	–		

*R*² values represent model fit quality for each condition (DEV, CAS, STD). Parameters *a* (initial response), *b* (decay rate), and *c* (steady state) were extracted from power-law fits applied to individual time courses. In bold, the high-quality *R*². Dashes (–) indicate that power-law parameters were not computed due to poor fit or lack of convergence, particularly in groups where adaptation was not reliably modeled (e.g., prepubertal VPA-exposed females).

### Spontaneous neuronal activity in the inferior colliculus

We fit a generalized linear mixed-effects model to examine how IC division, sex, age, and VPA exposure influenced spontaneous neuronal activity. The model included random intercepts and slopes for division and exposure within subjects. We specified a gamma distribution with a log link function to appropriately model the non-parametric structure of the data. The model yielded an intraclass correlation coefficient of 0.916 and a median absolute error of 0.880, indicating that it reliably captured between-subject variability in spontaneous activity (Table 2).

The model for spontaneous neuronal activity identified significant main effects of division (*p < *0.001), sex (*p < *0.001), age (*p < *0.001), and exposure (*p < *0.001). It also revealed several significant two-way interactions, including division × sex (*p < *0.001), division × age (*p < *0.001), division × exposure (*p < *0.001), sex × age (*p < *0.001), sex × exposure (*p = *0.001), and age × exposure (*p = *0.002). Significant three-way interactions emerged as well: division × sex × age (*p < *0.001), division × sex × exposure (*p = *0.004), division × age × exposure (*p = *0.004), and sex × age × exposure (*p = *0.005). Finally, the model detected a significant four-way interaction between division, sex, age, and exposure (*p = *0.009). These results (Table 2) indicate that both individual predictors and their higher-order combinations influence spontaneous firing in the inferior colliculus.

Because division significantly interacted with other fixed effects, we compared spontaneous activity within each division to assess the effects of sex, age, and exposure in the lemniscal and non-lemniscal IC (Fig 2A).

Within the lemniscal division, we detected significant effects of sex and age on spontaneous activity (Fig 2A). Females exhibited significantly greater spontaneous activity than males (*p* < 0.001, median difference = 1.178), and prepubertals showed larger spontaneous rate than adult animals (*p* < 0.001, median difference = 1.092). In the non-lemniscal division, significant effects emerged for sex (*p* = 0.004, median difference = 0.009), age (*p* = 0.001, median difference = 0.024), and exposure (*p* = 0.041, median difference = –0.125). As in the lemniscal division, females exhibited increased spontaneous rate than males, and prepubertals showed larger responses than adult individuals. Prenatal VPA exposure significantly augmented spontaneous activity in the non-lemniscal division compared to controls.

Overall, these findings indicate that sex, age, and exposure influence spontaneous activity differently across the two divisions of the IC. To examine these patterns in greater detail and isolate effects at the experimental group level, we compared spontaneous activity across experimental groups within each IC division (Fig 2B).

Experimental group-level analysis of spontaneous activity revealed distinct effects of sex, age, and exposure across IC divisions (Fig 2B). In the lemniscal division, adult females exhibited significantly greater spontaneous firing than males in both the control (*p = *0.008, median difference = 0.630) and VPA groups (*p < *0.001, median difference = 2.828). In contrast, in the non-lemniscal division, VPA-exposed adult and prepubertal females displayed significantly smaller spontaneous activity compared to males (both *p < *0.001, median differences = –0.725 and –0.154, respectively).

Developmental effects were more selective (Fig 2B). Among males in the lemniscal division, control prepubertal rats showed reduced spontaneous firing relative to adults (*p = *0.041, median difference = –0.034), whereas VPA-exposed prepubertal males exhibited increased activity compared to adults (*p < *0.001, median difference = 1.707). In the non-lemniscal division, males demonstrated an age-related decrease in spontaneous firing across control (*p < *0.001, median difference = 0.203), while VPA-exposed males exhibited a developmental increase (*p = *0.003, median difference = –0.547). Among females, only control animals showed a significant developmental augmentation in the non-lemniscal division (*p = *0.040, median difference = –0.027).

Prenatal VPA exposure further modulated spontaneous activity in a division- and group-specific manner (Fig 2B). In the lemniscal division, VPA exposure significantly augmented spontaneous activity in adult females (*p = *0.045, median difference = –2.019) and prepubertal males (*p = *0.002, median difference = –1.563). In the non-lemniscal division, prenatal VPA administration increased spontaneous activity in adult males (*p < *0.001, median difference = –0.915), but reduced it in prepubertal females (*p = *0.001, median difference = 0.091).

Collectively, these findings indicate that sex, age, and prenatal exposure differentially modulate spontaneous activity in the IC. While significant effects emerged across both divisions, the non-lemniscal region demonstrated markedly greater variability in spontaneous activity patterns.

### Deviant-, cascade-, and standard-evoked responses

We fit a generalized linear mixed-effects models to examine how division, sound level, sex, age, and exposure influenced the DEV-, CAS-, and STD-evoked responses. Each model included random intercepts and slopes for division, level, and exposure within subjects. We selected suitable distributions and link functions for each response variable: a gamma distribution with a log link for DEV- and CAS-elicited activity, and a normal distribution with an identity link for STD-evoked responses.

All models demonstrated good fit. The intraclass correlation coefficients were 0.916 for DEV, 0.951 for CAS, and 0.737 for STD conditions. Corresponding median absolute errors were 0.064, 0.054, and 0.074, respectively, indicating that the models yielded accurate predictions and effectively captured between-subject variability (Table 3).

The DEV-response model identified significant main effects of division (*p < *0.001), sound level (*p < *0.001), sex (*p = *0.011), age (*p = *0.011), and exposure (*p = *0.007). It also revealed several significant two-way interactions: division × sex (*p = *0.015), level × sex (*p = *0.045), division × age (*p = *0.005), division × exposure (*p = *0.014), level × exposure (*p = *0.003), sex × exposure (*p = *0.016), and age × exposure (*p = *0.016). These results, together with the significant three- and five-way interactions (Table 3), indicate that both the main factors and their interactions influenced DEV-evoked responses.

For CAS-evoked responses, we observed significant main effects of division (*p = *0.003), level (*p = *0.001), sex (*p = *0.001), age (*p = *0.007), and exposure (*p < *0.001). In addition, the model identified significant interactions between division × level (*p = *0.002), division × sex (*p = *0.040), level × sex (*p = *0.005), level × age (*p = *0.031), sex × age (*p = *0.003), sex × exposure (*p = *0.008), and age × exposure (*p = *0.014). These patterns, together with the significant three-, four-, and five-way interactions (Table 3), highlight the combined influence of the experimental variables on CAS-elicited responses.

The model for STD-evoked activity revealed significant main effects of division (*p < *0.001), level (*p = *0.003), sex (*p = *0.001), age (*p = *0.006), and exposure (*p = *0.014). Among interaction terms, sex × age (*p = *0.001), age × exposure (*p* = 0.036), and sex × age × exposure (*p* = 0.014) reached significance (Table 3).

Higher-order interactions significantly affected all evoked brain responses, particularly for the DEV and CAS conditions (Table 3), reflecting the complex influence of sex, age, and exposure across IC divisions and sound levels. We assessed these effects for DEV-, CAS-, and STD-evoked responses within each auditory division and sound level (Fig 3).

Within the lemniscal division, none of the comparisons for sex, age, or exposure reached significance for DEV-evoked activity. In contrast, the non-lemniscal division exhibited robust effects (Fig 3). At the high stimulation level, prepubertal animals showed significantly reduced DEV-elicited responses compared to adults (*p < *0.001, median difference = –0.021), and control rats demonstrated increased scores than their VPA-exposed counterparts (*p < *0.001, median difference = 0.021). Age-related differences remained significant at the low stimulation level, with prepubertal individuals again exhibiting markedly diminished responses (*p < *0.001, median difference = –0.100).

For CAS-evoked responses in the lemniscal division (Fig 3), males displayed significantly greater activity than females under high stimulation (*p < *0.001, median difference = –0.024). In the non-lemniscal division, all three factors (sex, age, and exposure) significantly influenced CAS responses. At the high level, males outperformed females (*p < *0.001, median difference = –0.024), prepubertal animals exhibited greater activity than adults (*p < *0.001, median difference = 0.048), and VPA-exposed subjects exceeded control animals (*p < *0.001, median difference = –0.029). These differences became even more pronounced at the low stimulation level, with significant effects observed for age (*p < *0.001, median difference = 0.108), sex (*p < *0.001, median difference = –0.051), and exposure (*p = *0.005, median difference = –0.039).

STD-evoked responses also varied across divisions and stimulation levels (Fig 3). In the lemniscal division, females exhibited significantly stronger responses than males at both high (*p < *0.001, median difference = 0.053) and low (*p = *0.029, median difference = 0.038) stimulation levels. In the non-lemniscal division, both sex (*p < *0.001, median difference = 0.033) and age (*p < *0.001, median difference = –0.047) significantly modulated STD responses at high intensity, with females and adults showing greater activity than males and prepubertal animals, respectively. At the low stimulation level, only sex differences remained significant (*p = *0.029, median difference = 0.020).

Together, these results show that sex, age, and exposure strongly influence DEV-, CAS-, and STD-evoked responses, particularly in the non-lemniscal division. We further examined these effects across experimental groups within IC divisions and stimulation levels (Fig 4).

Experimental group-level analysis of DEV-elicited activity revealed significant sex effects, with patterns varying across IC divisions and sound levels (Fig 4). Under high-level stimulation, control adult females exhibited reduced DEV responses compared to males in the lemniscal division (*p* = 0.016, median difference = –0.045), but larger responses in the non-lemniscal division (*p* = 0.048, median difference = 0.022). Among prepubertal animals exposed to VPA, females showed smaller DEV activity than males only in the non-lemniscal division under high-level stimulation (*p* = 0.003, median difference = –0.034).

Developmental differences robustly emerged in the non-lemniscal regions across groups, with adults generally showing stronger DEV-elicited responses than prepubertal animals (Fig 4). In the control group, adult females exhibited a significant decrease in DEV responses compared to prepubertal females in the lemniscal division at high-level stimulation (*p* = 0.001, median difference = 0.049). In contrast, DEV responses increased with age in the non-lemniscal division at both high (*p* = 0.035, median difference = –0.014) and low (*p* = 0.001, median difference = –0.175) stimulation levels. Adult control males also showed stronger responses than prepubertal males in the non-lemniscal division at low stimulation (*p* < 0.001, median difference = –0.114). Among VPA-exposed animals, females exhibited age-related increases in DEV responses in the non-lemniscal division at both high (*p* = 0.001, median difference = –0.055) and low levels (*p* < 0.001, median difference = –0.147). VPA-exposed males also showed increased DEV responses with age in the non-lemniscal division at low stimulation (*p* < 0.001, median difference = –0.069).

Prenatal VPA exposure significantly influenced DEV-elicited responses in female rats (Fig 4). Among adult females, VPA exposure increased DEV responses compared to controls in the lemniscal division at high-level stimulation (*p* = 0.043, median difference = –0.051). In contrast, prepubertal females exhibited increased DEV responses following VPA exposure in the non-lemniscal division at high-level stimulation (*p* = 0.001, median difference = 0.043).

Significant sex differences in CAS-evoked responses appeared in both divisions and varied by age and exposure (Fig 4). In the lemniscal division, adult VPA-exposed females exhibited smaller CAS responses than males at high-level stimulation (corrected *p* = 0.002, median difference = –0.044). In the non-lemniscal division, control adult females also showed reduced responses than males at high stimulation (*p* < 0.001, median difference = –0.107). Conversely, among prepubertal VPA-exposed animals, females exhibited greater CAS-elicited activity than males at high-level stimulation in the non-lemniscal division (*p* = 0.002, median difference = 0.039).

Age comparisons revealed significant developmental decreases in CAS-evoked responses, particularly in the non-lemniscal division (Fig 4). In the lemniscal division, control females exhibited greater responses in prepubertal animals than adults at low-level stimulation (*p* = 0.039, median difference = 0.048). In the non-lemniscal division, both control and VPA-exposed females showed similar patterns. Control females had stronger CAS responses in prepubertals at both high (*p* < 0.001, median difference = 0.096) and low (*p* < 0.001, median difference = 0.197) stimulation levels, and VPA-exposed females showed greater prepubertal responses at both high (*p* < 0.001, median difference = 0.049) and low (*p* < 0.001, median difference = 0.142) levels. Among males, developmental decreases also emerged. Control males showed higher responses in prepubertals than adults at low-level stimulation (*p* < 0.001, median difference = 0.121), and VPA-exposed males showed a similar decrease (*p* < 0.001, median difference = 0.062) in the non-lemniscal IC.

Prenatal VPA exposure significantly influenced CAS-elicited responses in the non-lemniscal IC of female rats (Fig 4). Among adult females at high-level stimulation, VPA exposure increased CAS-control responses compared to controls (*p* < 0.001, median difference = –0.074). A similar increase appeared in prepubertal females at high-level stimulation (*p* = 0.032, median difference = –0.027).

Experimental group-level comparisons revealed significant sex differences in STD-elicited responses, particularly among adults (Fig 4). In the lemniscal division, adult VPA-exposed females exhibited stronger STD responses than males at high-level stimulation (*p* < 0.001, median difference = 0.087). Control adult females also showed larger responses than males at low-level stimulation (*p* = 0.005, median difference = 0.090). In the non-lemniscal division, control adult females showed stronger STD responses than males at both high (*p* < 0.001, median difference = 0.194) and low stimulation levels (*p* = 0.008, median difference = 0.059).

Significant developmental effects emerged among control female animals (Fig 4). In the lemniscal division, females increased STD responses with age at low-level stimulation (*p* = 0.004, median difference = –0.113). In the non-lemniscal division, adult females showed stronger responses than prepubertal females at high-level stimulation (*p* < 0.001, median difference = –0.180).

Prenatal VPA exposure significantly altered STD-elicited responses across groups (Fig 4). In the lemniscal division, VPA exposure increased STD responses in prepubertal females at high-level stimulation (*p* = 0.017, median difference = –0.068), while it reduced responses in adult females at low-level stimulation (*p* = 0.037, median difference = 0.107). In the non-lemniscal division, VPA increased STD responses in prepubertal females at high-level stimulation (*p* = 0.004, median difference = –0.033) but reduced responses in adult females at high-level stimulation (*p* < 0.001, median difference = 0.138). Among males, VPA consistently increased STD responses. This increase appeared in adults at high-level stimulation (*p* < 0.001, median difference = –0.064) and in prepubertal males at both high (*p* < 0.001, median difference = –0.044) and low stimulation levels (*p* = 0.051, median difference = –0.041).

Our findings demonstrate that sex, age, and prenatal VPA exposure significantly modulated DEV-, CAS-, and STD-elicited responses, with distinct patterns, particularly in the non-lemniscal IC. Control females consistently exhibited stronger STD and DEV responses, but reduced CAS activity compared to males in adult animals. Developmental effects were also evident, with DEV and STD responses increasing with age, while CAS responses declined, especially within non-lemniscal regions. Prenatal VPA exposure exerted sex- and age-dependent influences, introducing sex differences among prepubertals and altering the maturation of contextual auditory processing. Notably, VPA primarily affected elicited DEV and CAS responses in females compared to their control counterparts, and STD activity across sexes and developmental stages.

### Predictive components of mismatch processing

We fit a generalized linear mixed-effects models to examine how IC division, sound level, sex, age, and prenatal exposure influenced the predictive components of mismatch processing—indexed by iMM, iRS, and iPE. Each model included random intercepts and slopes for division, level, and exposure within subjects. We used a normal distribution with an identity link for all three indices.

The models demonstrated strong performance for iMM and iRS, with intraclass correlation coefficients of 0.856 and 0.681, and median absolute errors of 0.112 and 0.089, respectively. In contrast, the iPE model yielded a low intraclass correlation coefficient of 0.040, which aligns with theoretical expectations. Subcortical regions like the inferior colliculus generate relatively small prediction error signals, especially at the lemniscal level [41], and likely exhibit minimal inter-individual variability explained by experimental variables. Our modeling supports this view. Despite the low intraclass correlation, the model showed good fit (Akaike Information Criterion = –286.792; Bayesian Information Criterion = –151.700) and acceptable predictive accuracy (median absolute error of 0.070), indicating it reliably captured systematic effects of auditory division, sound level, sex, age, and prenatal exposure for iPE.

The iMM model revealed significant main effects of division (*p < *0.001), level (*p < *0.001), sex (*p = *0.001), age (*p = *0.002), and exposure (*p = *0.003). It also captured several significant two-way interactions, including sex × age (*p < *0.001), level × exposure (*p = *0.005), sex × exposure (*p = *0.013), and age × exposure (*p = *0.005). These results, together with the significant three-way interactions reported in Table 4, indicate that both individual factors and their interactions contribute to modulating neuronal mismatch.

For iRS, the model detected significant main effects of division (*p < *0.001), sound level (*p < *0.001), sex (*p < *0.001), age (*p < *0.001), and prenatal exposure (*p = *0.004). It also revealed significant two-way interactions of division × level (*p = *0.001), level × sex (*p = *0.011), sex × age (*p < *0.001), sex × exposure (*p* = 0.034), and age × exposure (*p = *0.021). These patterns, together with the significant three- and four-way interactions (Table 4), indicate that experimental factors and their interactions jointly influence repetition suppression.

The iPE model identified significant main effects of sex (*p = *0.002) and a significant sex × age interaction (*p = *0.014), along with a marginally significant level × exposure interaction (*p = *0.048), division × level × age (*p* = 0.002) and division × level × exposure (*p* = 0.028). Other predictors did not reach significance, indicating that prediction error signals were more selectively modulated (Table 4).

Higher-order interactions significantly modulated all predictive components, particularly iMM and iRS (Table 4), highlighting the complex influence of sex, age, and prenatal exposure across IC divisions and stimulation levels. We further examined these effects for each index by sound level and IC division (Fig 5).

The analyses of the iMM revealed marked differences across auditory divisions and stimulation levels (Fig 5). In the lemniscal division, sex differences emerged prominently at the high stimulation level, with males exhibiting significantly greater mismatch indices than females (*p < *0.001, median difference = –0.074). The non-lemniscal division showed broader effects: at high stimulation, both sex (*p = *0.003, median difference = –0.036) and exposure (*p = *0.019, median difference = 0.022) significantly influenced iMM, with males and control animals displaying greater values than females and VPA-exposed rats, respectively. At low stimulation, age was the predominant factor, as prepubertal animals displayed significantly smaller iMM compared to adults (*p < *0.001, median difference = –0.119).

The iRS exhibited a comparable pattern of group differences (Fig 5). In the lemniscal division, males showed significantly increased iRS scores than females at both high (*p < *0.001, median difference = –0.077) and low (*p = *0.044, median difference = –0.046) stimulation levels. At the low level, VPA exposure also significantly modulated iRS (*p = *0.043, median difference = –0.066), with VPA-exposed animals showing elevated scores relative to controls. In the non-lemniscal division, sex, age, and exposure each significantly influenced iRS at both stimulation levels. Under high stimulation, males again outperformed females (*p < *0.001, median difference = –0.045), prepubertal animals showed stronger iRS than adults (*p < *0.001, median difference = 0.096), and VPA-exposed animals displayed greater iRS than controls (corrected *p = *0.007, median difference = –0.021). These effects were amplified at low sound intensity, with males, prepubertal animals, and VPA-exposed groups continuing to exhibit significantly elevated iRS values (sex: *p < *0.001, median difference = –0.108; age: *p < *0.001, median difference = 0.136; exposure: *p = *0.008, median difference = –0.055).

The iPE exhibited a distinct pattern of effects (Fig 5). In the lemniscal division, none of the comparisons reached significance at either stimulation level. In contrast, the non-lemniscal division revealed clear and consistent differences. At the high stimulation level, sex (corrected *p = *0.011, median difference = 0.045), age (*p < *0.001, median difference = –0.081), and exposure (*p < *0.001, median difference = 0.056), each significantly modulated iPE, with females, adults, and control animals showing larger scores than males, prepubertals, and VPA-exposed rats, respectively. These effects persisted at the low stimulation level, where sex (corrected *p = *0.002, median difference = 0.065) and age (*p < *0.001, median difference = –0.191) continued to significantly influence iPE. Notably, iPE scores in adult animals became positive, whereas iPE values in prepubertal rats remained negative.

Our results demonstrate that sex, age, and exposure substantially influence the predictive components of mismatch processing, with the strongest effects observed in the non-lemniscal division. We further characterized these patterns at the experimental group level across auditory divisions and stimulation levels (Fig 6).

Experimental group-level analysis of the neuronal mismatch index revealed significant effects of sex, age, and exposure, with patterns that varied across IC divisions and stimulation levels (Fig 6). For sex differences, control adult females showed significantly smaller iMM than males in both the lemniscal (*p < *0.001, median difference = –0.092) and non-lemniscal divisions (*p < *0.001, median difference = –0.177) at the high stimulation level. This pattern persisted in the VPA group, where adult females exhibited reduced iMM compared to males in the lemniscal division (*p = *0.010, median difference = –0.061). Among prepubertal VPA-exposed animals, females also showed reduced indices than males in both the lemniscal (*p = *0.012, median difference = –0.079) and non-lemniscal divisions (*p = *0.029, median difference = –0.062) under high stimulation. At the low sound level, control adult females again demonstrated reduced iMM index compared to males in the lemniscal division (*p = *0.013, median difference = –0.090).

Age comparisons revealed robust developmental effects that varied by sex and exposure (Fig 6). In the lemniscal division, control prepubertal females exhibited significantly greater iMM than adult females at both high (*p < *0.001, median difference = 0.125) and low (*p = *0.020, median difference = 0.119) stimulation levels. In the non-lemniscal division, control prepubertal females also showed an elevated score relative to adults under high stimulation (*p < *0.001, median difference = 0.182). However, among VPA-exposed females, the trend reversed: prepubertal animals showed significantly attenuated iMM than adults at the same sound level (*p = *0.004, median difference = –0.106). At low stimulation in the non-lemniscal division, VPA-exposed prepubertal females also exhibited significantly smaller iMM than adult females (*p = *0.008, median difference = –0.146). Among males, prepubertal rats in the control group displayed a decreased iMM compared to adults in the non-lemniscal division at low stimulation (*p = *0.008, median difference = –0.118), a pattern that was also evident across VPA-exposed males (*p < *0.001, median difference = –0.118).

Prenatal VPA exposure produced divergent effects depending on age and IC division (Fig 6). In adult females, VPA exposure significantly increased iMM compared to controls in the non-lemniscal division under high stimulation (*p = *0.002, median difference = –0.153). Distinctly, among prepubertal females, prenatal VPA administration decreased iMM in both the lemniscal (*p = *0.002, median difference = 0.104) and non-lemniscal divisions (*p < *0.001, median difference = 0.136) at high sound level. Similarly, among males, VPA-exposed animals exhibited reduced iMM in prepuberty (*p = *0.004, median difference = 0.058) and adulthood (*p = *0.014, median difference = 0.054) at the same region and sound level.

Experimental group-level analysis of the repetition suppression index revealed robust sex differences (Fig 6). Under high-level stimulation in the lemniscal division, control adult females exhibited significantly smaller iRS than males (*p = *0.027, median difference = –0.133), as did adult females in the non-lemniscal division (*p < *0.001, median difference = –0.282). VPA-exposed adults maintained reduced lemniscal iRS relative to males (*p < *0.001, median difference = –0.130). At low-level stimulation, control adult females again showed smaller lemniscal iRS than males (*p = *0.007, median difference = –0.121) and reduced non-lemniscal iRS (*p = *0.002, median difference = –0.111). Among VPA-exposed prepubertals, females displayed greater non-lemniscal iRS than males at high stimulation (*p = *0.016, median difference = 0.044).

Age comparisons revealed divergent developmental trajectories of iRS. In the lemniscal division, control prepubertal females outperformed adults at both high (*p = *0.027, median difference = 0.124) and low (*p = *0.003, median difference = 0.167) levels, whereas VPA-exposed prepubertal males showed reduced iRS relative to VPA adults at high stimulation (*p = *0.026, median difference = –0.117). In the non-lemniscal division, control prepubertal females exhibited elevated iRS at both high (*p < *0.001, median difference = 0.282) and low (*p < *0.001, median difference = 0.286) levels. VPA-exposed prepubertal females mirrored these increases (high: *p = *0.028, median difference = 0.032; low: *p = *0.015, median difference = 0.121). Both control (*p = *0.001, median difference = 0.121) and VPA (*p = *0.007, median difference = 0.065) prepubertal males exceeded adults under low non-lemniscal stimulation.

Prenatal VPA exposure produced division- and group-specific effects. In the non-lemniscal division at high stimulation, VPA augmented adult female iRS (*p < *0.001, median difference = –0.228) but reduced adult male iRS (*p = *0.002, median difference = 0.076). By contrast, among prepubertals, control females exceeded VPA-exposed females in the lemniscal division at high stimulation (*p = *0.025, median difference = 0.059), and control males outperformed VPA males in the non-lemniscal division at both high (*p = *0.022, median difference = 0.030) and low (*p = *0.042, median difference = 0.041) levels. Finally, at low lemniscal stimulation, VPA-exposed adults, females (*p = *0.019, median difference = –0.173) and males (*p = *0.022, median difference = –0.121), displayed greater iRS than controls.

Experimental group-level analysis revealed significant differences in the prediction error index, with effects varying across IC divisions and stimulation levels (Fig 6). In the lemniscal division at high sound level, adult females exposed to VPA showed significantly greater iPE scores than males (*p = *0.042, median difference = 0.058). In contrast, the non-lemniscal division showed more pronounced sex effects. Under high stimulation, control adult females exhibited significantly greater iPE than males (*p < *0.001, median difference = 0.133). In the VPA group, prepubertal females had significantly smaller scores than males (*p < *0.001, median difference = –0.072).

We observed robust developmental influences on iPE in the non-lemniscal division (Fig 6). Among control females, prepubertal rats exhibited significantly smaller iPE compared to adults at both high (*p < *0.001, median difference = –0.120) and low (*p < *0.001, median difference = –0.356) stimulation. VPA-exposed females showed a similar pattern, with reduced scores in prepubertal animals at high (*p < *0.001, median difference = –0.119) and low (*p < *0.001, median difference = –0.284) levels. In males, control prepubertal rats showed a significant reduction in iPE at low stimulation (*p < *0.001, median difference = –0.224), and VPA-exposed prepubertals also demonstrated reduced iPE (*p < *0.001, median difference = –0.144) compared to their adult counterparts.

Prenatal VPA exposure significantly modulated prediction error processing, particularly in the non-lemniscal division of female rats under high stimulation. Adult control females displayed greater iPE values than their VPA-exposed counterparts (*p < *0.001, median difference = 0.070), and the same pattern emerged among prepubertal females (*p = *0.003, median difference = 0.069).

Sex, age, and prenatal VPA exposure distinctly modulated predictive coding indices across IC divisions and sound levels. Adult females consistently exhibited smaller iMM and iRS than males, especially in non-lemniscal regions, a pattern maintained in VPA-exposed animals. Developmentally, control prepubertal females showed elevated iMM and iRS, while VPA reversed these trajectories. iPE increased after puberty in controls but was reduced by VPA across ages, introducing prepubertal sex differences. Despite these disruptions, the typical shift from negative to positive iPE in non-lemniscal circuits remained preserved. Sex and age influenced predictive coding, and prenatal VPA exposure particularly disrupted these processes, especially in non-lemniscal regions of the inferior colliculus.

### Does spontaneous activity correlate with deviant processing?

Finally, we assessed whether spontaneous activity correlated with the DEV [49]. In control adult females, we observed positive correlations in the lemniscal division at high intensity (*ρ* = 0.573, *p < *0.001) and in the non-lemniscal region at high (*ρ* = 0.127, *p = *0.029) and low (*ρ* = 0.340, *p < *0.001) levels. Control adult males also showed positive associations in the lemniscal division at low intensity (*ρ* = 0.671, *p < *0.001) and in the non-lemniscal at high (*ρ* = 0.253, *p = *0.002) and low (*ρ* = 0.481, *p < *0.001) levels. In control prepubertal females, we detected positive correlations in the lemniscal division at low intensity (*ρ* = 0.284, *p = *0.030) and in the non-lemniscal region at low intensity (*ρ* = 0.709, *p = *0.010), but, intriguingly, a negative correlation in the lemniscal division at high intensity (*ρ* = –0.301, *p = *0.014). Control prepubertal males exhibited a positive correlation in the non-lemniscal region at high intensity (*ρ* = 0.238, *p = *0.006).

Among VPA-exposed animals, VPA adult females showed positive correlations in the non-lemniscal at high (*ρ* = 0.407, *p < *0.001) and low (*ρ* = 0.574, *p = *0.002) levels; VPA adult males exhibited positive associations in the lemniscal at high intensity (*ρ* = 0.469, *p < *0.001) and in the non-lemniscal division at high (*ρ* = 0.346, *p = *0.002) and low (*ρ* = 0.297, *p = *0.002) levels; and VPA prepubertal males displayed robust positive correlations in the lemniscal region at both high (*ρ* = 0.347, *p < *0.001) and low (*ρ* = 0.517, *p < *0.001) levels.

### Time course of adaptation in the inferior colliculus

To investigate the time course of long-term adaptation to DEV-, control-, and STD-evoked responses, we fit a power-law model (*y*(*t*) = a tᵇ + c; see “Materials and methods”) to the averaged spike counts, considering fits with *r*² ≥ 0.65 as high-quality [50]. Table 5 summarizes the goodness of fit, and coefficient estimates for these responses.

Control rats predominantly exhibited power-law dynamics in the non-lemniscal inferior colliculus under high stimulation levels. Prepubertal females showed power-law adaptation across all response types, while adult females and males displayed this pattern for CAS- and STD-evoked activity. Among VPA-exposed animals, adult males exhibited power-law fits for STD responses in the lemniscal division, whereas prepubertal females (STD responses) and prepubertal males (all responses) demonstrated power-law behavior in the non-lemniscal division. At low intensity, only control adult females in the non-lemniscal division exhibited power-law dynamics across all response types.

These findings suggest that prenatal VPA modifies how the inferior colliculus encodes stimulus statistics [51]. In other words, the presence or absence of power-law dynamics likely reflects disruptions in how neurons integrate repeated stimuli, potentially indicating atypical adaptation mechanisms or altered sensitivity to the temporal structure of sounds. These processes are essential for prediction and flexible auditory processing.

Due to inadequate power law fits in some experimental groups, we restricted our evaluation of adaptation coefficients to groups with well-fitting data. We interpolated non-lemniscal CAS- and STD-evoked responses at high intensity, generating 1,000 iterations per fit. These produced distributions for the *a* (initial response), *b* (long-term adaptation velocity), and *c* (steady-state response) coefficients (Table 6). We assessed sex, age, and exposure effects using Wilcoxon rank-sum tests with false discovery rate correction when comparisons were possible (Fig 7).

**Table 6:** Statistical significance of sex, age, and exposure comparisons for power-law coefficients (*a, b, c*) across experimental groups in the non-lemniscal inferior colliculus at high sound levels.

	CAS			STD		
Non-lemniscal division under high level stimulation						
	*a*	*b*	*c*	*a*	*b*	*c*
Adult Female Control	0.3859	−1.2574	1.9532	0.4938	−0.5039	1.0843
Adult Male Control	0.5485	−0.6928	1.5182	0.3850	−0.5407	0.2127
*p-value*	***0.0000***	***0.0000***	***0.0000***	***0.0000***	***0.0000***	***0.0000***
Prepubertal Female VPA	–	–	–	0.4031	−0.3653	0.2390
Prepubertal Male VPA	0.3097	−0.8497	1.2759	0.3651	−0.4184	0.2772
*p-value*	–	–	–	***0.0000***	***0.0000***	***0.0000***
Prepubertal Female Control	0.4969	−0.8294	1.4596	0.3937	−0.4551	0.1756
Adult Female Control	0.3859	−1.2574	1.9532	0.4938	−0.5039	1.0843
*p-value*	***0.0000***	***0.0000***	***0.0000***	***0.0000***	***0.0000***	***0.0000***
Prepubertal Female Control	0.4969	−0.8294	1.4596	0.3937	−0.4551	0.1756
Prepubertal Female VPA	–	–	–	0.4031	−0.3653	0.2390
*P-value*	–	–	–	***0.0000***	***0.0000***	***0.0000***

*P*-values are from Wilcoxon rank-sum tests with false discovery rate correction. Dashes (–) indicate that CAS or STD parameters were not available for prepubertal VPA-exposed females due to not fitting power-law modelling and thus, not undergoing iteration. Group comparisons are arranged in row pairs, with *p*-values corresponding to the row directly below.

In terms of sex, control adult males showed reduced initial and steady-state STD responses and faster decay compared to females. For CAS responses, males exhibited larger initial responses but slower adaptation. Among VPA rats, prepubertal males showed smaller initial STD responses, faster adaptation, and higher steady-state activity than females (all *p < *0.001; Fig 7B). Age comparisons revealed that control prepubertal females had lower initial and steady-state STD responses and slower decay than adults. CAS responses in prepubertal females showed greater initial responses but slower decay and lower steady-state activity compared to adults (all *p < *0.001; Fig 7B). Exposure effects showed that VPA prepubertal females had larger initial STD responses, higher steady-state values, and slower adaptation than controls (all *p < *0.001; Fig 7B).

In summary, control adult males exhibited faster long-term adaptation to STD tones than females. Adaptation velocity increased from prepuberty to adulthood in control females. Prenatal VPA exposure altered adaptation dynamics in prepubertal males and disrupted long-term adaptation in females by slowing decay and modifying steady-state responses.

### Half adaptation to standard conditions in the inferior colliculus

To assess early adaptation, we measured half adaptation time, defined as the trial at which STD-evoked responses decayed to half their initial value, and visually inspected group differences across divisions and sound levels.

For sex differences, at high intensity, control adult males adapted faster than females in the non-lemniscal division (Fig 8A). At low intensity, control prepubertal males adapted faster than females in the lemniscal division (Fig 8B). Among VPA-exposed rats, males consistently adapted faster than females in the non-lemniscal division, while VPA adult females preserved half adaptation in the lemniscal division at low intensity.

Age comparisons showed that half adaptation slowed with development in controls, particularly among adult females in the non-lemniscal division (Fig 8A). In the lemniscal division, prepubertal rats exhibited half adaptation at low intensity, which vanished in adulthood. In VPA rats, adult males adapted faster than prepubertals, while adult females showed slower adaptation in the non-lemniscal division but retained adaptation at low intensity in the lemniscal division.

Prenatal VPA exposure delayed half adaptation in prepubertal males and females at high intensity in the non-lemniscal division. In adulthood, VPA-exposed males adapted faster than controls, whereas females adapted more slowly. At low intensity, VPA exposure abolished half adaptation in prepubertal rats in the lemniscal division but preserved it in adult females.

## Discussion

This study examined how sex, age, and prenatal VPA exposure influence contextual sound processing at the subcortical level in rats. We demonstrate that prenatal VPA exposure profoundly alters both spontaneous firing and predictive processing of context-evoked cues in the IC. Our findings also reveal sex- and age-related differences at the single-neuron level in both control and VPA-exposed rats. Collectively with previous research [14,34,38,52], our results have important implications for understanding the neural mechanisms underlying atypical predictive processing in VPA-exposed rats and may offer translational insights into the heterogeneity of autism symptoms.

### Spontaneous activity and mismatch signaling

To our knowledge, this is the first study to assess spontaneous activity at the single-neuron level while considering sex, age, and prenatal VPA exposure. Females exhibited greater spontaneous activity than males (Fig 2), consistent with resting-state functional imaging studies reporting stronger within-cortical and subcortical processing in female rats [53]. Spontaneous activity decreased with age throughout IC divisions (Fig 2). Prior single-unit recordings showed comparable age-related declines in spontaneous firing in the medial orbitofrontal cortex [54]. In predictive coding terms, this reduction likely reflects the refinement of internal models as they align with the statistical regularities of auditory scenes accumulated through postnatal experience [55, 56].

VPA-exposed rats showed increased spontaneous activity in the non-lemniscal division compared to controls (Fig 2). Group-level analyses revealed that prenatal VPA disrupted spontaneous activity in a sex- and age-specific manner across divisions (Fig 2). Spontaneous activity before hearing onset is essential for shaping the auditory system, propagating through the auditory hierarchy and guiding the formation of neural connections [57–60]. Disruptions during this critical developmental window, including those caused by prenatal VPA exposure, can alter both evoked and spontaneous neural activity throughout the central auditory pathway, including the IC [60,61].

While single-neuron data are scarce in humans, resting-state neuroimaging studies consistently report atypical spontaneous brain activity in autistic individuals, often with sex-dependent differences in cortical networks [62,63]. Developmental studies also reveal divergent trajectories of spontaneous cortical activity in autism [64], although sex-specific patterns remain underexplored. In the Bdnf Pax2 knockout mouse model of autism, impaired maturation of the high-spontaneous rate auditory nerve fibers alter spike synchrony and affect cortical auditory processing [65,66]. At the subcortical level, studies considering sex differences remain scarce, but a recent investigation in autistic individuals reported reduced activation in the inferior colliculus during voice processing, which may impact social reciprocity differently across sexes [14].

Spontaneous activity is thought to refine internal models through cortical-subcortical interactions in the absence of external stimulation [55]. Spearman correlations showed that spontaneous activity predicted mismatch signaling in a sex-, age-, and exposure-specific manner. VPA-exposed prepubertal females showed no significant correlations between spontaneous activity and iMM, suggesting a developmental disruption.

These findings indicate that prenatal VPA alters the relationship between spontaneous activity and mismatch signaling in a sex- and age-specific manner, highlighting developmental windows of sensitivity in females. Female-specific vulnerability is further supported by findings in mice, where social isolation selectively reduced serotonergic fibers in females but not males [67]. Pubertal hormone effects may also contribute, as estrogen receptor alpha is present in non-lemniscal regions of the IC and modulates auditory processing [68]. Reductions in estrogen levels previously reported in female rats chronically exposed to VPA [69] may influence spontaneous activity and its relationship to auditory mismatch signaling during critical developmental periods.

### Auditory processing of contextual sounds in typical development

In control animals, sex differences in mismatch processing emerged only in adulthood. Adult male rats exhibited greater iMM and iRS than females across IC divisions (Fig 5, upper panel). Similarly, auditory processing in the human midbrain becomes sexually dimorphic by adulthood [70]. These differences reflected increased CAS-elicited activity, reduced STD-evoked responses (Fig 3, upper panel), and faster adaptation to repetitive stimulation. Previous studies also reported sex differences in rats’ contextual discrimination. Males acquired context regulation over reward- and novelty-seeking behaviors more rapidly than females, suggesting enhanced neural attenuation to stable scenarios [71].

At the population level, we observed that from the prepubertal stage (P30–45) to adulthood (P65–120), iMM increased alongside a reduction in iRS, as well as a shift toward positive iPE values at low intensities in the non-lemniscal region of the IC (Fig 5, mid panel). Prenatal VPA exposure did not affect iPE emergence. An insightful study in mice [72] recently examined the development of neuronal mismatch (or stimulus-specific adaptation) across the auditory neuraxis and reported a decrease in neuronal mismatch with age up to P50 in the lemniscal IC.

Notably, in our study, a reduction in neuronal mismatch was only observed as a function of age in control females, and this occurred in both lemniscal and non-lemniscal IC (Fig 6). Several factors may explain this divergence. We used anesthetized rats, distinguished groups by sex, anatomical location, and age (including adult animals), and decomposed neuronal mismatch into its predictive components, repetition suppression and prediction error. Furthermore, our study employed a hierarchical statistical analysis, spanning from the mixed-effects model to population-level and experimental group post hoc comparisons, thereby avoiding the masking of group-specific differences. Future studies including awake animals, and similar experimental and analytical procedures, should help reach consensus regarding these differences.

Similar to animal studies, human research has reported heterogeneous findings, with some studies showing increases [73–75] and others reporting reductions in mismatch negativity as a function of age [76, 77]. Differences in DEV types, recording sites, and the confounding effects of pooling sexes likely contribute to the variability across mismatch studies.

Control rats of both sexes also showed age-related slowing and eventual loss of adaptation to repetitive stimulation in the non-lemniscal and lemniscal divisions, respectively (Fig 8). During prepuberty, neural mechanisms supporting auditory discrimination remain immature as sensory regularity patterns are still forming [76–78]. As the brain internalizes these regularities, change detection improves and reaches optimal performance in adulthood.

### Effects of prenatal VPA exposure on contextual processing

Our population-level findings of reduced iMM and iPE, alongside increased iRS (Fig 5, lower panel), are consistent with predictive coding accounts that posit *hyper-priors* and *diminished precision-weighting of prediction errors* in autism [3]. Under this framework, overly rigid top–down predictions and attenuated gain on prediction error signals may lead to an underestimation of discrepancies between expected and actual input, thereby dampening mismatch responses and impairing model updating. Then, an enhanced repetition suppression may emerge as repeated stimuli confirm strongly weighted predictions, resulting in an overly reduced neural responses to repeated input. This imbalance may explain the strong preference for predictability often observed in autism [3]. VPA-exposed rats display heightened stereotyped behaviors, including repetitive grooming and circling [79–81], likely as strategies to impose predictability in an unpredictable environment.

Our single-unit findings, considered in the context of previously reported behavioral evidence, suggest a potential mechanistic relationship between atypical predictive processing and core sensory and behavioral features of autism, a relationship that warrants investigation in future studies.

Our single-unit findings, in the context of previously reported behavioral evidence, point to a potential mechanistic relationship between atypical predictive processing and core sensory and behavioral features of autism that should be explored in future experiments.

Group-level analyses revealed pronounced sex- and age-specific effects of prenatal VPA exposure on mismatch processing (Fig 6). Control prepubertal rats showed no auditory sex differences. In contrast, VPA exposure introduced sex differences at this age and altered the developmental trajectory of contextual processing across IC divisions, producing distinct patterns in females and males. Previous studies reported that motor and sensory outcomes in VPA rats diverge by sex and developmental stage [82]. These findings highlight the heterogeneous developmental profiles and sex-specific trajectories characteristic of autism phenotypes.

**Fig 9:**
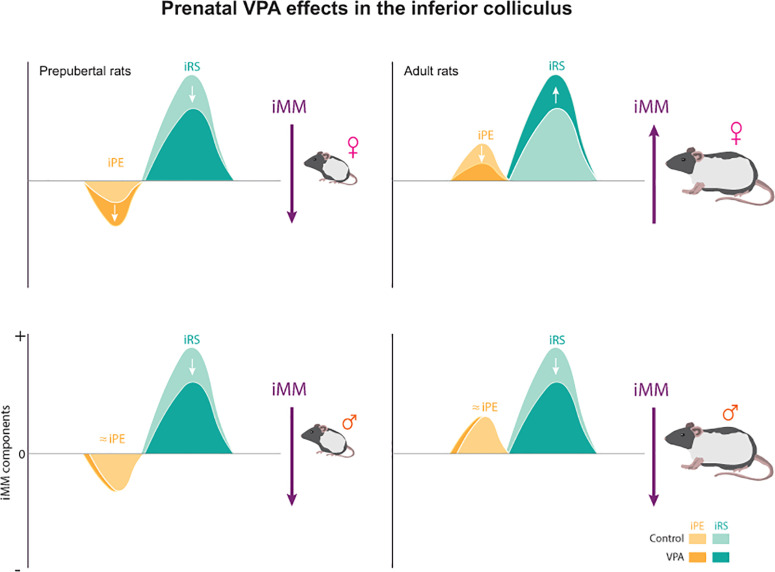
Summary diagram of major effects of *in utero* VPA exposure on predictive processing. The left panel illustrates findings in prepubertal rats, which exhibit negative iPE. Overall, VPA-exposed prepubertal rats of both sexes show reduced iRS and iMM. The right panel illustrates adult responses, where iPE becomes positive. In adult females, VPA exposure increases iRS and iMM but reduces iPE across both age groups. In contrast, VPA-exposed adult males show reductions in both iRS and iMM. This diagram summarizes the primary observations in the non-lemniscal IC.

Notably, group-level analyses revealed effects that were masked at the population level. The enhanced iRS observed at the population scale was primarily driven by VPA-exposed adult female rats, whereas reduced iRS and iMM were consistently present in males and prepubertal females (Fig 9). Reduced adaptation to repetitive tones, underlying attenuated mismatch signals, is broadly supported by existing literature in autism research in humans [12,83,84] and in rodent models of neurodevelopmental disorders, including fragile X syndrome [85–87]. From a predictive coding perspective, these findings may reflect attenuated priors, or *hypo-priors*. When predictions are weak, repeated stimulation continues to evoke robust neural responses, resulting in reduced adaptation, while prediction error signals remain relatively intact [3,28].

The heterogeneity of experimental designs and analytical approaches in autism research, combined with the divergent neurodevelopmental trajectories of the condition itself, likely gives rise to distinct neural phenotypes. These phenotypes may be better captured by different predictive coding models in an age- and sex-dependent manner.

### Female-specific divergences in contextual sound processing

Our sample of VPA adult females showed increased iMM and iRS, but reduced iPE across developmental stages (Fig 9). This pattern is consistent with *hyper-priors* combined with a *moderately reduced precision-weighting on prediction error signals*. Overly precise priors strongly suppress responses to repeated standards, resulting in enhanced iRS. But a partial reduction in the precision of prediction error signals attenuates the neural response to deviants, leading to reduced iPE, without eliminating it. Because the standard response is strongly suppressed, the contrast between deviant and standard responses remains large, producing an elevated iMM.

Recent investigations have begun to highlight sex-dependent differences in the VPA rodent model of autism [88–92]. Female rodents prenatally exposed to VPA have shown elevated spontaneous cortical and hippocampal activity [92], increased neuroinflammatory responses [91], superior performance in the Morris water maze [90], sex-specific patterns of gene expression across brain regions [89] and enhanced social and repetitive behaviors [88], compared to male counterparts. In line with this evidence, our findings support the presence of sex-specific differences, suggesting a female-specific pattern of neuronal and behavioral features of autism-like signs.

As most studies on perceptual sensitivity have focused on males, female-specific auditory phenotypes remain elusive. Recent evidence suggests that autistic females display intermediate mismatch responses between autistic males and neurotypical individuals [16]. Our findings support the presence of a distinct auditory discrimination phenotype in female VPA rats.

### The role of precision-weighting in atypical predictive function

Under predictive coding, increasing the gain of prior predictions or prediction errors, known as precision weighting, relies on post synaptic gain neuromodulation [19,93,94]. Neuromodulators assign confidence to predictions in volatile scenarios and to prediction errors in stable contexts [19,95]. Flexibly adjusting the importance of predictions and errors is atypical in autism [23,24,26,28,96–98], potentially due to unusual post-synaptic dopamine or acetylcholine gain along the auditory hierarchy [21,94,99,100]. Dopamine reduces DEV-evoked activity [46], while acetylcholine enhances STD-evoked responses [101], both lowering neuronal mismatch in the rat IC. Disruption in these systems [27,102] may impair predictive function in VPA rats. Indeed, our results show reduced neuronal mismatch and repetition suppression driven by weaker responses to STD stimuli, except in VPA adult females, which exhibited increased neuronal mismatch and repetition suppression. This unexpected pattern warrants further investigation into sex-specific effects of VPA on post-synaptic gain. Divergent developmental trajectories of precision weighing between sexes likely contribute to the heterogeneity of auditory phenotypes in autism.

### Structural and functional alterations in the auditory pathway of VPA rats

Previous studies have reported VPA-induced anatomical and physiological changes throughout the auditory hierarchy. Subcortically, VPA rats show reduced ascending projections, fewer neurons, hyper-responsiveness to pure tones, and weaker responses to complex sounds [32,34,38,103–105]. In the auditory cortex, they exhibit degraded temporal processing, deficient speech processing, and fewer cortico-collicular projections [52,106,107]. Disrupted excitatory/inhibitory balance [108–110] and altered connectivity contribute to the widespread anomalies observed across neuronal responses [52]. These alterations likely underlie the atypical context-dependent processing we observed in the IC of VPA rats. Behaviorally, VPA rats display autism-like features and atypical context-dependent discrimination across social and non-social domains [30,31,71]. While VPA-induced changes in neuronal mismatch have not been extensively studied, one report in adult VPA marmosets found weaker global predictions reflected as a smaller mismatch response [111], though sex and age effects remain elusive.

### Limitations and future directions

We conducted our IC recordings under urethane anesthesia, which is known to influence some aspects of prediction error responses [41,49]. While anesthesia provides stable recordings and controlled conditions, it can also alter brain activity, potentially masking the true dynamics of spontaneous activity and the excitation/inhibition balance. Previous studies have reported higher spontaneous and evoked firing rates in the awake rabbit IC compared to anesthetized states [112, 113]. In awake, behaving animals, increased spontaneous firing is linked to reduced adaptation [49,114], and attentional engagement can modulate both spontaneous and evoked responses [115].

Importantly, urethane is considered one of the least disruptive anesthetics for auditory research. Compared to barbiturates, it affects fewer neuronal properties and minimally impacts vital functions, excitatory/inhibitory balance, and synaptic timing [116–118].

Nonetheless, anesthesia limits our ability to fully capture the dynamic neural processes of natural, awake states. Future research should investigate prediction error processing in awake, behaving animals, particularly those prenatally exposed to VPA, to better reflect real-world brain function. Incorporating behavioral paradigms would also clarify how IC responses relate to task-relevant behaviors and higher cognitive functions, such as attention, prediction, and decision-making.

Another limitation is the lack of neuronal identity for the recorded IC cells. While cortical neurons can often be classified by spike shape [72,119], this approach is not feasible in the IC because there is no bimodal distribution of peak-to-trough times, despite the presence of inhibitory neurons [72,120,121]. Identifying cell types would require intracellular recordings [122], which are technically demanding and time-consuming in vivo.

Although the VPA model exhibits several behavioral and sensory features relevant to autism, we acknowledge the inherent limitations of animal models in distinguishing construct-specific effects from broader neurodevelopmental disruptions. Our objective was to identify neural response patterns that inform theoretical accounts of autistic perception, rather than to isolate electrophysiological markers unique to autism. We interpreted the observed divergences in neuronal mismatch within the framework of predictive coding, and contextualized them using converging evidence from human studies of autism and other neurodevelopmental models, such as fragile X syndrome. These parallels indicate that the findings are relevant to autism-related phenotypes, though not specific to autism itself. We therefore emphasize the value of these results in advancing mechanistic insight into disrupted auditory predictive processing in autism, while recognizing the translational limitations of the VPA model.

Despite these limitations, our findings reveal clear and robust differences between VPA-exposed and control animals. While these effects may differ in magnitude or character under awake conditions, they provide a strong foundation for future investigations across the auditory pathway.

## Materials and methods

We performed single-unit recordings in the IC of 83 anesthetized Long-Evans rats: 39 from a control rat group, consisting of prepubertal females (*n* = 9), prepubertal males (*n* = 11), adult females (*n* = 9) and adult males (*n* = 10), and 44 from a rat group given valproic acid *in utero*, consisting of prepubertal females (*n* = 12), prepubertal males (*n* = 11), adult females (*n* = 10) and adult males (*n* = 11). Developmental stages were defined as Sengupta [48], with prepuberty ranging P30–45, and adulthood ranging P65–120.

Experimental procedures were performed at the University of Salamanca, in accordance with the guidelines of the European Communities Directive (86/609/EEC, 2003/65/EC and 2010/63/EU) and the RD 53/2013 Spanish legislation for the use and care of animals. All the details of the study were approved by the Bioethics Committee of the University of Salamanca (ref# USAL-ID-574 and USAL-ID-1193).

### Generation of the control and VPA rat groups

To produce the VPA rat group, 8 control female adults with controlled estrous cycles were mated with 8 control male adults overnight, and the first day of pregnancy was determined by vaginal cytology. Since previous studies on *in utero* effects of VPA administration set a prenatal dose ranging approximately 250−600 mg/kg capable of inducing autism-like symptoms in rodent litters [30,31], we gave pregnant females an intraperitoneal injection of 400 mg/kg VPA on gestational day 12.5 [30,31,123–125]. We avoided using higher doses, e.g., 500−600 mg/kg, as these increase complete fetal resorptions [30,31,126–128]. The solution was prepared with 250 mg/mL VPA (Sigma) dissolved in 0.9% saline, pH 7.4. Female rats were monitored over gestation in the animal care facility until delivery, and the offspring were weaned on P21–23. Litters were housed with same-sex littermates in groups of 2–3 until reaching prepuberty or adulthood, which are the developmental stages selected for single-unit recordings.

The VPA rat group exhibited minor tail malformations due to prenatal exposure to VPA, as reported in previous studies [129]. We did not specifically quantify behavior or conduct formal behavioral tests, but we monitored the animals daily in their housing cages. Treated animals exhibited atypical locomotor patterns, repetitive behaviors, and stereotypic-like activity, consistent with previous reports [130–132].

The control rat group were either bred in our animal facility until prepuberty or adulthood, or purchased at the required age prior to electrophysiological recordings. A total of 37 control rats of both sexes and ages were purchased (Janvier, Spain). Out of the total purchased rats, 3 control female and male adults were mated overnight, defining the first day of pregnancy in females by vaginal cytology prior to intraperitoneally injecting 0.9% saline on the gestational day 12.5. The offspring received identical animal care as previously described for VPA litters, and purchased rats were housed by sex and age in groups of 2 or 3 prior to surgical procedures. Control and VPA rat groups were under a 12-h light/12-h dark cycle, with *ad libitum* access to food and water.

To minimize potential confounds, pups from the same litter were assigned to different experimental groups varying in sex, age, and exposure, ensuring that these factors were not conflated with litter identity. Litters were evenly distributed across experimental groups and, when feasible, contributed animals to multiple conditions to reduce systematic bias from litter effects. While all pups from a given litter shared the same prenatal treatment (control or VPA), the study design minimized the likelihood of confounding by litter. Because most control animals were purchased rather than born in-house, potential litter effects were further mitigated in this group.

Specifically, the saline-injected, in-house control animals included three rats in the control prepubertal female group, two in the control prepubertal male group, two in the control adult male group, and one in the control adult female group. Statistical comparisons between purchased and in-house control rats revealed no significant differences in spontaneous activity, evoked responses, or predictive indices across divisions or sound levels (Wilcoxon rank-sum tests with Bonferroni corrections; all *p > *0.05). Although including litter as a random effect could address any residual shared variance in future studies, the small number of in-house control animals and the lack of observed statistical differences suggest that litter-related effects were negligible.

### Surgical procedures

The surgical and recording procedures were as described in previous investigations [41,45,46]. Surgical anesthesia was induced with an intraperitoneal injection of urethane, 1.5 g/kg, ensuring a stable deep anesthetic level, with supplementary intraperitoneal doses of urethane approximately 0.5 g/kg when the rat recovered pedal withdrawal reﬂexes. Urethane was chosen over other anesthetic agents because it preserves normal neural activity better, having a modest, balanced eﬀect on inhibitory and excitatory synapses [45,117]. Normal hearing of rats was veriﬁed by recording the auditory brainstem responses subcutaneously with needle electrodes. An RZ6 Multi I/O Processor (Tucker-Davis Technologies) was used to acquire the auditory brainstem response, which was processed with BioSig software (Tucker-Davis Technologies) before the beginning of each experiment. Stimuli used to elicit the auditory brainstem response consisted of 0.1 ms clicks at a rate of 21 clicks/s, delivered monaurally to the right ear in 10 dB steps, from 10 to 90 decibels of sound pressure level (dB SPL), using a closed system through a Beyer DT-770 earphone (0.1–45 kHz) ﬁtted with a custom-made cone and coupled to a small tube (12 G) sealed in the ear.

Control and VPA rats presented similar auditory thresholds, since differences across sex, age and exposure were quantified as non-significant using *ranksum* function in MATLAB. This ensured the normal hearing of the animals prior to experimental procedures.

Once normal hearing had been conﬁrmed, a cannula was installed in the trachea to provide artiﬁcial ventilation and the expiratory CO_2_, was monitored, as urethane depresses respiratory function. Likewise, to maintain body temperature at 37 ± 1 °C, a rectal thermometer was used to control a homeothermic blanket system (Cibertec) on which the animal was placed. The head was stabilized in a stereotaxic frame with a bite bar and two hollow specula replaced the ear bars. A sound delivery system was accommodated within the right hollow speculum. Eyes were protected with a drop of ophthalmic gel. To prevent excessive bronchial secretions, 0.1 mg/kg of atropine sulfate was administered subcutaneously. To ameliorate brain edema, 0.25 mg/kg of dexamethasone was injected intramuscularly. To prevent dehydration, we administered 5 ml of glucosaline solution subcutaneously. The scalp was shaved, and the exposed skin was disinfected with povidone-iodine. Using a scalpel, an incision was opened along the midline to expose the skull, and the periosteum covering the parietal, and the most rostral part of the occipital bones was retracted. Using a dental drill, a round craniotomy was performed in the caudal part of the left parietal bone, just rostral to the lambdoid suture and lateral to the sagittal suture, thereby exposing the cerebral cortex overlying the left IC. The exposed dura was removed, and the tissue beneath it was covered with 2% agar to prevent desiccation during the recording session.

### Single-neuron electrophysiological recordings

Glass-coated tungsten microelectrodes were used to record the extracellular activity of IC neurons. These were crafted with a tip impedance of 1.5–3.5 MΩ at 1 kHz following the protocol detailed in our previous studies [41,42,50]. Experiments were performed inside a sound-insulated and electrically shielded chamber. The microelectrode was mounted on a holder over the exposed cortex, forming an angle of 20° perpendicularly rostral to the coronal plane. Using a piezoelectric micromanipulator (Sensapex), the electrode was inserted into the brain while measuring the penetration depth until strong spiking activity synchronized with the train of searching stimuli could be identified.

All sound stimuli were generated using an RZ6 Multi I/O Processor (Tucker-Davis Technologies) and custom software programmed with OpenEx Suite (Tucker-Davis Technologies) and MATLAB (MathWorks). Searching for evoked auditory neuronal responses from the IC, white noise bursts and sinusoidal pure tones of 75 ms duration with 5 ms rise–fall ramps were presented while varying stimuli parameters manually to prevent frequency-specific adaptation. Once the activity of a single neuron was clearly isolated, only pure tones were used to record the experimental stimulation protocols, which ran at four stimuli per second. Stimuli were delivered monaurally to the ear contralateral to the left IC through a close-field speaker. We calibrated the speaker using a 0.25-inch condenser microphone (model 4136, Brüel and Kjær) and a dynamic signal analyzer (Photon+, Brüel and Kjær) to ensure a flat spectrum up to 76 ± 3 dB SPL between 0.5 and 45 kHz and that the second and third signal harmonics were at least 40 dB lower than the fundamental at the loudest output level.

Analog signals were digitized with a RZ6 Multi I/O Processor, a RA16PA Medusa Preampliﬁer, and a ZC16 headstage (Tucker-Davis Technologies) at 12 kHz sampling rate and ampliﬁed 251×. Neurophysiological signals of spiking activity were bandpass ﬁltered between 0.5 and 3 kHz using a second-order Butterworth ﬁlter. Stimulus generation, neuronal response processing, and visualization were controlled online with custom software created with the OpenEx suite (Tucker-Davis Technologies) and MATLAB. A unilateral threshold for automatic action potential detection was manually set at approximately 2–3 STD deviations of the background noise. Spike waveforms were displayed on the screen and overlaid on each other in a pile plot to facilitate isolation of single unit activity. The recorded action potentials were considered to belong to a single unit only when all spike waveforms were identical, clearly separable from other smaller units and the background noise, and the spike amplitude-to-noise ratio of the average waveform was larger than 5.


$$\text{Signal/Noise Ratio}=\frac{\text{max }\left(\overset{―}{\text{x }}\left(\text{waveforms}\right)\right)\text{ – }min\left(\overset{―}{\text{x }}\left(\text{waveforms}\right)\right)}{\text{Std }\left(\text{waveforms}\right)}$$

A map of response magnitude for each frequency–intensity combination was ﬁrst computed, representing the receptive ﬁeld of the single unit in a frequency response area (Fig 1C). The stimulation protocol to obtain the frequency response area consisted of a sequence of sinusoidal pure tones ranging between 0.7 and 44 kHz, 75 ms of duration with 5 ms rise–fall ramps, presented at a 4 Hz rate, randomly varying frequency and intensity (3–5 repetitions of all tones).

Then, 10 frequencies separated by 0.5 octave steps at a ﬁxed sound intensity (usually 10–20 dB above minimal response threshold) were selected so that at least two consecutive tones fell within the excitatory region of the frequency response area. These 10 tones were used to generate the cascade control sequences (Fig 1B), and adjacent pairs within the excitatory region of the frequency response area were used to create oddball sequences (Fig 1B). All sequences were 400 tones in length, presented at a constant rate of 4 Hz and at a constant intensity between 10- and 70-dB SPL, in steps of 10 dB SPL.

The auditory oddball paradigm configures *standard* and *deviant* conditions, where repetitive stimuli are interrupted by novel tones [17]. The cascade control sequence, referred to as *cascade* condition, generates the control condition by embedding DEV tones in a pseudorandom manner [39]. The difference between DEV and STD conditions results in MMN [17,41]. Under predictive coding, MMN reflects the combination of a repetition suppression, or neural adaptation to repetitive sensory signals, when external signals match prior predictions. However, MMN also represents a prediction error, or the increase in neural response to unexpected cues, when external inputs differ from prior predictions [41].

A ﬁrst complete experimental set, composed of oddball and cascade control sequences in both their “ascending” and “descending” versions (Fig 1B), was presented at a chosen intensity. Whenever it was possible to maintain a certain neuron isolated over a long period of time, another set would be presented at a diﬀerent intensity. Usually, one intensity would be <40 dB SPL (low) and the other ≥40 dB SPL (high), but this depended on the receptive ﬁeld of each neuron as shown by the frequency response area. The presentation of a set at diﬀerent intensity levels did not follow any particular order. Sometimes the stability of the signal allowed recordings to be made with multiple complete sets of sequences at several levels of intensity. However, such occurrences were relatively rare, since our recording protocol did not permit reisolation attempts. When signal quality dropped below STDs, the recording was interrupted, and the incomplete set discarded.

A 10% probability was used to pseudorandomly distribute tones across control sequences within chunks of 10 stimuli, as well as to scatter DEV tones across the oddball sequence. Since only the last STD tones before a DEV were considered for analysis, this method resulted in 40 trials of DEV, STD and CAS for each given tone of interest. In the oddball paradigm, the ﬁrst 10 tones were always STD, and a minimum of 3 STD preceded each DEV, which were pseudorandomly presented with a 10% probability. DEV and STD conditions of the oddball paradigm will be used to obtain neuronal mismatch measurements.

One limitation of the neuronal mismatch measurements obtained using the oddball paradigm is that the activity relating to high-order processes of prediction error signaling cannot be distinguished from lower-order eﬀects such as frequency-speciﬁc adaptation [133]. The controls allow the relative contribution of both higher- and lower-order processes to the overall mismatch response to be assessed [39]. These controls of the auditory oddball paradigm are tone sequences that must meet three criteria: (1) to feature the same tone of interest with the same presentation probability as that of the DEV (e.g., 10%); (2) to induce an equivalent state of refractoriness by presenting the same rate of stimulus per second (e.g., 4 Hz); and (3) to present no recurrent repetition of any individual stimulus, especially the tone of interest [134].

These three presentation criteria prevent the short-term dynamics of frequency-speciﬁc adaptation in the IC while keeping the long-term dynamics at a minimum. Eliminating this minimum would be impractical due to the long-lasting eﬀects of frequency-speciﬁc adaptation, which in the rat IC have been reported at presentation rates of up to one repetition per second [134]. Consequently, even DEV-evoked responses manifest some marginal frequency-speciﬁc adaptation [42] and will similarly aﬀect tones in a control sequence when presented at equal rate and probability. Note that the objective of these criteria is not to avoid inducing frequency-speciﬁc adaptation altogether during the control sequences but to guarantee that the response to the tone of interest is subject to similar amounts of refractoriness and adaptation in DEV and CAS conditions, thereby allowing comparisons to reveal the inﬂuence of higher-order processes.

Hence, we can assess the portion of the mismatch response (DEV–STD) that can be attributed to frequency-speciﬁc adaptation induced during the STD train [135]. When the auditory-evoked response is similar or higher during the control than in DEV, then the mismatch response can be fully accounted for by repetition suppression, and no higher-order process of prediction error signaling can be deduced (e.g., DEV ≤ CAS; Fig 1B). Otherwise, a stronger response to DEV than to the control unveils a component of the mismatch response that can be better explained by prediction error signaling (e.g., DEV > CAS; Fig 1B).

To dissociate the relative contribution of frequency-speciﬁc eﬀects from genuine prediction error signaling (Fig 1B), we generated the cascade control sequences for our oddball paradigms. We used CAS instead of the many standards control in this experimental design. The many standards control presents the tone of interest embedded in a random sequence of assorted tones, where each tone shares the same 10% presentation probability as DEV in the oddball paradigm. However, some authors have argued that the many standards control is not fully comparable with the oddball paradigm, because the disorganized succession of tones creates a context of uncertainty that never allows the generation of high-precision predictions, whereas STD does [39,136]. Additionally, each tone is preceded by a diﬀerent tone from trial to trial, which makes it diﬃcult to control any possible eﬀects of spectral processing caused by the diﬀerences in frequency steps.

CAS tries to overcome the alleged caveats of many standards control by presenting tones in a regular fashion, that is, in an increasing or a decreasing frequency succession or scale. Thus, the stimulus of interest conforms to a regularity, as opposed to DEV, but not a regularity established by repetition, contrary to STD, thereby restricting possible frequency-speciﬁc eﬀects to a minimum while making CAS a more ﬁtted control than many standards control [39]. Stimuli (DEV, CAS, and STD conditions) and experimental groups were interleaved across recording sessions to control for temporal drift and minimize batch effects.

### Data analysis and code accessibility

All data analysis and data visualization were performed with MATLAB software, using the built-in functions, the Statistics and Machine Learning toolbox, and custom scripts and functions developed in our laboratory. All custom MATLAB and statistical scripts used in data preprocessing, analysis, and visualization are publicly available without restriction at Zenodo (https://doi.org/10.5281/zenodo.15914264), under a MIT license.

Taking the 40 trials available for each tone within each condition (DEV, STD and CAS), a peristimulus time histogram was computed to represent the action potential density over time in spikes per second from −75 to 250 ms around stimulus onset. This histogram was smoothed with a 6 ms Gaussian kernel (*ksdensity* function in MATLAB) in 1 ms steps to estimate the spike- density function over time. The baseline spontaneous ﬁring rate was determined as the average ﬁring rate (in spikes/s) during the 75 ms preceding stimulus onset. The excitatory response was measured as the area below the spike-density function and above the baseline spontaneous ﬁring rate, between 0 and 180 ms after stimulus onset. In other words, the average level of spontaneous ﬁring within the −75–0 ms time window was subtracted from the activity recorded within the 0–180 ms time window, thereby obtaining a measurement of the evoked ﬁring activity that is referred to as the baseline-corrected spike count.

After subtracting the spontaneous activity, we used a Monte Carlo method to ﬁnd statistically signiﬁcant responses evoked by sound within the resulting baseline-corrected spike counts. This approach consists of a probability simulation that withdraws numerical values from several random samplings. First, 1,000 peristimulus time histograms were simulated using a Poisson model with a constant ﬁring rate equal to the baseline spontaneous ﬁring rate. With this collection of histograms, we generated a null distribution of baseline-corrected spike counts. Finally, we computed the *p* value of the original baseline-corrected spike count as p = (g + 1)/(*N* + 1), where *g* is the count of null measures greater than or equal to the baseline-corrected spike count and *N* = 1,000 is the size of the null sample. Hence, the Monte Carlo method allowed us to remove any unit-frequency combinations without signiﬁcant ﬁring activity evoked in response to at least one of the conditions in each experimental set (DEV, STD or CAS).

To compute predictive processing indices, CAS was introduced to control for the repetition eﬀects of the oddball paradigm, allowing a dissociation of frequency-speciﬁc adaptation into prediction error and repetition suppression components. To adequately compare between responses from diﬀerent neurons, we normalized the spike count evoked by each tone in DEV, STD, and CAS as follows:


$$\text{DEV }Normalized\text{\textasciitilde{}}=\frac{\text{DEV}}{N}\text{;}$$

$$\text{STD}Normalized\text{\textasciitilde{}}=\frac{\text{STD}}{N}\text{; }$$

$$\text{CAS}Normalized\text{\textasciitilde{}}=\frac{\text{CAS}}{N}\text{,}$$

where


$$N=\sqrt{\text{ DEV² + STD² + CAS²}}.$$

From these normalized responses, we computed iMM, iRS, and iPE as follows:


$$\text{iMM}={\text{DEV}}_{Normalized}-{\text{STD}}_{Normalized};$$

$$\text{iRS}={\text{CAS}}_{Normalized}-{\text{STD}}_{Normalized};$$

$$\text{iPE}={\text{DEV}}_{Normalized}-{\text{CAS}}_{Normalized}.$$

These indices range between −1 and 1. The neuronal mismatch is largely equivalent to the classic Common Stimulus-Speciﬁc Adaptation Index [41,135].

### Statistical analysis

Statistical analysis was carried out using distribution-free, non-parametric tests. These included the Friedman test for baseline-corrected spike counts and normalized responses to DEV, STD and CAS, as well as for the iMM, iRS, and iPE (Table 1, significant values are highlighted in bold). For multiple comparison tests, *p* values were corrected for false discovery rate 0.1 using the Benjamini–Hochberg method.

To investigate how auditory division, sound level, sex, age, and prenatal VPA exposure influenced single-neuron activity, we fit a *generalized linear mixed-effects models* separately for spontaneous activity, evoked brain responses (DEV, CAS, and STD), and predictive indices (iMM, iRS, and iPE) [137]. Fixed effects included division, level, sex, age, and exposure. Each model incorporated random intercepts for subject ID (*n* = 83) and random slopes for division, level, and exposure to capture both between-subject and within-subject variability.

Including random effects was essential given the considerable inter-individual variability inherent to neurodevelopmental research, particularly in developmental and prenatal exposure models. The random effects accounted for between-subject variability, allowing the fixed effects to estimate population-level predictors accurately.

The intraclass correlation coefficient quantified the proportion of variance attributable to between-subject variability. According to the normed and standardized intraclass correlation coefficient [138], values below 0.40 indicate poor concordance, values between 0.40 and 0.59 are considered fair, values from 0.60 to 0.74 are good, and values of 0.75 or higher represent excellent concordance. Higher intraclass correlation coefficient values indicated that a larger proportion of the total variance was attributable to consistent between-subject differences driven by the experimental variables (sex, age, and exposure, across each division and sound level), rather than random or within-subject variability. The models were specified as follows:

For spontaneous activity:


Spontaneousactivity~Division×Sex×Age×Exposure+(1+Division+Exposure|SubjectID)

Distribution: Gamma; Link: log.

For DEV-evoked responses:


DEV~Division×Level×Sex×Age×Exposure+(1+Division+Level+Exposure|SubjectID)

Distribution: Gamma; Link: log;

For CAS-evoked responses:


Control~Division×Level×Sex×Age×Exposure+(1+Division+Level+Exposure|SubjectID)

Distribution: Gamma; Link: log.

For STD-evoked responses:


STD~Division×Level×Sex×Age×Exposure+(1+Division+Level+Exposure|SubjectID)

Distribution: Normal; Link: identity; FitMethod: Laplace.

For iMM:


iMM~Division×Level×Sex×Age×Exposure+(1+Division+Level+Exposure|SubjectID)

Distribution: Normal; Link: identity; FitMethod: Laplace.

For iRS:


iRS~Division×Level×Sex×Age×Exposure+(1+Division+Level+Exposure|SubjectID)

Distribution: Normal; Link: identity; FitMethod: Laplace.

For iPE:


iPE~Division×Level×Sex×Age×Exposure+(1+Division+Level+Exposure|SubjectID)

Distribution: Normal; Link: identity; FitMethod: Laplace.

For spontaneous activity, DEV-, and CAS-evoked responses, we used a gamma distribution with a log link because the data were strictly positive and non-parametric. STD-elicited responses and predictive indices, although non-parametric, approximated normal distributions after normalization, supporting the use of normal distributions with identity links. We applied the Laplace approximation for these models to ensure accurate likelihood estimation given the complex random effects structure.

To ensure robust model performance and reliable estimation of both fixed and random effects, we evaluated several metrics. The intraclass correlation coefficient assessed the proportion of variance attributable to between-subject variability, with higher values reflecting better explanatory power of the fixed effects and random slopes [138]. We also computed the cross-validated median absolute error, a robust measure of predictive error less sensitive to outliers, a critical consideration given the biological variability inherent to single-neuron data [139]. We used the Akaike Information Criterion and Bayesian Information Criterion to assess overall model fit and parsimony [137]. Both criteria help select the best model for the data among candidate models, with lower values indicating better fit and greater parsimony.

For all fixed-effect estimates, we computed bootstrapped 95% confidence intervals using 500 iterations. This resampling approach accounted for uncertainty in parameter estimation and validated the robustness of our statistical inferences. Although computational demands limited the number of iterations, 500 resamples were sufficient to produce stable and reliable confidence intervals, in line with established resampling principles [140].

We specified random-effects structures based on the response type. For spontaneous activity, the model included random slopes for IC Division and Exposure. For elicited responses, we included random slopes for IC Division, Level, and Exposure. These structures increased intraclass correlation coefficients, indicating improved capture of subject-specific variability. Random-effect standard deviations were low, and correlations among random terms were consistently high (Table 7), supporting model stability.

**Table 7:** Generalized linear mixed-effect model outcome for random effects.

Name1	Name2	Type	Estimate
**Spontaneous activity**			
** **Intercept	Intercept	std	2.742
** **Division	Intercept	corr	–0.996
** **Exposure	Intercept	corr	–0.817
** **Division	Division	std	2.144
** **Exposure	Division	corr	0.837
** **Exposure	Exposure	std	1.553
** **Sqrt (Dispersion)			0.83
**DEV**			
** **Intercept	Intercept	std	0.187
** **Division	Intercept	corr	–0.650
** **Level	Intercept	corr	–0.880
** **Exposure	Intercept	corr	–0.981
** **Division	Division	std	0.107
** **Level	Division	corr	0.421
** **Exposure	Division	corr	0.639
** **Level	Level	std	0.192
** **Exposure	Level	corr	0.778
** **Exposure	Exposure	std	0.129
** **Sqrt (Dispersion)			0.056
**CAS**			
** **Intercept	Intercept	std	0.206
** **Division	Intercept	corr	–0.950
** **Level	Intercept	corr	–0.976
** **Exposure	Intercept	corr	–0.995
** **Division	Division	std	0.168
** **Level	Division	corr	0.883
** **Exposure	Division	corr	0.914
** **Level	Level	std	0.185
** **Exposure	Level	corr	0.985
** **Exposure	Exposure	std	0.158
** **Exposure	Exposure	std	0.129
** **Sqrt (Dispersion)			0.047
**STD**			
** **Intercept	Intercept	std	0.116
** **DIVISION	Intercept	corr	−0.998
** **Level	Intercept	corr	0.719
** **Exposure	Intercept	corr	−0.957
** **DIVISION	DIVISION	std	0.102
** **Level	DIVISION	corr	−0.76
** **Exposure	DIVISION	corr	0.972
** **Level	Level	std	0.041
** **Exposure	Level	corr	−0.89
** **Exposure	Exposure	std	0.075
** **Sqrt (Dispersion)			0.075
**iMM**			
** **Intercept	Intercept	std	0.189
** **Division	Intercept	corr	–0.734
** **Level	Intercept	corr	–0.375
** **Exposure	Intercept	corr	–0.958
** **Division	Division	std	0.123
** **Level	Division	corr	–0.248
** **Exposure	Division	corr	0.62
** **Level	Level	std	0.137
** **Exposure	Level	corr	0.32
** **Exposure	Exposure	std	0.111
** **Sqrt (Dispersion)			0.078
**iRS**			
** **Intercept	Intercept	std	0.157
** **Division	Intercept	corr	–0.999
** **Level	Intercept	corr	0.284
** **Exposure	Intercept	corr	–0.939
** **Division	Division	std	0.141
** **Level	Division	corr	–0.302
** **Exposure	Division	corr	0.946
** **Level	Level	std	0.039
** **Exposure	Level	corr	–0.596
** **Exposure	Exposure	std	0.083
** **Sqrt (Dispersion)			0.108
**iPE**			
** **Intercept	Intercept	std	0.006
** **DIVISION	Intercept	corr	–0.873
** **Level	Intercept	corr	–0.410
** **Exposure	Intercept	corr	–0.987
** **DIVISION	DIVISION	std	0.084
** **Level	DIVISION	corr	–0.087
** **Exposure	DIVISION	corr	0.784
** **Level	Level	std	0.062
** **Exposure	Level	corr	0.55
** **Exposure	Exposure	std	0.004
** **sqrt (Dispersion)			0.059

The “*Name1*” and “*Name2*” columns indicate the pair of random effects for which the covariance parameters were estimated (e.g., intercept or a factor such as Division or Exposure). The “*Type*” column specifies whether the estimate refers to a standard deviation (“std”) or a correlation (“corr”) between random effects. The “*Estimate*” column provides the numerical value of the corresponding standard deviation, correlation, or dispersion.

To further explore the effects identified by the mixed-effects models, we performed post hoc comparisons at two hierarchical levels. At the population level, we applied Wilcoxon rank-sum tests with Bonferroni correction to evaluate differences in spontaneous activity, evoked responses, and predictive indices across sex, age, and exposure within each division and sound level. At the group level, we conducted Wilcoxon rank-sum tests with false discovery rate correction to examine pairwise differences between subgroups defined by sex, age, and exposure while controlling for multiple comparisons within each division and sound level. This hierarchical strategy allowed us to detect both broad population-level trends and fine-grained, group-specific effects. Because between-subject variability was effectively explained by the modeled experimental factors and the random effects showed low residual variance, pooling neurons by experimental group for post hoc comparisons was statistically justified.

All generalized linear mixed-effects models were fit using MATLAB R2022b (MathWorks , Natick, MA), with custom scripts for model specification, diagnostics, and post hoc testing. Bootstrapping procedures were implemented using in-house code optimized for computational efficiency, and all multiple comparison corrections were conducted in MATLAB.

To analyze the time course of adaptation, we computed an averaged time course for all stimuli conditions. Then, we fit a power-law model, *y(t)=a t*^*b*^ + *c*, where *y(t)* represents the neuronal response at time *t*; *a* indicates the start of the response, or the first spike strength; *b* the sensitivity to repetitive stimuli, or long-term adaptation velocity; and *c* the steady-state value [50,141]. *R*^2^, or fit goodness (displayed in Table 5), indicated the responses that fit power-law dynamics in the control and VPA rat groups, explaining 67%–78% of the response variability within the inferior colliculus. To evaluate the variability of the fits, we used *interp1* MATLAB function to obtain 1,000 iterations of averaged CAS- and STD-evoked responses in the non-lemniscal IC at high-intensity levels. Sex, age and exposure differences, when possible, were evaluated with the MATLAB function *ranksum* (Table 6). Lastly, we computed half adaptation of averaged STD spikes by calculating half of the total decay of STD-evoked responses to evaluate short-term adaptation, as previously reported [50,141].

### Histology and neuroanatomical location of recording sites

At the end of each experiment, electrolytic lesions were inﬂicted by applying an electric current of 5 μA for 5 s through the recording electrode along the electrode track. If still alive after the conclusion of the experimental paradigms, animals were euthanized by injecting a lethal dose of pentobarbital. If still alive, animals were sacrificed by injecting a lethal dose of pentobarbital. After death was confirmed, they were decapitated. Brains were immediately immersed in a mixture of 4% formaldehyde in 0.1 M PB. After fixation, the neural tissue was cryoprotected in 30% sucrose and sectioned in the coronal plane at 40 μm thickness on a freezing microtome. Slices were stained with 0.1% cresyl violet to facilitate identiﬁcation of cytoarchitectural boundaries. Finally, the recorded neurons were assigned to one of the main subdivisions of the IC using the standard sections from a rat brain atlas as reference, as in our previous studies [41,42,44–46].

The main dataset is composed of 903 auditory neurons recorded in the IC of 83 anesthetized Long–Evans rats. Histological examination of the IC samples revealed that 426 neurons came from the central nucleus of the IC (the lemniscal division or lemniscal IC) comprised of 155 neurons from the control rat group: 50 neurons in prepubertal females; 34 neurons in prepubertal males; 27 neurons in adult females; and 44 neurons in adult males, and 271 neurons from the VPA rat group: 66 neurons in prepubertal females; 63 neurons in prepubertal males; 31 neurons in adult females; and 111 neurons in adult males.

The other 477 collicular neurons were in the cortical regions of the IC, the non-lemniscal division or non-lemniscal IC, represented by 242 neurons from the control rat group: 52 neurons in prepubertal females; 38 neurons in prepubertal males; 84 neurons in adult females; and 68 neurons in adult males, and 235 neurons from the VPA rat group: 59 neurons in prepubertal females; 97 neurons in prepubertal males; 49 neurons in adult females; and 30 neurons in adult males.

## Supporting information

S1 Data Data for spike-density functions to DEV, CAS, and STD conditions across lemniscal and non-lemniscal IC regions. DEV, deviant; CAS, cascade; STD, standard.
(XLSX).

S2 Data Data for population- and group-level spontaneous activity in the inferior colliculus. Group abbreviations: FpC = female prepubertal control, FaC = female adult control, MpC = male prepubertal control, MaC = male adult control, FpV = female prepubertal VPA, FaV = female adult VPA, MpV = male prepubertal VPA, MaV = male adult VPA. General abbreviations: IC, inferior colliculus; VPA, valproic acid.
(XLSX).

S3 Data Data for population-level spike count to deviant, cascade, and standard conditions. Abbreviations: IC, inferior colliculus; VPA, valproic acid; DEV, deviant; CAS, cascade; STD, standard.
(XLSX).

S4 Data Data for group-level spike count to deviant, cascade, and standard conditions. Group abbreviations: FpC = female prepubertal control, FaC = female adult control, MpC = male prepubertal control, MaC = male adult control, FpV = female prepubertal VPA, FaV = female adult VPA, MpV = male prepubertal VPA, MaV = male adult VPA. General abbreviations: IC, inferior colliculus; VPA, valproic acid; DEV, deviant; CAS, cascade; STD, standard.
(XLSX).

S5 Data Data for population-level predictive components of mismatch processing. Abbreviations: IC, inferior colliculus; VPA, valproic acid; iMM, neuronal mismatch index; iRS, repetition suppression index; iPE, prediction error index.
(XLSX).

S6 Data Data for group-level spike count to predictive components of mismatch processing. Group abbreviations: FpC = female prepubertal control, FaC = female adult control, MpC = male prepubertal control, MaC = male adult control, FpV = female prepubertal VPA, FaV = female adult VPA, MpV = male prepubertal VPA, MaV = male adult VPA. General abbreviations: IC, inferior colliculus; VPA, valproic acid; iMM, neuronal mismatch index; iRS, repetition suppression index; iPE, prediction error index.
(XLSX).

S7 Data Data for time course of adaptation in cascade and standard conditions in control and VPA-exposed rats. Group abbreviations: FpC = female prepubertal control, FaC = female adult control, MaC = male adult control, FpV = female prepubertal VPA, MpV = male prepubertal VPA. General abbreviations: IC, inferior colliculus; VPA, valproic acid; CAS, cascade; STD, standard.
(XLSX).

S8 Data Data for half adaptation to standard stimuli in control and VPA-exposed rats. Group abbreviations: FPC = female prepubertal control, FaC = female adult control, MpC = male prepubertal control, MaC = male adult control, FpV = female prepubertal VPA, FaV = female adult VPA, MpV = male prepubertal VPA, MaV = male adult VPA.
(XLSX).

## Abbreviations

CAS: cascade control
dB SPL: decibels sound pressure level
DEV: deviant
IC: Inferior colliculus
IC_L_: lemniscal inferior colliculus
IC_NL_: non-lemniscal inferior colliculus
iMM: index of neuronal mismatch
iPE: index of prediction error
iRS: index of repetition suppression
MMN: Mismatch negativity
P: postnatal days
STD: standard
VPA: Valproic acid
