# Supplementary material for: Contextual auditory processing in the inferior colliculus is affected in a sex- and age-dependent manner in the valproic acid-induced rat model of autism
Source: PLoS Biol. 2025 Aug 4;23(8):e3003309. doi: 10.1371/journal.pbio.3003309 (PMC12342314; doi:10.1371/journal.pbio.3003309)
Supplement: Attachment — Submitted filename: response to reviewers final 12-5-25.pdf [file pbio.3003309.s011.pdf]

Dear Manolo,

Thank you for your patience while your manuscript "Atypical predictive coding in the inferior colliculus of the valproic acid-induced rat model of autism: Sex, age and exposure differences" was peer-reviewed at PLOS Biology. It has now been evaluated by the PLOS Biology editors, an Academic Editor with relevant expertise, and by several independent reviewers.

In light of the reviews, which you will find at the end of this email, we would like to invite you to revise the work to thoroughly address the reviewers' reports.

As you will see below, the reviewers agree that the topic is interesting and the findings potentially important, but they mention a lot of concerns regarding the statistical analyses, the overall presentation, lack of validation of the animal model, lack of behavioral assessment and the fact that experiments have been conducted in anesthetized animals.
We encourage you to address all reviewer concerns, but understand that the concerns regarding the lack of behavioral data and the anesthesia may be very challenging to address. If you are not able to provide additional experimental data to address these points, please discuss both as limitations in the Discussion section.

Given the extent of revision needed, we cannot make a decision about publication until we have seen the revised manuscript and your response to the reviewers' comments. Your revised manuscript is likely to be sent for further evaluation by all or a subset of the reviewers.

In addition to these revisions, you will need to complete some formatting changes, which you will receive in a follow up email. A member of our team will be in touch with a set of requests shortly.

We expect to receive your revised manuscript within 3 months. Please email us (plosbiology@plos.org) if you have any questions or concerns, or would like to request an extension.

At this stage, your manuscript remains formally under active consideration at our journal; please notify us by email if you do not intend to submit a revision so that we may withdraw it.

IMPORTANT - SUBMITTING YOUR REVISION

Your revisions should address the specific points made by each reviewer. Please submit the following files along with your revised manuscript:

1. A 'Response to Reviewers' file - this should detail your responses to the editorial requests, present a point-by-point response to all of the reviewers' comments, and indicate the changes made to the manuscript.
*NOTE: In your point-by-point response to the reviewers, please provide the full context of each review. Do not selectively quote paragraphs or sentences to reply to. The entire set of reviewer comments should be present in full and each specific point should be responded to individually, point by point.
You should also cite any additional relevant literature that has been published since the original submission and mention any additional citations in your response.

2. In addition to a clean copy of the manuscript, please also upload a 'track-changes' version of your manuscript that specifies the edits made. This should be uploaded as a "Revised Article with Changes Highlighted" file type.

Re-submission Checklist
When you are ready to resubmit your revised manuscript, please refer to this re-submission checklist: https://plos.io/Biology_Checklist

To submit a revised version of your manuscript, please go to https://www.editorialmanager.com/pbiology/ and log in as an Author. Click the link labelled 'Submissions Needing Revision' where you will find your submission record.

Please make sure to read the following important policies and guidelines while preparing your revision:

Published Peer Review
Please note while forming your response, if your article is accepted, you may have the opportunity to make the peer review history publicly available. The record will include editor decision letters (with reviews) and your responses to reviewer comments. If eligible, we will contact you to opt in or out. Please see here for more details:
https://blogs.plos.org/plos/2019/05/plos-journals-now-open-for-published-peer-review/

PLOS Data Policy
Please note that as a condition of publication PLOS' data policy (http://journals.plos.org/plosbiology/s/data-availability) requires that you make available all data used to draw the conclusions arrived at in your manuscript. If you have not already done so, you must include any data used in your manuscript either in appropriate repositories, within the body of the manuscript, or as supporting information (N.B. this includes any numerical values that were used to generate graphs, histograms etc.). For an example see

here: http://www.plosbiology.org/article/info%3Adoi%2F10.1371%2Fjournal.pbio.1001908#s5

Blot and Gel Data Policy
We require the original, uncropped and minimally adjusted images supporting all blot and gel results reported in an article's figures or Supporting Information files. We will require these files before a manuscript can be accepted so please prepare them now, if you have not already uploaded them. Please carefully read our guidelines for how to prepare and upload this data: https://journals.plos.org/plosbiology/s/figures#loc-blot-and-gel-reporting-requirements

Protocols deposition
To enhance the reproducibility of your results, we recommend that if applicable you deposit your laboratory protocols in protocols.io, where a protocol can be assigned its own identifier (DOI) such that it can be cited independently in the future. Additionally, PLOS ONE offers an option for publishing peer-reviewed Lab Protocol articles, which describe protocols hosted on protocols.io. Read more information on sharing protocols at https://plos.org/protocols?utm_medium=editorial-email&utm_source=authorletters&utm_campaign=protocols

Thank you again for your submission to our journal. We hope that our editorial process has been constructive thus far, and we welcome your feedback at any time. Please don't hesitate to contact us if you have any questions or comments.

Sincerely,
Christian

Christian Schnell, PhD
Senior Editor
PLOS Biology
cschnell@plos.org

OUR RESPONSE

Dear Dr. Schnell, dear Christian

First of all, we would like to thank the 3 reviewers for their constructive criticisms, and you, as the editor, for giving us the opportunity to submit a significantly improved version of our manuscript. While it is not a frequent occurrence, it is a privilege to receive comments and reviews that are both thoughtful and enjoyable to read.

We are deeply grateful for the insightful observations and suggestions provided by the reviewers. Some of the comments were raised by more than one reviewer, which highlights their genuine constructive criticism. We truly believe these comments have been instrumental in enhancing the quality of this revised manuscript. As a result of the reviewers' analytical suggestions, the entire Results section has been thoroughly revised and recast. We are confident that we have addressed all the reviewers' concerns and that, as a result, the manuscript has substantially improved.

REVIEWS:

Reviewer #1: Summary:

In this study, the researchers conducted single-unit recordings in the inferior colliculus of anaesthetised animals to examine age- and sex-related variability in predictive coding in a rat model of autism. Specifically, they utilised valproic acid-induced (VPA) rat models within an oddball paradigm and non-repetitive cascade control sequences to investigate mismatch negativity. The rats were divided into multiple groups for this study: control and VPA rats, further categorised by sex and age (prepubertal and adult). The authors report several age- and sex-related differences in VPA rats, providing further evidence of the heterogeneity of autism-like symptoms and predictive coding.

Overall, the investigation of predictive coding in an autism model is highly valuable, given existing hypotheses on the subject. Additionally, the study originates from a group of researchers well known for their excellent work in predictive coding. However, several concerns limit the impact of this paper. After multiple readings, this reviewer found the data presentation overwhelming, with numerous comparisons (young vs. adult, male vs. female, loud vs. soft, VPA vs. non-VPA, lemniscal vs. non-lemniscal), making it difficult to discern any overarching conclusions. Further concerns are outlined below regarding the statistical approaches and the connection between the auditory and autistic phenotypes.

OUR RESPONSE:

Thank you very much for your supportive comments and appreciation of our work. We agree that the data is overwhelming, and we hope that the new analysis and presentation now simplify and enhance readability.

Major Concerns:

1. While VPA is known to increase the risk of autism-like symptoms in rats, how

was this confirmed? The methods section mentions that minor tail malformations are present in VPA rats, but no additional checks, such as behavioural studies, are referenced. The cited study on tail malformations describes the phenotype in rats dosed with 450 mg/kg (or higher), but not at 400 mg/kg, which was the dose used in this study. It would have been beneficial to assess whether the severity of the "autism" phenotype correlates with the neurophysiological measurements.

OUR RESPONSE:

We apologize for the previously brief description. In addition to observing tail malformations, and although we did not specifically quantify behavior or conduct formal behavioral tests, we monitored the animals daily in their housing cages. Treated animals exhibited atypical locomotor patterns, repetitive behaviors, and stereotypic-like activity, consistent with previous reports (e.g., Morales-Navas et al., 2024; Cezar et al., 2018). These observations are now detailed in the Methods section. Conducting a full behavioral analysis would have been time-consuming and fell outside the scope of the present study. Further, as also requested by Reviewer #3 and the editor, this limitation has been explicitly acknowledged in the Discussion section.

We apologize for the previous inaccuracies in the references. These have now been carefully revised and corrected. The dose of 400 mg/kg was selected based on previous studies that evaluated the potential effects of moderate dosages (Cohen et al., 2013; Cusmano & Mong, 2014; Wellmann et al., 2014). These doses were adjusted to reduce toxicity and minimize reabsorption issues in female rats, which had been observed at higher doses of 500 and 600 mg/kg (Vorhees, 1987; Favre et al., 2013).

2. In this study, recordings from individual neurons were pooled within each group (e.g., adult female VPA rats) for comparison, yet little mention is made of the variation between individual rats or litters within each group. It is essential to determine whether the main source of variability arises between litters, between individual rats, between treatment groups, or between neurons.

OUR RESPONSE:

We agree with the reviewer’s suggestion and, as also recommended by Reviewer #2, we consulted a colleague from the Department of Statistics and conducted a new analysis using a generalized linear mixed-effects model. This updated approach offers additional insights into between-individual variability and reinforces our original conclusions, thereby enhancing the robustness of

the analysis. We believe the revised findings provide a clearer understanding of the phenomenon and effectively address the reviewers' concerns.

Given the complexity of our design, spanning multiple biological factors (Division, Level, Sex, Age, Exposure) and repeated measures, we selected the generalized linear mixed-effects model as the primary analytical framework. This model effectively captured both fixed effects and random effects associated with SubjectID. Where appropriate, we incorporated random slopes to account for subject-specific variability across Division, Exposure, and Level, acknowledging that both spontaneous and evoked responses varied systematically with these factors.

This analytical strategy enabled us to generate interpretations that are both statistically rigorous and biologically meaningful while appropriately modelling the hierarchical structure of the data. Specifically, we grouped neurons by Exposure, Division, Level, and SubjectID to account for potential variability in neuronal responses among individual rats. Model fit and predictor evaluation indicated that variability was explained by the fixed effects rather than by within-animal differences, thus justifying the pooling of data by experimental group and neuronal level.

To minimize potential confounds, pups from the same litter were assigned to different experimental age (Prepubertal and Adult), sex (Female and Male), and exposure (Control and VPA) groups, ensuring that these effects of Sex, Age, and Exposure were not conflated with litter identity. This design minimized systematic bias due to litter effects. However, as all pups from a given litter shared the same prenatal treatment (Control or VPA), we acknowledge that future studies could explicitly include litter as a random effect to account for any remaining shared variance. Nevertheless, because most rats in the control group were purchased, litter effects are not expected.

Full model specifications are now detailed in the Methods section (see pages 47-51, lines 1102–1200). As a result of the analytical suggestions made by you and the other reviewers, the entire Results section has been thoroughly revised and recast.

3. Given the substantial number of neurons recorded, this study has very high statistical power, meaning that even small, non-physiological differences may reach statistical significance. Therefore, it is crucial to report the magnitude of the differences in the main body of the text, rather than just the p-values. Furthermore, no adjustments appear to have been made for the large number of comparisons conducted, nor for the fact that many neurons were used across

multiple comparisons. The authors should either adjust for multiple comparisons or provide a clear justification for why this was not done.

OUR RESPONSE:
Thank you very much for this suggestion. We agree with the recommendation and have accordingly implemented these details throughout the Results section.

Minor Concerns:

Line 47 - replace "if" with "when."

OUR RESPONSE:
Done

Tables 2-4 contain text that is cut off (e.g., "Female Cont-"). Formatting should be checked to ensure clarity.

OUR RESPONSE:
We apologize for the oversight and thank you for your observation. We have revised the tables accordingly, and everything has now been carefully checked to ensure that no cut-off issues remain.

Reviewer #2: In this study, Cacciato-Salcedo et al., examine contextual sound processing and predictive coding in a VPA-induced rat model of autism. To accomplish this, they perform single unit recordings from the lemniscal and non-lemniscal inferior colliculus (IC) of urethane-anesthetized male and female rats at two age points (prepubertal and adult) that either did or didn't received in utero exposure to VPA. There are several strengths to this study. Examination of predictive coding across development in a neurodevelopmental model is a laudable goal. The experimental techniques are generally appropriate and well-explained (though there are some questions about the statistical approach for such a complex dataset). It uses a well-established paradigm for examining contextual sound processing (deviant detection in auditory oddball paradigm), with appropriate controls (cascading standards). While there are some limitations regarding the VPA model (as there are with any model of complex neuropsychiatric disorders), this is a generally well-accepted environmental model of autism. It seriously considers the effects of key biological variables, such as age and sex, as well several experimental variables (sound intensity, lemniscal v. nonlemniscal recordings). However, there were some major concerns regarding the narrative structure and methodological approaches that limited enthusiasm.

OUR RESPONSE:
Thank you very much for your supportive comments and appreciation of our work. As we mention to reviewer#1, we agree that the data is overwhelming, and we hope that the new analysis and presentation now simplify and enhance readability.

Major Concerns

(1) The effort to consider so many biological variables (age, sex, lemniscal/non-lemniscal) is admirable, but the way the results are presented makes it extremely hard to follow, with essentially a laundry list of each possible comparison. I am sure there are some interesting results in this dataset, but it's very hard to discern what the take home points are in its current form. It is admittedly a complex dataset, so I am not sure what the best approach here is. One simple thing that would be helpful is to flip x and y axis on all the violin plots, as it feels more intuitive for most metrics (e.g. firing rate, response magnitude) to be reflected in changes on the y axis rather than x (I found myself rotating the pages to better look at the data). But the larger issue is the need to for a better narrative that highlights the differences that are most important. Perhaps by organizing the figures/results by comparisons made (e.g. effect of age, effect of sex, effect of VPA) rather than by measurements made (spontaneous activity, evoked, mismatch indices)? This way, the focus would be on the most salient group comparisons rather than attempting to compare all 8 groups for each analysis. As it stands, the results are borderline uninterpretable, which makes it difficult to evaluate their impact. While the discussion section was detailed, it did not provide much clarity in this regard. And some of the connections drawn felt fairly tenuous, e.g. not sure what conclusions can be made from comparing single unit spontaneous rates in auditory midbrain of anesthetized rats to cortical functional connectivity from resting state fMRI in awake humans (lines 570-575) or habituation dynamics in IC evoked responses to context-discrimination behavior in pavlovian conditioning tasks (lines 588-596).

OUR RESPONSE:
We agree that the presentation and interpretation of the data may have felt overwhelming. This was indeed the case, even for us as authors, who are deeply familiar with the dataset, as the complexity posed challenges in terms of clarity and flow. Recognizing this, we have taken steps to streamline the presentation and clarify key findings. Additionally, because major issues# 1 and #2 both relate to the structure and interpretation of the data, we have addressed them together in our response to issue# 2, in order to provide a more coherent and integrated explanation. Your suggestions have been extremely helpful in this process, and we are deeply grateful for your thoughtful feedback.

We have reoriented the violin plots, as we agree that this significantly improves their visual clarity. Additionally, we included a population-level analysis to highlight the main effects of sex, age, and exposure within each division and sound level. These analyses are now presented in Figures 3 and 5.

Additionally, as part of a thorough revision process, we have made substantial changes to the Discussion section, incorporating and commenting on the reviewer's suggestions throughout.

(2) There are some key methodological issues that need to be addressed. First, there needs to be a more thorough description of the statistical approach, as this is a complicated dataset with many biological variables. The authors mostly present pairwise comparison without any tests for main effects and interactions between them. This is likely because they used non-parametric tests, and non-parametric modeling solutions for multivariate statistics are more difficult. Ideally, they should also run either wilcoxon (as they did on the pairwise data) or kruskal-Wallis test (extension of the wilcoxon test that deals with multiple groups) on the main variables and not just the pairwise comparisons. They do state that they are correcting for multiple comparisons, though are one of the least stringent approaches. Perhaps most importantly, it appears that all statistical comparisons are done at the level of the unit, not subject. This is not appropriate, since the major experimental variables (sex, age, +/- VPA exposure) all act the whole animal level, individual cells/units from the same animal will have shared variance and thus cannot be considered independent samples. The statistical comparisons for these experiments should be done at the animal level not the cell/unit level. Units either need to be averaged across animals or, alternatively, a mixed model approach can be used that incorporates animal ID as a fixed variable. Consultation with a biostatistician could improve statistical analysis and perhaps find more precise and interesting ways to explore the interaction between the many experimental variables. A more conservative statistical approach might also provide some clarity to the results by highlighting the most robust effects to be focused on.

OUR RESPONSE:

We thank the reviewer for their constructive comments. After consultation with colleagues from the Department of Statistics, we have reanalyzed the data using a generalized linear mixed-effects model to better capture the complexity of our experimental design.

We agree with Reviewer #2's suggestion and, following this recommendation, we consulted a colleague from the Department of Statistics and conducted a new analysis using a generalized linear mixed-effects model. As explained to reviewer#1, this updated approach offers additional insights into between-

individual variability and reinforces our original conclusions, thereby enhancing the robustness of the analysis. We believe the revised findings provide a clearer understanding of the phenomenon and effectively address the concerns raised.

Given the complexity of our design, spanning multiple biological factors (Division, Level, Sex, Age, Exposure) and repeated measures, we selected the generalized linear mixed-effects model as the primary analytical framework. This model effectively captured both fixed effects and random effects associated with SubjectID. Where appropriate, we incorporated random slopes to account for subject-specific variability across Division, Exposure, and Level, acknowledging that both spontaneous and evoked responses varied systematically with these factors.

This analytical strategy enabled us to generate interpretations that are both statistically rigorous and biologically meaningful while appropriately modelling the hierarchical structure of the data. Specifically, we grouped neurons by Exposure, Division, Level, and SubjectID to account for potential variability in neuronal responses among individual rats. Model fit and predictor evaluation indicated that variability was primarily explained by the fixed effects rather than by within-animal differences, thus justifying the pooling of data by experimental group and neuronal level.

Full model specifications are now detailed in the Methods section (see pages 47-51, lines 1102–1200). As a result of the analytical suggestions made by Reviewer #2 and the other reviewers, the entire Results section has been thoroughly revised and recast.

In response to the reviewers' suggestions, the entire Results section has been thoroughly revised and recast. First, we present the model outcomes, followed by a population-level comparison across each division and sound level. Finally, we perform an experimental group-level analysis to identify potential specific or isolated effects at the group level. These results are now shown in a revised version of Figure 2 (spontaneous activity) and two new figures (Figure 3 and Figure 5), which more clearly illustrate the effects of Sex, Age, and Exposure across Division and Level for elicited responses and predictive components, respectively.

Finally, to complement the hierarchical analysis, we retained the experimental group-level comparisons. This additional step allowed us to confirm the general trends identified and to detect isolated differences specific to particular experimental groups. Importantly, the figures presenting group-level comparisons (Figures 4 and 6) have been simplified to enhance readability. For example, this analysis revealed that, contrary to the overall trend, adult females

prenatally exposed to VPA exhibited an increase in iMM relative to their control counterparts.

Finally, it appears that the control animal group contains a mix of those bred at their facilities and purchased from vendors (at different ages), while the VPA group were all necessarily bred in house. This creates a potential confound, as there are many important rearing variables that cannot be controlled for, such as ambient noise levels in the facilities, diet, housing conditions, etc. These issues feel particularly important for studying a neurodevelopmental disorder across multiple age points. The authors state at the end of the methods section that no statistical difference was detected in control group but they do not show any of the data or statistical comparisons. At the very least, the authors should provide a breakdown of how many animals were purchased vs bred inhouse and show the data comparing response properties between these groups. It was also unclear if the experiments were performed in an interleaved fashion. If recordings were not made from VPA/control pairs yolked for age and sex, then the between group comparisons may not be valid.

OUR RESPONSE:

We evaluated potential differences within our control group by comparing prenatally saline-injected and commercially purchased control rats across each auditory division and sound level. Saline was administered via intraperitoneal injection during pregnancy to account for any potential stress effects associated with the injection procedure, thereby ensuring that postnatal outcomes observed in the VPA group could be attributed specifically to prenatal VPA exposure.

Due to space limitations in our animal care facility, which prioritized the housing of VPA-exposed litters, the control group was partially composed of purchased animals. Among the control group, the saline-injected offspring included three animals in the Control Prepubertal Female group, two in the Control Prepubertal Male group, two in the Control Adult Male group, and one in the Control Adult Female group.

To confirm the absence of statistical differences between groups matched by sex and age, we compared spontaneous and evoked neuronal responses separately within each division and sound level, using Wilcoxon rank-sum tests with Bonferroni correction. These analyses did not reveal any significant differences between injected and purchased animals within each sex and age group. Based on these results, we compiled all control animals into a single, unified group for subsequent analyses.

Given that the manuscript already contains a substantial amount of tables and figures, we believe it is not necessary to include these additional data. However, if the reviewer considers it important, we would be happy to incorporate them.

Other concerns:

Abstract/Intro

Line 47: typo? (should it read "In comparing control.." instead of If?)

OUR RESPONSE:
We changed as suggested by reviewer#1

Line 47-49 wording is confusing, is the point trying to be made that there was a reduction in neuronal mismatch in all VPA rats except the adult females, which instead exhibited an increase?

OUR RESPONSE:

We apologize for the earlier unclear wording. We have now revised the text to make it clearer.

Figure 1A is never referenced in text

OUR RESPONSE:
Thanks for this observation. It is cited now

Methods/Results

While inclusion of two intensities is admirable, not sure how informative it is given the vagueness of absolute intensity and range across the population of units recorded.

OUR RESPONSE:

We apologize if our previous explanation was not sufficiently clear. We have previously demonstrated that, particularly within the inferior colliculus (IC), differences in sound level significantly affect the processing of iMM and iPE (Parras et al., 2017; Carbajal et al., 2024), with the strongest effects observed in the non-lemniscal division. Therefore, we consider sound level to be a critical variable in our analysis.

Indeed, one of the main findings of the present study is that iPE becomes positive in adult animals while remaining negative in prepubertal animals within the non-lemniscal division under low-intensity stimulation. This finding suggests a possible postnatal development of the iPE. We hope that the newly added Figure 5 clearly illustrates this result across each sound level.

Line 940: What was the reasoning for using only non-parametric tests? Was data tested for normality?

Would be nice to see some example PTSHs to std, dev, and control stimuli
Some p values reported seemed to be exact values but still used < rather than = (e.g. line 186 $p < 0.0404$)

OUR RESPONSE:

The reviewer is correct; the data distribution was not normal, which is why we selected the generalized linear mixed-effects model. This approach does not assume normality of the data, making it more appropriate for our analysis.

Yes, we have now included some exemplary PSTHs in Figure 1F.

Thank you for your comment regarding the p-values; we have corrected them throughout the manuscript.

Line 132-137: Would be best to report n for rats as well as units, e.g. n = 102 units from XX rats

OUR RESPONSE:
We have now included these ‘n’ as requested.

Line 205-206 (Fig 2): Spontaneous rates in adult non-meniscal VPA looks to be bimodally distributed. Could this be explained by individual animal variations?

OUR RESPONSE:
The model we implemented for spontaneous activity confirms that inter-individual variability is explained by the experimental variables of sex, age, exposure, division, and sound level. Thus, the apparent variation may be a visual effect resulting from the smoothing process.

Line 537: typo? "When age is considered, females probe a major decrease…"

OUR RESPONSE:
As previously, this paragraph has been entirely revised.

Discussion

The discussion section is rather long, with the first several paragraphs essentially just restating the results without much context or synthesis. The second half (after line 647, the "Precision weighing accounts…" subheading) does a better job of this and helps put a spotlight on some of the more interesting findings. The initial summary of the results should be shorten with more focus on synthesis and highlighting most important results.
The authors try to make the case that altered spontaneous rates in VPA rats (although oinly observed in specific nuclei at specific ages) relates to altered predictive processing. Was there any correlation between spontaneous rates and deviant indices on a unit-to-unit or subject-to-subject basis?
There was surprisingly little discussion of how the current results compare to a recent study (Valerio, et al, Sci Advances 2024) that examined the development of stimulus specific adaption across the auditory neuraxis in mice, including IC. That study suggests stimulus specific adaption is done maturing in the IC by prepuberty, contrary to the findings here. This previous study is cited, but there is no discussion of how its results compare to the results of the current study.
Intriguingly, the authors did not find any difference in true deviant detection (iPE) with valproic acid, this feels like it should be highlighted more in the discussion.

OUR RESPONSE:

Thank you very much for your helpful feedback. We agree that the previous version of the Discussion was somewhat verbose and occasionally redundant. In response, we have made a concerted effort to reduce its length and sharpen the focus on the functional context of our results. The original Discussion was 2,077 words and has now been shortened to 1,937. While the overall reduction may appear modest, we have also incorporated new content, as you suggested. These additions include: (1) a discussion of the relationship between spontaneous activity and mismatch responses (see pages 29-30, lines 666-707); (2) a more detailed comparison with the elegant study by Valerio and colleagues (see page 31, lines 718-735); (3) a new section on limitations and future directions, as also recommended by Reviewer #3 and the editor (see pages 35-36, lines 809-835); and (4) a discussion on true deviance detection (iPE). We also appreciate your attention to this omission regarding iPE and the lack of differences observed with valproic acid exposure (see updated Figure 9; see pages 32-33, lines 747-776).

Overall, we believe the Discussion has been significantly streamlined while offering a clearer and more functional interpretation of our findings.

General
The structuring of paragraphs felt uneven, in some cases there were very short paragraphs (1-2 sentences) that seemed to be clearly connected to each other and could be combined. In other cases, there were very long paragraphs (> 1 pg) that would probably be better off broken up.

OUR RESPONSE:

We have made a concerted effort to improve the structure of the paragraphs.

Reviewer #3: The manuscript, "Atypical Predictive Coding in the Inferior Colliculus of the Valproic Acid-Induced Rat Model of Autism: Sex, Age, and Exposure Differences" by Cacciato-Salcedo et al., investigates how neurons in the inferior colliculus (IC) respond differently based on sex, age, and in an autism animal model. Using both prepubertal and adult male and female rats, the authors performed electrophysiological recordings in the IC of anesthetized animals. Their analyses examine differences in spontaneous activity, evoked responses to contextual sounds, neuronal mismatch components, and adaptation across sex, age, and valproic acid (VPA) exposure groups. The experiments are well-structured, and the group comparisons are clearly presented. However, the study's broader significance is not fully articulated, particularly in terms of its relevance to autism-related behaviors. Specific concerns are outlined below.

OUR RESPONSE:

Thank you very much for your supportive comments and appreciation of our work. We hope that the new analysis and presentation now simplify and enhance readability.

Major

1. There is no assessment of how these electrophysiological differences relate to behavioral outcomes, including both autism-related and non-related behaviors, across sex, age, and exposure groups.

2. Recordings were performed in anesthetized rats, it further raises the concern on how these findings link to behaviors.

OUR RESPONSE:

We address major issues 1 and 2 together, as they are closely related. The reviewer is absolutely right in identifying these limitations, and we fully acknowledge their importance. As also suggested by the editor, we have added a dedicated paragraph in the Discussion section to elaborate on these constraints. Conducting experiments in awake, behaving animals would represent a significant extension of the current study; one that would require a separate and extensive project, likely spanning an additional 2–3 years solely for the experimental work.

We respectfully submit that the current dataset offers valuable insights and is sufficiently robust to support the conclusions presented, thereby justifying its consideration for publication. We are confident that it lays a strong foundation for future investigations. These limitations are now thoroughly addressed in the revised Discussion section***. Please see pages 35-36, lines 809-835.***

3. It is not clear whether single units included in the analyses are all excitatory or combined with inhibitory units. If it is combined, it would be interesting to reveal whether excitatory and inhibitory units display differently in these analyses.

OUR RESPONSE:

This is a very interesting suggestion. Indeed, neurons in the cortex can be classified as excitatory or inhibitory based on the shape of their action potentials. Unfortunately, to the best of our knowledge, this is not feasible in the inferior colliculus. Therefore, unless we conduct intracellular recordings, we cannot distinguish between excitatory and inhibitory neurons. However, intracellular recordings in vivo in the IC are not only extremely time-consuming but also technically challenging, making it nearly impossible to gather a sufficient sample to draw reliable conclusions. For example, in a previous study (Valdes-Baizabal et al., 2020), we were only able to collect data from 13 neurons over the course of four years. Again, this limitation is discussed in the manuscript (see pages 35-36, lines 809-835), as it complements the previous point regarding experimental feasibility.

4. Some key details are missing from the Results and Methods sections. For instance, the explanation of the power-law model lacks sufficient clarity. The authors should elaborate on the biological relevance of power-law adaptation and define key variables such as y and t, as well as the meaning of "averaged spikes". Additionally, the statement, "These results suggest that prenatal VPA

exposure alters the processing of stimulus statistics over multiple time scales in the IC," is vague, as "stimulus statistics" is not clearly defined. Providing a more explicit explanation would strengthen the interpretation of the results. Furthermore, the calculation of the half-adaptation measure is not clearly described, making it difficult to assess its significance.

OUR RESPONSE:

Thank you for this suggestion. We have now added an additional explanation in the Methods section and, where appropriate, throughout the manuscript. Also, the sentence related to "stimulus statistics" has been clarified, as we agree it was too vague. ***Please see Methods: page 51, lines 1201-1210; and see Results: pages 26-27, lines 611-615.***

Minor:

1. Neuron numbers were provided in the text of the result section, but with no information on associated animal numbers.

OUR RESPONSE:

Thanks and sorry, this is now implemented as also suggested by reviewer#2

2. Figure 2 right panel, there are 4 smaller plots next to the schematic drawing. Not sure what they are (no legend for them). Same for Figure 3A&4A.

OUR RESPONSE:

We apologize for the confusion regarding the panels; however, these figures have since been revised and/or removed.

3. Figure 3, CTR was used in figure panels, but CAS was used in the legend.

OUR RESPONSE:

Sorry for the typo, fixed

4. Figure 6, what is "TD" groups? No information in the legend and text.

OUR RESPONSE:

We apologize for the confusion; it was originally meant to indicate "Typical development." However, due to the figure revisions, we have now updated the abbreviations throughout all figures and the manuscript.
