# Supplementary material for: Contextual auditory processing in the inferior colliculus is affected in a sex- and age-dependent manner in the valproic acid-induced rat model of autism
Source: PLoS Biol. 2025 Aug 4;23(8):e3003309. doi: 10.1371/journal.pbio.3003309 (PMC12342314; doi:10.1371/journal.pbio.3003309)
Supplement: Attachment — Submitted filename: Response to reviewers.docx [file pbio.3003309.s012.docx]

Dear Manuel,

Thank you for your patience while we considered your revised manuscript "Atypical predictive coding in the inferior colliculus of the valproic acid-induced rat model of autism: Sex, age and exposure differences" for publication as a Research Article at PLOS Biology. This revised version of your manuscript has been evaluated by the PLOS Biology editors, the Academic Editor and the original reviewers.

Based on the reviews and on our Academic Editor's assessment of your revision, we are likely to accept this manuscript for publication, provided you satisfactorily address the remaining points raised by the reviewers. To address Reviewer 3 concerns, we encourage you to add a discussion about these concerns in the Limitations section of your manuscript.


Please also make sure to address the following data and other policy-related requests.

* We would like to suggest a different title to improve its accessibility for our broad audience:
Contextual auditory processing in the inferior colliculus is affected in a sex- and age-dependent manner in the valproic-acid-induced rat model of autism

OUR RESPONSE:

Thank you very much for your supportive comments and for the opportunity to submit an additional revision to improve our manuscript.
We have changed the title as suggested and carefully considered all reviewer comments. The manuscript has been revised accordingly, including the addition of a new paragraph in the *Discussion* to specifically address the issues raised by Reviewer #3.
We sincerely hope that this extensively revised version will now be favorably considered for acceptance and publication.

Thank you once again to the reviewers, the Academic Editor, and yourself for your time, thoughtful feedback, and support throughout the review process.

Reviewer remarks:

Reviewer #1: I have reviewed the revised manuscript. The authors have made substantial improvements to the manuscript: they have added a hierarchical analysis which was very much needed, they have discussed more thoroughly the weaknesses in the manuscript regarding the lack of formal behavioral assessments and the use of anaesthetised animals, they have re-oriented the violin plots and now provide numbers of rats as well as units. All of these changes have added substantial clarity to what was already a very important manuscript. I have no further suggestions for this manuscript.

OUR RESPONSE:

Thank you very much for your appreciation of our revised manuscript. We sincerely value all your insightful suggestions, which have contributed significantly to improving the quality of the work.

Reviewer #2: This revised manuscript is much improved, and the authors have put considerable effort into addressing the main reviewer concerns of statistical methodology and narrative structure. The revised manuscript performs a much more thorough and rigorous statistical analysis and completely reworks the results section and figures to first highlight population and group level comparison before delving into all the potential interactions. The discussion section also does a better job of putting results in bigger picture context, though there are a few points that could be expanded on or made clearer. While it is still a dense read, the authors did a commendable job in making the findings from this study more interpretable give the challenging and complex nature of the data set. The overarching conclusion is somewhat unsatisfying (that age, sex, and VPA exposure effect contextual sound processing in the IC in a complex and interconnected manner), but there are some
specific take home points that this reviewer found to be interesting and informative, if somewhat counterintuitive.

OUR RESPONSE:

Thank you very much for your appreciation of our revised manuscript. We are sincerely grateful for your insightful suggestions, which have significantly improved the clarity and interpretation of our data.

We also appreciate the reviewer’s earlier analytical recommendations, which substantially enhanced the readability and depth of our findings. We fully agree that psychiatric conditions, including neurodevelopmental disorders such as autism, are profoundly influenced by interactive biological, environmental, cultural, and social factors intrinsic to the human experience (Schmengler et al., 2021; Araujo Scattolin et al., 2022). We share the reviewer’s view that basic research using mammalian models should continue to build upon insights from human studies, aiming to examine biological variables and their interactions with increasing nuance and integration.

By embracing the complexity of these interacting variables, situated within an inherently dynamic and relational world, we may move closer to understanding the heterogeneity that defines neurodevelopmental disorders across the lifespan.

Major comments
The authors did a commendable job in re-working the figures. One further suggestion to improve interpretability would be to consistently label all axis, violin plots (FpC, FaC, MpC…), and color-coded groups (CAS/DEV/STD or iMM/iRS/iPE). Because there are so many groups/variables that need to be kept straight, having labels on each subplot (while perhaps not the most aesthetically pleasing choice) would greatly facilitate interpretation of the graphs.

OUR RESPONSE:

Thanks for the suggestion, we have made the changes as requested.

In terms of the effect of VPA exposure and autism, the major finding from this study was that VPA exposure reduced mismatch processing, with an in increase iRS but reduced iPE (at least for high sound levels in the non-lemniscal IC). The authors' interpret these results in the context of hypo-priorism in ASD, as attenuated priors would be expected to produce less robust prediction errors. This makes sense, but the logic as to how reduced priors would lead to greater iRS was unclear to me, as one may expect reduced rather than enhanced habituation if sensory input is treated as less predictable.  Indeed, previous results in other ASD models have found impaired adaptation to repetitive sensory input (perhaps most notably in Fragile X Syndrome, see Ethridge et al., 2016; Lovelace et al., 2016; He et al., 2017). How can these results be reconciled?

OUR RESPONSE:

Thank you for this comment, which prompted us to revisit this section and recognize that we had misunderstood the underlying concept.

We apologize if we misinterpreted the concept of hypo-priorism in autism; the reviewer is correct in their interpretation. Clearly, the notion of hyper-priors provides a more accurate framework for explaining our population-level findings. Within this framework, overly precise internal predictions suppress neural responses to novel stimuli, thereby weakening mismatch responses and impairing model updating. Excessively rigid priors generate smaller prediction errors because the brain underestimates discrepancies between expected and actual sensory input. This leads to a shift in processing toward a stronger reliance on internal predictive models, as reflected in enhanced iRS. Enhanced repetition suppression naturally arises as repeated stimuli robustly confirm these rigid predictions. Ultimately, this imbalance between top-down and bottom-up processing manifests as atypical mismatch responses, observable through electrophysiological measures. We have clarified this interpretation in the revised *Discussion* section (see lines 748–757).

For further clarification, we have now included a female-specific subsection in the *Discussion, lines 789-796*, and expanded our explanation of findings in male and prepubertal female groups. These findings support the existing literature on reduced repetition suppression and mismatch negativity in autism and other neurodevelopmental models, including fragile X síndrome, patterns that are more consistent with hypo-priorism. Please refer to lines 780–783 in the *Discussion* section.

Perhaps the most interesting finding from this study is that VPA exposure causes female specific deficits in IC development and contextual sound processing in the IC. The authors provide some context for these findings in terms of how they relate to other results from the VPA model, but this is an interesting finding in light of male prevalence of ASD and should be discussed more explicitly.

OUR RESPONSE:

We agree with the reviewer. We created a female section in the Discussion to further expand our results with existing scientific literature. See *lines 789-796*,

Minor comments
Line 50 typo "well-stablished"

OUR RESPONSE:

Thanks!!! corrected

Line117-143 This paragraph is rather long and should probably be split into two. Sentence starting with "We performed single-unit recordings.." (line 129) seems like a natural place for this.

OUR RESPONSE:

Thanks!!! corrected

Line 135-136: "In utero administration of VPA in pregnant rats induced autism-like signs in the offspring." Not sure if enough data is provided to support this claim.

OUR RESPONSE:

We revised the text accordingly and added “neuronal signatures” to clarify that no behavioral analyses were conducted.

Line 165 cite figure 1D at end of sentence

OUR RESPONSE:

Thanks!!! corrected

Line 193/255/405: typo, "We fitted" should be "We fit"

OUR RESPONSE:

Thanks!!! We have corrected throughout

Lines 213-222: Various words are used in this section to describe spontaneous rates (scores, values, responses), making it somewhat confusing. Sticking with spontaneous rates or spontaneous activity levels would make this section clearer.

OUR RESPONSE:

Thanks!!! Agree and corrected

Line 282 p value typo (says significant sex x age x exposure but p = 0.14)

OUR RESPONSE:

Agree and corrected, it should be 0.014, thanks!!!!

Line 339-340 "Among adult females, VPA exposure reduced DEV response compared to controls in the lemniscal division at high-level stimulation" This doesn't seem to match the data in Figure 4 lemniscal, high level plot, where FaV distribution appears to be higher than FaC.

OUR RESPONSE:

Agree and corrected, thanks!!!!

Fig 7: It wasn't clear why only subset of conditions are looked at here. Was this due to inadequate power law fits for these data sets? May be worth showing an example of response that doesn't follow power law adaptation (e.g. for VPA groups).

OUR RESPONSE:
Thank you for the suggestion. However, we respectfully disagree, as the figure is already visually dense, and adding this negative panel would increase its complexity and make it even more crowded.
Nevertheless, to address this point, we have added a sentence to clarify the explanation and improve the flow of the narrative. Please see line 615-616.

Fig 8: Not sure I agree with the interpretation of these results, seems like it's more of a difference in starting point than in adaptation rate. Is there a way to quantify this (e.g. curve fitting or response ratio)?

OUR RESPONSE:

We apologize for the lack of clarity in our original description, which may have led to a misinterpretation, as appears to be the case in the reviewer’s comment. This procedure has been implemented in previous publications (e.g., Nieto-Diego and Malmierca, 2016; Parras et al., 2020) from our lab and is therefore well standardized. We have now properly cited the relevant reference in the methods (please see line XXX)

Line 689 typo: "impaired maturation of high-spontaneous-rate in the auditory nerve fibers"

OUR RESPONSE:

Agree and corrected, thanks!!!!

Lines 718-735. In reference to Valerio et al., the authors state that this study found that neuronal mismatch is already mature in lemniscal IC by prepurberty (lines 719-720) but then state that this study found a decrease in neuronal mismatch with age up to P50 in lemniscal IC (724-725). These statements seem contradictory, unless I am misinterpreting something.

OUR RESPONSE:

We agree that the narrative was confusing and disorganized. We have revised the description to clarify the statement and have separated it into two paragraphs to improve readability and flow. Please see lines XXX

Line 759-761 "our single-unit findings, together with behavioral evidence, reveal a mechanistic link between atypical predictive processing and core sensory and behavioral features of autism." This feels like too strong of a statement, given no behavioral phenotyping was done and no direct correlation between altered mismatch processing and repetitive behaviors was performed.
OUR RESPONSE:

Thank you again!!! we have revised the text to tone down the original statement.

Reviewer #3: I acknowledge the technical issues in response to my last review comments. However, my concern regarding the significance of the study remains. It is still unclear which of the reported electrophysiological differences are specifically related to autism, and which may simply reflect characteristics of the animal model that are unrelated to autism-like behaviors.

OUR RESPONSE:

We thank the reviewer for raising this important issue. As acknowledged, animal models inherently carry limitations when attempting to disentangle construct-specific effects from broader phenotypic variation. Our study did not claim to isolate electrophysiological markers specific to autism, but rather to identify neural signatures that support theoretical accounts of the condition, particularly predictive coding models. The observed differences in predictive components of neuronal mismatch are interpreted within this framework and contextualized using comparable findings from both human studies and other neurodevelopmental models (e.g., fragile X syndrome).

To specifically address the reviewer’s concern, we have clarified this point in the revised *Discussion*. While the VPA model recapitulates certain autism-relevant traits, the electrophysiological alterations observed may also reflect broader neurodevelopmental disruptions. We further emphasize that our goal is to provide mechanistic insight into auditory processing abnormalities relevant to autism, while acknowledging the translational limitations of the model. We hope that these clarifications adequately address the reviewer’s concern.
