# Supplementary material for: Contextual auditory processing in the inferior colliculus is affected in a sex- and age-dependent manner in the valproic acid-induced rat model of autism
Source: PLoS Biol. 2025 Aug 4;23(8):e3003309. doi: 10.1371/journal.pbio.3003309 (PMC12342314; doi:10.1371/journal.pbio.3003309)
Supplement: Peer review history

# Editor report: Decision Letter 0

**DOI:** 10.1371/journal.pbio.3003309.r001

**Authors:** Christian Schnell, PhD

19 Jan 2025

Dear Manolo,

Thank you for submitting your manuscript entitled "Atypical predictive coding in the inferior colliculus of the valproic acid-induced rat model of autism:  Sex, age and exposure differences" for consideration as a Research Article by PLOS Biology.

Your manuscript has now been evaluated by the PLOS Biology editorial staff as well as by an academic editor with relevant expertise and I am writing to let you know that we would like to send your submission out for external peer review.

However, before we can send your manuscript to reviewers, we need you to complete your submission by providing the metadata that is required for full assessment. To this end, please login to Editorial Manager where you will find the paper in the 'Submissions Needing Revisions' folder on your homepage. Please click 'Revise Submission' from the Action Links and complete all additional questions in the submission questionnaire.

Once your full submission is complete, your paper will undergo a series of checks in preparation for peer review. After your manuscript has passed the checks it will be sent out for review. To provide the metadata for your submission, please Login to Editorial Manager (https://www.editorialmanager.com/pbiology) within two working days, i.e. by Jan 21 2025 11:59PM.

If your manuscript has been previously peer-reviewed at another journal, PLOS Biology is willing to work with those reviews in order to avoid re-starting the process. Submission of the previous reviews is entirely optional and our ability to use them effectively will depend on the willingness of the previous journal to confirm the content of the reports and share the reviewer identities. Please note that we reserve the right to invite additional reviewers if we consider that additional/independent reviewers are needed, although we aim to avoid this as far as possible. In our experience, working with previous reviews does save time.

If you would like us to consider previous reviewer reports, please edit your cover letter to let us know and include the name of the journal where the work was previously considered and the manuscript ID it was given. In addition, please upload a response to the reviews as a 'Prior Peer Review' file type, which should include the reports in full and a point-by-point reply detailing how you have or plan to address the reviewers' concerns.

During the process of completing your manuscript submission, you will be invited to opt-in to posting your pre-review manuscript as a bioRxiv preprint. Visit http://journals.plos.org/plosbiology/s/preprints for full details. If you consent to posting your current manuscript as a preprint, please upload a single Preprint PDF.

Feel free to email us at plosbiology@plos.org if you have any queries relating to your submission.

Kind regards,

Christian

Christian Schnell, PhD

Senior Editor

PLOS Biology

cschnell@plos.org

# Peer review: Decision Letter 1

**DOI:** 10.1371/journal.pbio.3003309.r002

**Authors:** Christian Schnell, PhD

12 Mar 2025

Dear Manolo,

Thank you for your patience while your manuscript "Atypical predictive coding in the inferior colliculus of the valproic acid-induced rat model of autism:  Sex, age and exposure differences" was peer-reviewed at PLOS Biology. It has now been evaluated by the PLOS Biology editors, an Academic Editor with relevant expertise, and by several independent reviewers.

In light of the reviews, which you will find at the end of this email, we would like to invite you to revise the work to thoroughly address the reviewers' reports.

As you will see below, the reviewers agree that the topic is interesting and the findings potentially important, but they mention a lot of concerns regarding the statistical analyses, the overall presentation, lack of validation of the animal model, lack of behavioral assessment and the fact that experiments have been conducted in anesthetized animals.

We encourage you to address all reviewer concerns, but understand that the concerns regarding the lack of behavioral data and the anesthesia may be very challenging to address. If you are not able to provide additional experimental data to address these points, please discuss both as limitations in the Discussion section.

Given the extent of revision needed, we cannot make a decision about publication until we have seen the revised manuscript and your response to the reviewers' comments. Your revised manuscript is likely to be sent for further evaluation by all or a subset of the reviewers.

In addition to these revisions, you will need to complete some formatting changes, which you will receive in a follow up email. A member of our team will be in touch with a set of requests shortly.

We expect to receive your revised manuscript within 3 months. Please email us (plosbiology@plos.org) if you have any questions or concerns, or would like to request an extension.

At this stage, your manuscript remains formally under active consideration at our journal; please notify us by email if you do not intend to submit a revision so that we may withdraw it.

**IMPORTANT - SUBMITTING YOUR REVISION**

Your revisions should address the specific points made by each reviewer. Please submit the following files along with your revised manuscript:

1. A 'Response to Reviewers' file - this should detail your responses to the editorial requests, present a point-by-point response to all of the reviewers' comments, and indicate the changes made to the manuscript.

*NOTE: In your point-by-point response to the reviewers, please provide the full context of each review. Do not selectively quote paragraphs or sentences to reply to. The entire set of reviewer comments should be present in full and each specific point should be responded to individually, point by point.

You should also cite any additional relevant literature that has been published since the original submission and mention any additional citations in your response.

2. In addition to a clean copy of the manuscript, please also upload a 'track-changes' version of your manuscript that specifies the edits made. This should be uploaded as a "Revised Article with Changes Highlighted" file type.

*Re-submission Checklist*

When you are ready to resubmit your revised manuscript, please refer to this re-submission checklist: https://plos.io/Biology_Checklist

To submit a revised version of your manuscript, please go to https://www.editorialmanager.com/pbiology/ and log in as an Author. Click the link labelled 'Submissions Needing Revision' where you will find your submission record.

Please make sure to read the following important policies and guidelines while preparing your revision:

*Published Peer Review*

Please note while forming your response, if your article is accepted, you may have the opportunity to make the peer review history publicly available. The record will include editor decision letters (with reviews) and your responses to reviewer comments. If eligible, we will contact you to opt in or out. Please see here for more details:

https://blogs.plos.org/plos/2019/05/plos-journals-now-open-for-published-peer-review/

*PLOS Data Policy*

Please note that as a condition of publication PLOS' data policy (http://journals.plos.org/plosbiology/s/data-availability) requires that you make available all data used to draw the conclusions arrived at in your manuscript. If you have not already done so, you must include any data used in your manuscript either in appropriate repositories, within the body of the manuscript, or as supporting information (N.B. this includes any numerical values that were used to generate graphs, histograms etc.). For an example see here: http://www.plosbiology.org/article/info%3Adoi%2F10.1371%2Fjournal.pbio.1001908#s5

*Blot and Gel Data Policy*

We require the original, uncropped and minimally adjusted images supporting all blot and gel results reported in an article's figures or Supporting Information files. We will require these files before a manuscript can be accepted so please prepare them now, if you have not already uploaded them. Please carefully read our guidelines for how to prepare and upload this data: https://journals.plos.org/plosbiology/s/figures#loc-blot-and-gel-reporting-requirements

*Protocols deposition*

To enhance the reproducibility of your results, we recommend that if applicable you deposit your laboratory protocols in protocols.io, where a protocol can be assigned its own identifier (DOI) such that it can be cited independently in the future. Additionally, PLOS ONE offers an option for publishing peer-reviewed Lab Protocol articles, which describe protocols hosted on protocols.io. Read more information on sharing protocols at https://plos.org/protocols?utm_medium=editorial-email&utm_source=authorletters&utm_campaign=protocols

Thank you again for your submission to our journal. We hope that our editorial process has been constructive thus far, and we welcome your feedback at any time. Please don't hesitate to contact us if you have any questions or comments.

Sincerely,

Christian

Christian Schnell, PhD

Senior Editor

PLOS Biology

cschnell@plos.org

------------------------------------

REVIEWS:

Reviewer #1: Summary:

In this study, the researchers conducted single-unit recordings in the inferior colliculus of anaesthetised animals to examine age- and sex-related variability in predictive coding in a rat model of autism. Specifically, they utilised valproic acid-induced (VPA) rat models within an oddball paradigm and non-repetitive cascade control sequences to investigate mismatch negativity. The rats were divided into multiple groups for this study: control and VPA rats, further categorised by sex and age (prepubertal and adult). The authors report several age- and sex-related differences in VPA rats, providing further evidence of the heterogeneity of autism-like symptoms and predictive coding.

Overall, the investigation of predictive coding in an autism model is highly valuable, given existing hypotheses on the subject. Additionally, the study originates from a group of researchers well known for their excellent work in predictive coding. However, several concerns limit the impact of this paper. After multiple readings, this reviewer found the data presentation overwhelming, with numerous comparisons (young vs. adult, male vs. female, loud vs. soft, VPA vs. non-VPA, lemniscal vs. non-lemniscal), making it difficult to discern any overarching conclusions. Further concerns are outlined below regarding the statistical approaches and the connection between the auditory and autistic phenotypes.

Major Concerns:

1. While VPA is known to increase the risk of autism-like symptoms in rats, how was this confirmed? The methods section mentions that minor tail malformations are present in VPA rats, but no additional checks, such as behavioural studies, are referenced. The cited study on tail malformations describes the phenotype in rats dosed with 450 mg/kg (or higher), but not at 400 mg/kg, which was the dose used in this study. It would have been beneficial to assess whether the severity of the "autism" phenotype correlates with the neurophysiological measurements.

2. In this study, recordings from individual neurons were pooled within each group (e.g., adult female VPA rats) for comparison, yet little mention is made of the variation between individual rats or litters within each group. It is essential to determine whether the main source of variability arises between litters, between individual rats, between treatment groups, or between neurons.

3. Given the substantial number of neurons recorded, this study has very high statistical power, meaning that even small, non-physiological differences may reach statistical significance. Therefore, it is crucial to report the magnitude of the differences in the main body of the text, rather than just the p-values. Furthermore, no adjustments appear to have been made for the large number of comparisons conducted, nor for the fact that many neurons were used across multiple comparisons. The authors should either adjust for multiple comparisons or provide a clear justification for why this was not done.

Minor Concerns:

Line 47 - replace "if" with "when."

Tables 2-4 contain text that is cut off (e.g., "Female Cont-"). Formatting should be checked to ensure clarity.

Reviewer #2: In this study, Cacciato-Salcedo et al., examine contextual sound processing and predictive coding in a VPA-induced rat model of autism. To accomplish this, they perform single unit recordings from the lemniscal and non-lemniscal inferior colliculus (IC) of urethane-anesthetized male and female rats at two age points (prepubertal and adult) that either did or didn't received in utero exposure to VPA. There are several strengths to this study. Examination of predictive coding across development in a neurodevelopmental model is a laudable goal. The experimental techniques are generally appropriate and well-explained (though there are some questions about the statistical approach for such a complex dataset). It uses a well-established paradigm for examining contextual sound processing (deviant detection in auditory oddball paradigm), with appropriate controls (cascading standards). While there are some limitations regarding the VPA model (as there are with any model of complex neuropsychiatric disorders), this is a generally well-accepted environmental model of autism. It seriously considers the effects of key biological variables, such as age and sex, as well several experimental variables (sound intensity, lemniscal v. nonlemniscal recordings). However, there were some major concerns regarding the narrative structure and methodological approaches that limited enthusiasm.

Major Concerns

(1) The effort to consider so many biological variables (age, sex, lemniscal/non-lemniscal) is admirable, but the way the results are presented makes it extremely hard to follow, with essentially a laundry list of each possible comparison. I am sure there are some interesting results in this dataset, but it's very hard to discern what the take home points are in its current form. It is admittedly a complex dataset, so I am not sure what the best approach here is. One simple thing that would be helpful is to flip x and y axis on all the violin plots, as it feels more intuitive for most metrics (e.g. firing rate, response magnitude) to be reflected in changes on the y axis rather than x (I found myself rotating the pages to better look at the data). But the larger issue is the need to for a better narrative that highlights the differences that are most important. Perhaps by organizing the figures/results by comparisons made (e.g. effect of age, effect of sex, effect of VPA) rather than by measurements made (spontaneous activity, evoked, mismatch indices)? This way, the focus would be on the most salient group comparisons rather than attempting to compare all 8 groups for each analysis. As it stands, the results are borderline uninterpretable, which makes it difficult to evaluate their impact. While the discussion section was detailed, it did not provide much clarity in this regard. And some of the connections drawn felt fairly tenuous, e.g. not sure what conclusions can be made from comparing single unit spontaneous rates in auditory midbrain of anesthetized rats to cortical functional connectivity from resting state fMRI in awake humans (lines 570-575) or habituation dynamics in IC evoked responses to context-discrimination behavior in pavlovian conditioning tasks (lines 588-596).

(2) There are some key methodological issues that need to be addressed.

First, there needs to be a more thorough description of the statistical approach, as this is a complicated dataset with many biological variables. The authors mostly present pairwise comparison without any tests for main effects and interactions between them. This is likely because they used non-parametric tests, and non-parametric modeling solutions for multivariate statistics are more difficult. Ideally, they should also run either wilcoxon (as they did on the pairwise data) or kruskal-Wallis test (extension of the wilcoxon test that deals with multiple groups) on the main variables and not just the pairwise comparisons. They do state that they are correcting for multiple comparisons, though are one of the least stringent approaches. Perhaps most importantly, it appears that all statistical comparisons are done at the level of the unit, not subject. This is not appropriate, since the major experimental variables (sex, age, +/- VPA exposure) all act the whole animal level, individual cells/units from the same animal will have shared variance and thus cannot be considered independent samples. The statistical comparisons for these experiments should be done at the animal level not the cell/unit level. Units either need to be averaged across animals or, alternatively, a mixed model approach can be used that incorporates animal ID as a fixed variable. Consultation with a biostatistician could improve statistical analysis and perhaps find more precise and interesting ways to explore the interaction between the many experimental variables. A more conservative statistical approach might also provide some clarity to the results by highlighting the most robust effects to be focused on.

Finally, it appears that the control animal group contains a mix of those bred at their facilities and purchased from vendors (at different ages), while the VPA group were all necessarily bred in house. This creates a potential confound, as there are many important rearing variables that cannot be controlled for, such as ambient noise levels in the facilities, diet, housing conditions, etc. These issues feel particularly important for studying a neurodevelopmental disorder across multiple age points. The authors state at the end of the methods section that no statistical difference was detected in control group but they do not show any of the data or statistical comparisons. At the very least, the authors should provide a breakdown of how many animals were purchased vs bred inhouse and show the data comparing response properties between these groups. It was also unclear if the experiments were performed in an interleaved fashion. If recordings were not made from VPA/control pairs yolked for age and sex, then the between group comparisons may not be valid.

Other concerns:

Abstract/Intro

Line 47: typo? (should it read "In comparing control.." instead of If?)

Line 47-49 wording is confusing, is the point trying to be made that there was a reduction in neuronal mismatch in all VPA rats except the adult females, which instead exhibited an increase?

Figure 1A is never referenced in text

Methods/Results

While inclusion of two intensities is admirable, not sure how informative it is given the vagueness of absolute intensity and range across the population of units recorded.

Line 940: What was the reasoning for using only non-parametric tests? Was data tested for normality?

Would be nice to see some example PTSHs to std, dev, and control stimuli

Some p values reported seemed to be exact values but still used < rather than = (e.g. line 186 p < 0.0404)

Line 132-137: Would be best to report n for rats as well as units, e.g. n = 102 units from XX rats

Line 205-206 (Fig 2): Spontaneous rates in adult non-meniscal VPA looks to be bimodally distributed. Could this be explained by individual animal variations?

Line 537: typo? "When age is considered, females probe a major decrease…"

Discussion

The discussion section is rather long, with the first several paragraphs essentially just restating the results without much context or synthesis. The second half (after line 647, the "Precision weighing accounts…" subheading) does a better job of this and helps put a spotlight on some of the more interesting findings. The initial summary of the results should be shorten with more focus on synthesis and highlighting most important results.

The authors try to make the case that altered spontaneous rates in VPA rats (although oinly observed in specific nuclei at specific ages) relates to altered predictive processing. Was there any correlation between spontaneous rates and deviant indices on a unit-to-unit or subject-to-subject basis?

There was surprisingly little discussion of how the current results compare to a recent study (Valerio, et al, Sci Advances 2024) that examined the development of stimulus specific adaption across the auditory neuraxis in mice, including IC. That study suggests stimulus specific adaption is done maturing in the IC by prepuberty, contrary to the findings here. This previous study is cited, but there is no discussion of how its results compare to the results of the current study.

Intriguingly, the authors did not find any difference in true deviant detection (iPE) with valproic acid, this feels like it should be highlighted more in the discussion.

General

The structuring of paragraphs felt uneven, in some cases there were very short paragraphs (1-2 sentences) that seemed to be clearly connected to each other and could be combined. In other cases, there were very long paragraphs (> 1 pg) that would probably be better off broken up.

Reviewer #3: The manuscript, "Atypical Predictive Coding in the Inferior Colliculus of the Valproic Acid-Induced Rat Model of Autism: Sex, Age, and Exposure Differences" by Cacciato-Salcedo et al., investigates how neurons in the inferior colliculus (IC) respond differently based on sex, age, and in an autism animal model. Using both prepubertal and adult male and female rats, the authors performed electrophysiological recordings in the IC of anesthetized animals. Their analyses examine differences in spontaneous activity, evoked responses to contextual sounds, neuronal mismatch components, and adaptation across sex, age, and valproic acid (VPA) exposure groups. The experiments are well-structured, and the group comparisons are clearly presented. However, the study's broader significance is not fully articulated, particularly in terms of its relevance to autism-related behaviors. Specific concerns are outlined below.

Major

1. There is no assessment of how these electrophysiological differences relate to behavioral outcomes, including both autism-related and non-related behaviors, across sex, age, and exposure groups.

2. Recordings were performed in anesthetized rats, it further raises the concern on how these findings link to behaviors.

3. It is not clear whether single units included in the analyses are all excitatory or combined with inhibitory units. If it is combined, it would be interesting to reveal whether excitatory and inhibitory units display differently in these analyses.

4. Some key details are missing from the Results and Methods sections. For instance, the explanation of the power-law model lacks sufficient clarity. The authors should elaborate on the biological relevance of power-law adaptation and define key variables such as y and t, as well as the meaning of "averaged spikes". Additionally, the statement, "These results suggest that prenatal VPA exposure alters the processing of stimulus statistics over multiple time scales in the IC," is vague, as "stimulus statistics" is not clearly defined. Providing a more explicit explanation would strengthen the interpretation of the results. Furthermore, the calculation of the half-adaptation measure is not clearly described, making it difficult to assess its significance.

Minor:

1. Neuron numbers were provided in the text of the result section, but with no information on associated animal numbers.

2. Figure 2 right panel, there are 4 smaller plots next to the schematic drawing. Not sure what they are (no legend for them). Same for Figure 3A&4A.

3. Figure 3, CTR was used in figure panels, but CAS was used in the legend.

4. Figure 6, what is "TD" groups? No information in the legend and text.

# Author comment: Author response to Decision Letter 2

**DOI:** 10.1371/journal.pbio.3003309.r003

14 May 2025

Attachment Submitted filename: response to reviewers final 12-5-25.pdf

# Peer review: Decision Letter 2

**DOI:** 10.1371/journal.pbio.3003309.r004

**Authors:** Christian Schnell, PhD

23 Jun 2025

Dear Manuel,

Thank you for your patience while we considered your revised manuscript "Atypical predictive coding in the inferior colliculus of the valproic acid-induced rat model of autism: Sex, age and exposure differences" for publication as a Research Article at PLOS Biology. This revised version of your manuscript has been evaluated by the PLOS Biology editors, the Academic Editor and the original reviewers.

Based on the reviews and on our Academic Editor's assessment of your revision, we are likely to accept this manuscript for publication, provided you satisfactorily address the remaining points raised by the reviewers. To address Reviewer 3 concerns, we encourage you to add a discussion about these concerns in the Limitations section of your manuscript.

Please also make sure to address the following data and other policy-related requests.

* We would like to suggest a different title to improve its accessibility for our broad audience:

Contextual auditory processing in the inferior colliculus is affected in a sex- and age-dependent manner in the valproic-acid-induced rat model of autism

* Please add the links to the funding agencies in the Financial Disclosure statement in the manuscript details.

* DATA POLICY:

You may be aware of the PLOS Data Policy, which requires that all data be made available without restriction: http://journals.plos.org/plosbiology/s/data-availability. For more information, please also see this editorial: http://dx.doi.org/10.1371/journal.pbio.1001797

Note that we do not require all raw data. Rather, we ask that all individual quantitative observations that underlie the data summarized in the figures and results of your paper be made available in one of the following forms:

1) Supplementary files (e.g., excel). Please ensure that all data files are uploaded as 'Supporting Information' and are invariably referred to (in the manuscript, figure legends, and the Description field when uploading your files) using the following format verbatim: S1 Data, S2 Data, etc. Multiple panels of a single or even several figures can be included as multiple sheets in one excel file that is saved using exactly the following convention: S1_Data.xlsx (using an underscore).

2) Deposition in a publicly available repository. Please also provide the accession code or a reviewer link so that we may view your data before publication.

Regardless of the method selected, please ensure that you provide the individual numerical values that underlie the summary data displayed in the following figure panels as they are essential for readers to assess your analysis and to reproduce it: 1B, 2AB, 3, 4A. 5, 6A, 7B. As far as I can see you have provided all these data already, but please add a reference in the corresponding figure legends where the source data can be found.

NOTE: the numerical data provided should include all replicates AND the way in which the plotted mean and errors were derived (it should not present only the mean/average values).

Please also ensure that figure legends in your manuscript include information on where the underlying data can be found, and ensure your supplemental data file/s has a legend.

Please ensure that your Data Statement in the submission system accurately describes where your data can be found.

* CODE POLICY

Per journal policy, if you have generated any custom code during the course of this investigation, please make it available without restrictions. Please ensure that the code is sufficiently well documented and reusable, and that your Data Statement in the Editorial Manager submission system accurately describes where your code can be found.

Please note that we cannot accept sole deposition of code in GitHub, as this could be changed after publication. However, you can archive this version of your publicly available GitHub code to Zenodo. Once you do this, it will generate a DOI number, which you will need to provide in the Data Accessibility Statement (you are welcome to also provide the GitHub access information). See the process for doing this here: https://docs.github.com/en/repositories/archiving-a-github-repository/referencing-and-citing-content

As you address these items, please take this last chance to review your reference list to ensure that it is complete and correct. If you have cited papers that have been retracted, please include the rationale for doing so in the manuscript text, or remove these references and replace them with relevant current references. Any changes to the reference list should be mentioned in the cover letter that accompanies your revised manuscript.

In addition to these revisions, you will need to complete some formatting changes, which you will receive in a follow up email. A member of our team will be in touch with a set of requests shortly.

We expect to receive your revised manuscript within two weeks.

To submit your revision, please go to https://www.editorialmanager.com/pbiology/ and log in as an Author. Click the link labelled 'Submissions Needing Revision' to find your submission record. Your revised submission must include the following:

- a cover letter that should detail your responses to any editorial requests, if applicable, and whether changes have been made to the reference list

- a Response to Reviewers file that provides a detailed response to the reviewers' comments (if applicable, if not applicable please do not delete your existing 'Response to Reviewers' file.)

- a track-changes file indicating any changes that you have made to the manuscript.

NOTE: If Supporting Information files are included with your article, note that these are not copyedited and will be published as they are submitted. Please ensure that these files are legible and of high quality (at least 300 dpi) in an easily accessible file format. For this reason, please be aware that any references listed in an SI file will not be indexed. For more information, see our Supporting Information guidelines:

https://journals.plos.org/plosbiology/s/supporting-information

*Published Peer Review History*

Please note that you may have the opportunity to make the peer review history publicly available. The record will include editor decision letters (with reviews) and your responses to reviewer comments. If eligible, we will contact you to opt in or out. Please see here for more details:

https://plos.org/published-peer-review-history/

*Press*

Should you, your institution's press office or the journal office choose to press release your paper, please ensure you have opted out of Early Article Posting on the submission form. We ask that you notify us as soon as possible if you or your institution is planning to press release the article.

*Protocols deposition*

To enhance the reproducibility of your results, we recommend that if applicable you deposit your laboratory protocols in protocols.io, where a protocol can be assigned its own identifier (DOI) such that it can be cited independently in the future. Additionally, PLOS ONE offers an option for publishing peer-reviewed Lab Protocol articles, which describe protocols hosted on protocols.io. Read more information on sharing protocols at https://plos.org/protocols?utm_medium=editorial-email&utm_source=authorletters&utm_campaign=protocols

Please do not hesitate to contact me should you have any questions.

Sincerely,

Christian

Christian Schnell, PhD

Senior Editor

cschnell@plos.org

PLOS Biology

-------------------------------------

Reviewer remarks:

Reviewer #1: I have reviewed the revised manuscript. The authors have made substantial improvements to the manuscript: they have added a hierarchical analysis which was very much needed, they have discussed more thoroughly the weaknesses in the manuscript regarding the lack of formal behavioral assessments and the use of anaesthetised animals, they have re-oriented the violin plots and now provide numbers of rats as well as units. All of these changes have added substantial clarity to what was already a very important manuscript. I have no further suggestions for this manuscript.

Reviewer #2: This revised manuscript is much improved, and the authors have put considerable effort into addressing the main reviewer concerns of statistical methodology and narrative structure. The revised manuscript performs a much more thorough and rigorous statistical analysis and completely reworks the results section and figures to first highlight population and group level comparison before delving into all the potential interactions. The discussion section also does a better job of putting results in bigger picture context, though there are a few points that could be expanded on or made clearer. While it is still a dense read, the authors did a commendable job in making the findings from this study more interpretable give the challenging and complex nature of the data set. The overarching conclusion is somewhat unsatisfying (that age, sex, and VPA exposure effect contextual sound processing in the IC in a complex and interconnected manner), but there are some specific take home points that this reviewer found to be interesting and informative, if somewhat counterintuitive.

Major comments

The authors did a commendable job in re-working the figures. One further suggestion to improve interpretability would be to consistently label all axis, violin plots (FpC, FaC, MpC…), and color-coded groups (CAS/DEV/STD or iMM/iRS/iPE). Because there are so many groups/variables that need to be kept straight, having labels on each subplot (while perhaps not the most aesthetically pleasing choice) would greatly facilitate interpretation of the graphs.

In terms of the effect of VPA exposure and autism, the major finding from this study was that VPA exposure reduced mismatch processing, with an in increase iRS but reduced iPE (at least for high sound levels in the non-lemniscal IC). The authors' interpret these results in the context of hypo-priorism in ASD, as attenuated priors would be expected to produce less robust prediction errors. This makes sense, but the logic as to how reduced priors would lead to greater iRS was unclear to me, as one may expect reduced rather than enhanced habituation if sensory input is treated as less predictable. Indeed, previous results in other ASD models have found impaired adaptation to repetitive sensory input (perhaps most notably in Fragile X Syndrome, see Ethridge et al., 2016; Lovelace et al., 2016; He et al., 2017). How can these results be reconciled?

Perhaps the most interesting finding from this study is that VPA exposure causes female specific deficits in IC development and contextual sound processing in the IC. The authors provide some context for these findings in terms of how they relate to other results from the VPA model, but this is an interesting finding in light of male prevalence of ASD and should be discussed more explicitly.

Minor comments

Line 50 typo "well-stablished"

Line117-143 This paragraph is rather long and should probably be split into two. Sentence starting with "We performed single-unit recordings.." (line 129) seems like a natural place for this.

Line 135-136: "In utero administration of VPA in pregnant rats induced autism-like signs in the offspring." Not sure if enough data is provided to support this claim.

Line 165 cite figure 1D at end of sentence

Line 193/255/405: typo, "We fitted" should be "We fit"

Lines 213-222: Various words are used in this section to describe spontaneous rates (scores, values, responses), making it somewhat confusing. Sticking with spontaneous rates or spontaneous activity levels would make this section clearer.

Line 282 p value typo (says significant sex x age x exposure but p = 0.14)

Line 339-340 "Among adult females, VPA exposure reduced DEV response compared to controls in the lemniscal division at high-level stimulation" This doesn't seem to match the data in Figure 4 lemniscal, high level plot, where FaV distribution appears to be higher than FaC.

Fig 7: It wasn't clear why only subset of conditions are looked at here. Was this due to inadequate power law fits for these data sets? May be worth showing an example of response that doesn't follow power law adaptation (e.g. for VPA groups).

Fig 8: Not sure I agree with the interpretation of these results, seems like it's more of a difference in starting point than in adaptation rate. Is there a way to quantify this (e.g. curve fitting or response ratio)?

Line 689 typo: "impaired maturation of high-spontaneous-rate in the auditory nerve fibers"

Lines 718-735. In reference to Valerio et al., the authors state that this study found that neuronal mismatch is already mature in lemniscal IC by prepurberty (lines 719-720) but then state that this study found a decrease in neuronal mismatch with age up to P50 in lemniscal IC (724-725). These statements seem contradictory, unless I am misinterpreting something.

Line 759-761 "our single-unit findings, together with behavioral evidence, reveal a mechanistic link between atypical predictive processing and core sensory and behavioral features of autism." This feels like too strong of a statement, given no behavioral phenotyping was done and no direct correlation between altered mismatch processing and repetitive behaviors was performed.

Reviewer #3: I acknowledge the technical issues in response to my last review comments. However, my concern regarding the significance of the study remains. It is still unclear which of the reported electrophysiological differences are specifically related to autism, and which may simply reflect characteristics of the animal model that are unrelated to autism-like behaviors.

# Author comment: Author response to Decision Letter 3

**DOI:** 10.1371/journal.pbio.3003309.r005

26 Jun 2025

Attachment Submitted filename: Response to reviewers.docx

# Editor report: Decision Letter 3

**DOI:** 10.1371/journal.pbio.3003309.r006

**Authors:** Christian Schnell, PhD

9 Jul 2025

Dear Manuel,

Thank you for the submission of your revised Research Article "Contextual auditory processing in the inferior colliculus is affected in a sex- and age-dependent manner in the valproic acid-induced rat model of autism" for publication in PLOS Biology. On behalf of my colleagues and the Academic Editor, Jennifer Bizley, I am pleased to say that we can in principle accept your manuscript for publication, provided you address any remaining formatting and reporting issues. These will be detailed in an email you should receive within 2-3 business days from our colleagues in the journal operations team; no action is required from you until then. Please note that we will not be able to formally accept your manuscript and schedule it for publication until you have completed any requested changes.

While you attend to those requests to come, please also make sure to make any custom scripts generated in this study available without restrictions as per journal policy. Please ensure that the code is sufficiently well documented and reusable, and that your Data Statement in the Editorial Manager submission system accurately describes where your code can be found.

Please note that we cannot accept sole deposition of code in GitHub, as this could be changed after publication. However, you can archive this version of your publicly available GitHub code to Zenodo. Once you do this, it will generate a DOI number, which you will need to provide in the Data Accessibility Statement (you are welcome to also provide the GitHub access information). See the process for doing this here:

Please take a minute to log into Editorial Manager at http://www.editorialmanager.com/pbiology/, click the "Update My Information" link at the top of the page, and update your user information to ensure an efficient production process.

PRESS

We frequently collaborate with press offices. If your institution or institutions have a press office, please notify them about your upcoming paper at this point, to enable them to help maximise its impact. If the press office is planning to promote your findings, we would be grateful if they could coordinate with biologypress@plos.org. If you have previously opted in to the early version process, we ask that you notify us immediately of any press plans so that we may opt out on your behalf.

We also ask that you take this opportunity to read our Embargo Policy regarding the discussion, promotion and media coverage of work that is yet to be published by PLOS. As your manuscript is not yet published, it is bound by the conditions of our Embargo Policy. Please be aware that this policy is in place both to ensure that any press coverage of your article is fully substantiated and to provide a direct link between such coverage and the published work. For full details of our Embargo Policy, please visit http://www.plos.org/about/media-inquiries/embargo-policy/.

Thank you again for choosing PLOS Biology for publication and supporting Open Access publishing. We look forward to publishing your study. 

Sincerely, 

Christian

Christian Schnell, PhD

Senior Editor

PLOS Biology

cschnell@plos.org
